# Bio-inspired relay catalysis for aqueous redox flow batteries

**DOI:** 10.1038/s41467-026-73670-4

**Published:** 2026-05-27

**Authors:** Jiafeng Lei, Yaqin Zhang, Weixing Wu, Ying Wang, Jun Fan, Yi-Chun Lu

**Affiliations:** 1 Electrochemical Energy and Interfaces Laboratory, Department of Mechanical and Automation Engineering, The Chinese University of Hong Kong, Shatin, Hong Kong SAR China; 2 Department of Materials Science and Engineering, City University of Hong Kong, Kowloon, Hong Kong SAR China; 3 Department of Chemistry, The Chinese University of Hong Kong, Shatin, Hong Kong SAR China

**Keywords:** Batteries, Batteries

## Abstract

Aqueous redox flow batteries are promising for long-duration energy storage. However, many of them (e.g. sulfur-based and organic-based flow batteries) suffer from sluggish kinetics with low energy efficiency and insufficient capacity utilization. Here, we propose relay catalysis as a universal strategy to achieve high reaction rates while minimizing overpotential, enabling high capacity and energy efficiency. Inspired by sequential electron transfer in cellular respiration, relay catalysis employs a low-overpotential catalyst (e.g., isoalloxazine) to initiate the reaction, seamlessly transferring control to a high-activity catalyst (e.g., quinone) to sustain charge propagation, breaking the trade-off between overpotential and catalytic rate. Using this strategy, we demonstrate polysulfide-ferrocyanide flow batteries with near full polysulfide utilization (S_4_^2–^/S_2_^2–^, 64 Ah L^–1^_negolyte_) and high stability over 3 months (> 500 cycles at 20 mA cm^–2^, decay rate 0.00071% per cycle, 0.003% per day). We further extend this strategy to organosulfide- and azo-based batteries with various relay-catalyst couples. By mimicking biological electron relays, this approach not only redefines homogeneous catalysis for energy storage but also establishes a transformative platform for designing flow batteries with enhanced performance and scalability.

## Abstract (web summary)

Aqueous redox flow batteries are promising for large-scale energy storage, but many suffer from sluggish kinetics. Here, authors report a relay catalysis strategy to break the trade-off between overpotential and catalytic rate in sulfur- and organic-based flow cells.

## Introduction

Aqueous redox flow battery (RFB) is one of the most promising long-duration energy storage technologies for integrating intermittent renewable energy into the grid power owing to its intrinsic safety and design flexibility in power and energy^1–3^. All-vanadium RFB is the most mature technology; however, its further employment is hindered by vanadium element scarcity, volatile high price, and toxicity^4,5^. Exploring RFB systems with earth-abundant elements (e.g., sulfur-based RFBs and organic-based RFBs) could substantially reduce active material cost with high sustainability^6–12^. For instance, the annual yield of sulfur is over 60,000,000 tons per year (vs. 100,000 tons per year for vanadium), while the price is 1000 times cheaper than vanadium, suggesting they are a more sustainable choice for the growing energy storage market. Organic molecules, composed of earth-abundant elements like C, H, O, N, etc., are another potentially inexpensive alternative^13,14^. However, many competitive low-cost candidates suffer from sluggish kinetics, resulting in limited capacity utilization and low operating current density. For example, polysulfide-based RFB shows a low volumetric capacity (6–13 Ah L^−1^_negolyte_ with polysulfide utilization less than 35%) and low energy efficiency (EE, 50–60%)^15–17^. While utilizing polysulfide electrolyte at a low state of charge (SOC) could increase energy efficiency (81%), it largely limits energy density (1.6 Ah L^−1^_posolyte+negolyte_)^18^. Various organic molecules like azo compound, alloxazine, organosulfide, and fluorene molecules are also suffering from sluggish kinetics with limited capacity utilization and high voltage gap with low operating current^19–22^.

Developing effective catalytic processes and low-cost catalysts is critical for enabling the exploitation of earth-abundant, low-cost active materials in RFBs. Taking polysulfide-based RFB as an example, solid catalysts have been applied to improve the kinetics, including various metal sulfides (e.g., CoS/CoS_2_, NiS_x_, CuS_x_, WS_2_) and doped-carbon (e.g., nitrogen-doped carbon) catalysts^17,23–27^. However, their effectiveness remains unsatisfactory due to limited reaction sites, catalyst exfoliation, side reactions, and poor catalytic activity^15,19,28,29^. Homogeneous catalysis, in which both the catalysts and active material are all soluble in the electrolyte to flow, is a promising approach to address these issues in RFBs. For instance, Feng et al. reported the use of β-cyclodextrin as the redox-inactive organic additive to regulate alcohol oxidation of fluorenone-based RFB^20^. We previously introduced a molecular catalyst riboflavin sodium phosphate (FMN-Na), an isoalloxazine derivative, to accelerate the polysulfide reduction reaction by transferring the sluggish electrochemical reaction to a fast chemical reaction via homogeneous catalysis^30^. However, the FMN-catalyzed polysulfide-based RFB only showed limited polysulfide utilization (33%) in long-term operation due to insufficient catalytic rate at a high SOC (i.e., low polysulfide concentration). Exploring molecular catalysts with higher electron transfer rates is a feasible way to enhance capacity utilization. However, it is commonly observed that the molecules with high electron transfer rates usually bring a larger overpotential with a large potential difference with the reactants as the driving force of catalytic reaction in lithium-air batteries, redox-mediated batteries, and homogeneous catalysis applicaitons^31–36^, highlighting a tradeoff between electron transfer rate and overpotential and challenges in the practical engineering field under operating conditions.

**Fig. 1:**
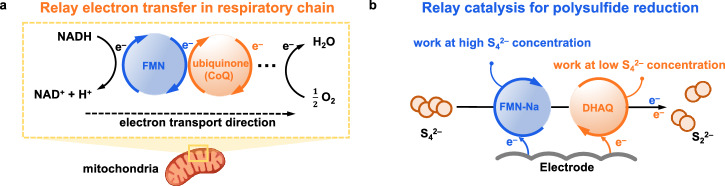
The design principle for bio-inspired relay catalysis on polysulfide reduction. **a** The schematic illustration of the electron transfer process in the biological respiratory chain. **b** The schematic illustration of the relay catalysis strategy of reducing K_2_S_4_ in a relay method, in which FMN takes effect on a high S_4_^2−^ concentration and the activating catalyst is switched to DHAQ at a low S_4_^2−^ concentration.

Herein, inspired by the sequential electron transfer in the cellular respiration chain, we employ a quinone-based molecular catalysts and propose a relay catalysis strategy to achieve both high capacity and low overpotential in aqueous redox flow batteries. In nature, electron stepwise relay is a common phenomenon in living cells, including energy production processes like photosynthesis and cellular respiration^37,38^. Taking respiratory chain as an example, the electron released from nicotinamide adenine dinucleotide is not directly oxidized within one step but transferred to flavin mononucleotide (FMN) followed by ubiquinone which then passes the electrons to *cytochrome c*^39,40^ (Fig. 1a). The electron transfer continues until the electron reaches the terminal acceptor, oxygen, and the series of electron transfer process resemble a relay race driven by potential difference. In this relay process, the electrons transfer through the lipid membrane and establish the proton gradient at the cost of the redox potential difference between each relay process, with a total energy efficiency of around 70%^41–43^. In cells, this rate-controlled mechanism allows precise energy production based on dynamic energy consumption and avoids the generation of deleterious reactive dioxygen species^41^. This natural process inspires us in two aspects: firstly, ubiquinone being an ideal electron receiver from FMN and electron donor of *cytochrome c*, suggests that quinone-based molecules (with suitable reversible potential) could be active electron shuttles for catalysis in flow batteries; secondly, the relay mode is an efficient way to take full advantage of catalysts with different reversible potential and catalytic activity on sequences by design to minimize energy loss.

Inspired by the electron-relay process in the natural respiratory process, we introduced an anthraquinone derivative, 2,6-dihydroxyanthraquinone (2,6-DHAQ) having a more negative potential but higher electron transfer rate compared with FMN-Na, as the relay catalyst to cooperate with FMN-Na to achieve the full polysulfide utilization with minimum energy loss in polysulfide-based RFB (Fig. 1b). Compared with using 2,6-DHAQ (high reaction rate but high overpotential) and FMN-Na (low reaction rate but low overpotential) alone, the cooperative mechanism, which mimics the sequential electron transfer in the cellular respiration chain, break the tradeoff between catalytic rate and overpotential. This relay mode minimizes energy loss by firstly utilizing FMN-Na at a lower overpotential and switching to 2,6-DHAQ to avoid premature termination of the charging process. The high-concentration polysulfide-ferrocyanide (S-Fe) RFB showed near full polysulfide utilization (S_4_^2−^/S_2_^2−^), yielding a high volumetric capacity of 64 Ah L^−1^_negolyte_ (near 100% SOC) for over 3 months (over 500 cycles) with a low decay rate of 0.00071% per cycle and 0.003% per day. The relay process, i.e., active catalysis transitioning from FMN-Na to 2,6-DHAQ, was visualized by operando ultraviolet–visible light (UV–vis) spectroscopy, providing insights into the relay catalysis for polysulfide reduction reactions. We successfully applied the relay catalysis strategy to a two-layer, 100 cm^2^ S-Fe flow cell stack that operates for more than 200 cycles (over 315 h) at 5 A to show its scalability. We further verified the relay catalysis strategy for other active materials, including organosulfides and azo compounds with various relay-catalyst couples, greatly broadening the scope of homogeneous catalysis in the redox flow batteries.

## Results

### Molecular catalysts with high electron transfer rate for polysulfide RFB

Sulfur, a byproduct of petroleum processes, has gained significant attention as the active material for rechargeable batteries like polysulfide-based RFBs owing to its low cost for energy storage^3,44–46^. The charge storage mechanism of polysulfide-based batteries relies on reversible formation/cleavage of sulfur–sulfur bonds (S–S), accompanied by the reversible conversion between long-chain polysulfide and short-chain polysulfide (Eq. (1))^15,16^.

1 $${{{{\rm{S}}}}}_{4}^{2-}+2\,{{{{\rm{e}}}}}^{-}\leftrightarrow 2\,{{{{\rm{S}}}}}_{2}^{2-}$$

Polysulfide-based RFBs have been suffering from sluggish kinetics of polysulfide reduction reaction involving the S–S bond cleavage process, resulting in low energy efficiency and low polysulfide utilization^15,29^. Developing molecular catalysts with high electron transfer rate is the key to achieve high capacity with full polysulfide utilization.

In nature, quinone molecules are among the most common redox-active biomolecules involved in the biogeochemical process, and it was revealed that ubiquinone collaborates with FMN on the mitochondria membrane by transferring electrons^47,48^. Quinone molecules possessing a lower potential than polysulfide reduction reaction ( ~ −0.45 V versus standard hydrogen electrode (SHE) for S_4_^2−^/S_2_^2−^) could potentially serve as the second relay molecular catalysts^30,49^. A more negative potential of molecular catalysts may generate a larger potential difference and therefore, with a more negative Gibbs free energy (Eqs. (2) and (3)), providing a larger driving force for this reaction^50,51^ (detailed analysis in Supplementary Note 1).

2 $$\Delta {{{\rm{E}}}}={{{{\rm{E}}}}}_{{{{\rm{polysulfide}}}}}-{{{{\rm{E}}}}}_{{{{\rm{molecular\; catalyst}}}}}$$

3 $$\Delta {{{\rm{G}}}}=-{{{\rm{n\; F}}}}\,\Delta {{{\rm{E}}}}$$

where $\Delta$E is the potential difference of polysulfide species and molecular catalyst, $\Delta$G is the Gibbs free energy, *n* is the number of electrons transferred between molecular catalysts and polysulfide, and *F* is Faraday’s constant. In lithium-oxygen and redox-mediated batteries, it is commonly observed that the molecules exhibiting fast kinetics usually have a larger potential difference from the reactants^31,32^, which inspires us to explore molecules at lower potential range for achieving fast kinetics of polysulfide reduction reactions. Furthermore, in a reduction reaction, the low-potential catalyst (high-rate one) can cooperate with the high-potential catalyst (low-rate one) in a relay mode to minimize energy loss: the high-potential catalyst will work first at low overpotential and once its catalytic rate could not catch up with the input current due to decreased reactant concentration, the second catalyst will be activated by a negative potential shift and take over catalyzing the rest of reactants, achieving full capacity utilization. The “relay operation” in homogeneous catalysis is differentiated from the commonly reported dual catalysts (or dual mediators) that each catalyst works on different reactions^52,53^. For instance, one catalyst only works on discharge reactions, and the other one works for charge reactions. In this way, the limitation and the trade-off (between kinetics and overpotential) of the single catalyst still exist in two separate reactions. However, in our relay approach, the design principle of relay catalysis on the same reaction lies in the potential/kinetics match and the dynamic transition from the low-rate catalyst to a high-rate catalyst via concentration-triggered potential shift. This cooperative relay mechanism effectively breaks the trade-off between decreasing overpotential and increasing driving force via streamlining the electron transfer sequence of multiple catalysts, maximizing achieved capacity while minimizing energy loss.

Considering the suitable potential, high stability, and cost at the industrial level, we selected the anthraquinone derivatives as the possible candidates. Three dihydroxyanthraquinones were tested, including 1,4-dihydroxyanthraquinone (1,4-DHAQ), 1,8-dihydroxyanthraquinone (1,8-DHAQ), and 2,6-DHAQ, as the molecular catalysts (Supplementary Fig. 1) to study their reaction mechanism. In a strongly alkaline environment, the DHAQ exists predominantly in the deprotonated form^54,55^. The proposed catalysis mechanism is listed below (Eqs. (4) and (5)).

4 $${{{\rm{DHA}}}}{{{{\rm{Q}}}}}^{2-}+2\,{{{{\rm{e}}}}}^{-}\,\to \,{{{\rm{DHA}}}}{{{{\rm{Q}}}}}^{4-}\,$$

5 $${{{\rm{DHA}}}}{{{{\rm{Q}}}}}^{4-}+{{{\rm{S}}}}_{4}^{2-}\to {{{\rm{DHA}}}}{{{{\rm{Q}}}}}^{2{{{\rm{\hbox{-}}}}}}+2\,{{{{\rm{S}}}}}_{2}^{2-}$$

**Fig. 2:**
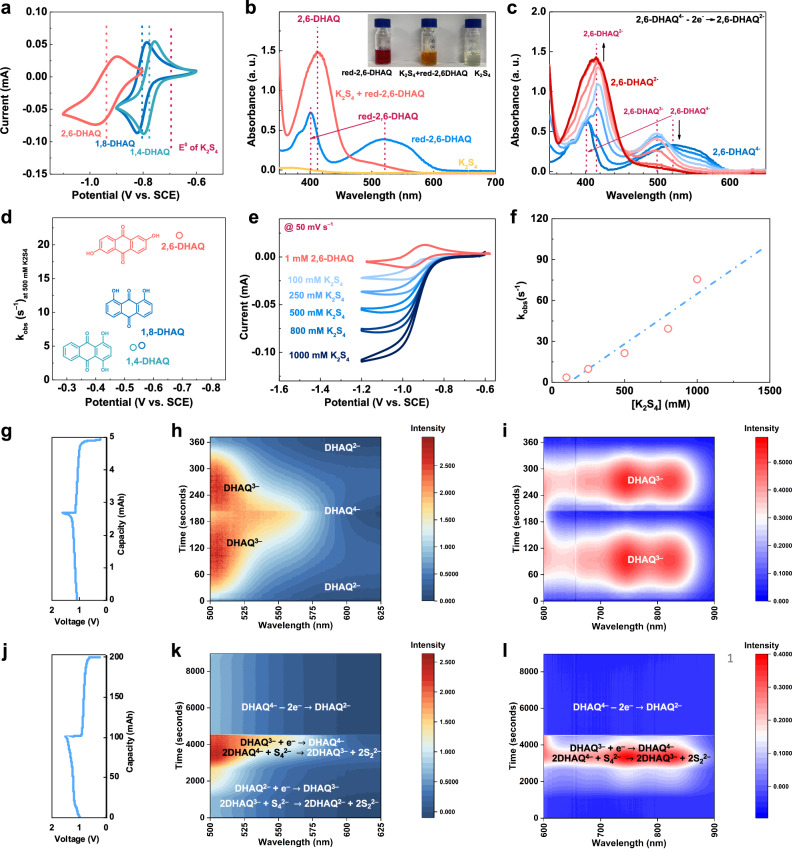
The reaction mechanism of DHAQ molecular catalysts for polysulfide reduction reaction. **a** The cyclic voltammograms of 2,6-DHAQ, 1,8-DHAQ, and 1,4-DHAQ. **b** The UV–vis spectra of 1 mM K_2_S_4_, 1 mM reduced 2,6-DHAQ (red-2,6-DHAQ), and a mixed solution of K_2_S_4_ + red-2,6-DHAQ. The inset shows the optical images of these solutions. The red dashed lines indicate the peak assignment of DHAQ^2−^ and DHAQ^4−^. **c** The operando UV–vis spectrum of 1 mM 2,6-DHAQ during oxidation. The color change from deep blue to deep red represents the evolution from the reduced state (DHAQ^4−^) to the oxidized state (DHAQ^2−^), and the spectra were collected at equal time intervals. The black arrows indicate the evolution trend. **d** The *k*_obs_ of 2,6-DHAQ, 1,8-DHAQ, and 1,4-DHAQ at 500 mM K_2_S_4_ electrolyte. **e** Successive cyclic voltammograms of 2,6-DHAQ with K_2_S_4_ electrolyte from 100 mM to 1000 mM at 50 mV s^−1^ after background correction. **f** The linear fit (*y* = 0.0739 *x*– 9.2317, *R*^2^ = 0.91) of the *k*_obs_ over K_2_S_4_ concentration. **g** The voltage profiles of 5 mM 2,6-DHAQ in a DHAQ-Fe flow cell at 4 mA cm^−2^ with 1.5 V cut-off voltage (10 mL of 5 mM 2,6-DHAQ–1 M KOH |Nafion 117 membrane| 15 mL of 0.25 M K_4_[Fe(CN)_6_]–1 M KCl). The operando UV–vis spectra of 2,6-DHAQ negolyte from **h** 500 nm to 625 nm and **i** 600 nm to 900 nm. **j** The voltage profiles of 250 mM K_2_S_4_ with 5 mM 2,6-DHAQ in a S-Fe flow cell at 20 mA cm^−2^ with 1.5 V cut-off voltage (10 mL of 0.25 M K_2_S_4_–1 M KOH with 5 mM 2,6-DHAQ |Nafion 117 membrane| 15 mL of 0.25 M K_4_[Fe(CN)_6_]–1 M KCl). The operando UV–vis spectra of K_2_S_4_ with 2,6-DHAQ negolyte from **k** 500 nm to 625 nm and **l** 600 nm to 900 nm. The operando tests were conducted at 24 ± 1 °C.

We firstly examined its electrochemical properties and characterized the spontaneous chemical reaction by UV–vis spectroscopy. The cyclic voltammograms show that 1,4-DHAQ, 1,8-DHAQ, and 2,6-DHAQ possesses a low potential of −0.53 V vs. SHE, −0.56 V vs. SHE, and −0.69 V vs. SHE, respectively, which are all lower than the theoretical potential of polysulfide species (−0.45 V vs. SHE), fulfilling the thermodynamic prerequisite (Fig. 2a and Supplementary Fig. 2).

We conducted UV–vis spectroscopy measurements to verify if reduced 2,6-DHAQ could chemically react with K_2_S_4_. Figure 2b shows that when mixing the reduced 2,6-DHAQ molecular catalyst with K_2_S_4_ electrolyte, the absorbance of 2,6-DHAQ^4−^ at 520 nm is dramatically decreased, while the peak at 401 nm is redshifted to 412 nm, and, rapidly, the red solution turned to orange (inset in Fig. 2b). These peak assignments are supported by the operando UV–vis spectra (Fig. 2c and Supplementary Figs. 3–5), collectively verifying 2,6-DHAQ could chemically reduce polysulfide species (Supplementary Note 2). Similarly, the spontaneous chemical reaction between reduced molecular molecules and K_2_S_4_ is further verified for 1,4-DHAQ and 1,8-DHAQ (Supplementary Figs. 6–8 and Supplementary Note 2), supporting the universality of this molecular catalyst strategy.

We evaluated the homogeneous electron transfer rate by measuring the turnover frequency (also called observed reaction rate, *k*_obs_)^56,57^ of various DHAQ candidates. The *k*_obs_ of 2,6-DHAQ, 1,8-DHAQ and 1,4-DHAQ are 21.4, 5.1, and 4.8 s^–1^ at 500 mM K_2_S_4_, respectively (Fig. 2d, Supplementary Figs. 9–11, and Supplementary Note 3), which is consistent with our in situ UV–vis spectra results that the 2,6-DHAQ reached the steady-state the fastest after mixing with polysulfide, followed by 1,8-DHAQ and 1,4-DHAQ, respectively (Supplementary Figs. 12–21 and Supplementary Note 4), confirming a higher catalytic rate of 2,6-DHAQ over the other two DHAQ candidates. This trend is consistent with our hypothesis that lower reversible potential of the catalyst may lead to a higher catalytic activity in the same derivative family (Fig. 2d, inset in Supplementary Fig. 19) owing to a higher driving force. The *k*_obs_ of 2,6-DHAQ is determined to be 75 s^−1^ at 1 M K_2_S_4_ electrolyte, approximately 120% higher than FMN-Na (33 s^−1^)^30^ (Fig. 2e, f). These results all verified 2,6-DHAQ is an ideal molecular catalyst with high catalytic rate for relay catalysis.

Operando UV–vis spectroscopy was conducted to investigate the 2,6-DHAQ catalytic process in the polysulfide-based flow cell during charge-discharge cycle. We firstly fabricated the 2,6-DHAQ-ferrocyanide flow cell with 2,6-DHAQ alone as the negolyte to establish the benchmark during the evolution of various DHAQ (DHAQ represents 2,6-DHAQ in the following discussion for simplicity) states (Fig. 2g–i). The absorbance at 520 nm and 744 nm are picked as the indicator of DHAQ^2−^/DHAQ^4−^ and DHAQ^3−^, respectively (Supplementary Figs. 22 and 23 and Supplementary Note 5). Interestingly, for the polysulfide-ferrocyanide cell with 2,6-DHAQ, we observed two distinct catalytic processes occurring during the polysulfide reduction process (Fig. 2j–l and Supplementary Fig. 24). In the initial range, there is barely any change in the intensity because the reduced DHAQ^3−^ are rapidly regenerated to DHAQ^2−^ with a fast reaction rate (Fig. 2k, l and Supplementary Fig. 24). However, Fig. 2l indicated a gradual accumulation of DHAQ^3−^ over charging time. At higher SOC, we observed the absorbance of DHAQ^3−^ start to decrease, while the absorbance of DHAQ^4−^ continuously increases (Fig. 2j–l and Supplementary Fig. 24). We hypothesize that these ranges correspond to two catalytic processes:

6 $$2\,{{{\rm{DHA}}}}{{{{\rm{Q}}}}}^{3-}+{{{{\rm{S}}}}}_{4}^{2-}\to 2\,{{{\rm{DHA}}}}{{{{\rm{Q}}}}}^{2-}+2\,{{{{\rm{S}}}}}_{2}^{2-}$$

7 $$2\,{{{\rm{DHA}}}}{{{{\rm{Q}}}}}^{4-}+{{{\rm{S}}}}_{4}^{2{{{\rm{\hbox{-}}}}}}\to 2\,{{{\rm{DHA}}}}{{{{\rm{Q}}}}}^{3-}+2\,{{{{\rm{S}}}}}_{2}^{2-}$$

In the first region with high polysulfide concentration, the reaction rate of Eqs. (6) and (7) is fast enough to support the fast regeneration of DHAQ^2−^. However, as the SOC increases, the reaction rate of Eq. (6) declines and becomes the rate-determining step, leading to the accumulation of DHAQ^3−^. This result indicates Eq. (7) (DHAQ^3−^ regeneration rate) has a higher reaction rate than Eq. (6) (DHAQ^3−^ consumption rate), otherwise no DHAQ^3−^ should be detected. The DHAQ^4−^ has a higher electron transfer rate (Eq. (7)). During discharge, the absorbance of 2,6-DHAQ changes back to the initial state and remain stable in the subsequent discharging process. The operando UV–vis spectra suggested two catalyst molecules would combine with one polysulfide chain to break the disulfide bond (Eqs. (6) and (7)).

**Fig. 3:**
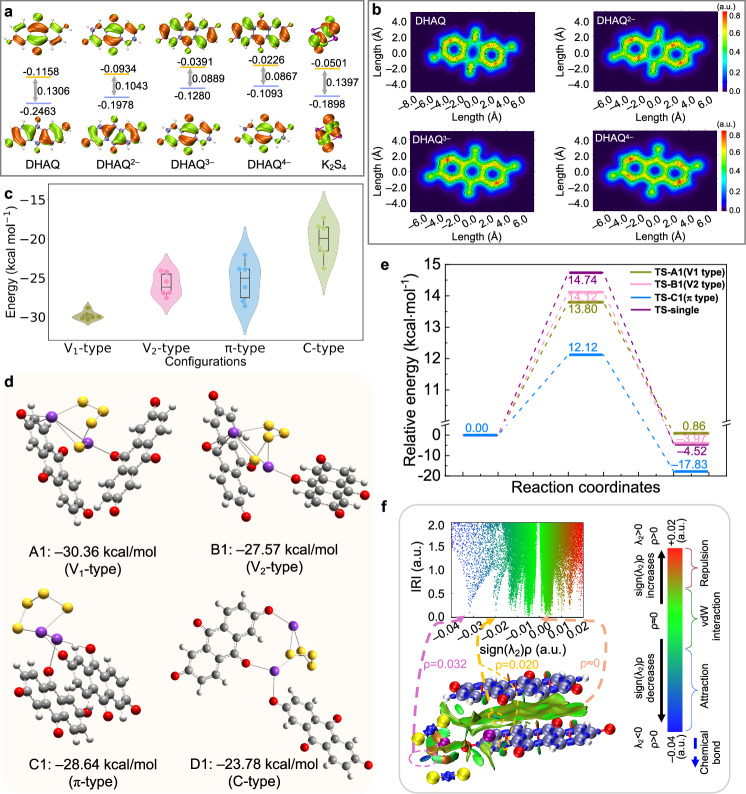
Theoretical calculations on the reaction mechanism of 2,6-DHAQ and K_2_S_4_. **a** Frontier molecular orbitals of DHAQ, reduced DHAQ, and K_2_S_4_. The energy levels are in a.u. **b** Projected localized orbital locator-π (LOL-π) map of DHAQ and its reduced states. **c** Violin distribution of interaction energy between K_2_S_4_ and two DHAQ^4−^ molecules, forming V_1_-type, V_2_-type, π-type, and C-type configurations. Six configurations are calculated for each combination type. Box plots inside violins show the median, lower (Q1) and upper (Q3) quartiles, with whiskers extending to the upper and lower percentiles, and the lower and upper limits defined as 1.5× the interquartile range. **d** Representative combinations between two DHAQ^4‒^ molecules and K_2_S_4_. Atoms are color-coded as follows: C (grey), H (white), O (red), S (yellow), K (purple). **e** Comparison of reaction energy barriers of cleavage of disulfide (–S–S–) bonds catalyzed by two reduced DHAQ^4−^ or single DHAQ^4−^ and K_2_S_4_ through TS-A1 (V_1_-type), TS-B1 (V_2_-type), TS-C1 (π-type), and TS-single configurations. **f** Visualizing chemical bond and non-bond regions by interaction region indicator (IRI) analysis of the transition state of π-type (TS-C1) intermediate.

We performed density functional theory (DFT) calculations to understand the reaction mechanism of the polysulfide catalytic process. We analyze the electronic property of 2,6-DHAQ. The highest occupied molecular orbitals spread around the aromatic rings and the active oxygen atoms of 2,6-DHAQ^4‒^ (Fig. 3a), whose conjugation effects with a π-character are presented by the localized orbital locator-π map (Fig. 3b and Supplementary Note 6). It is believed that these π-electrons can favor short-range π–π stacking between aromatic rings, providing electron migration channels^58^.

To investigate the reaction kinetics and energy barrier of polysulfide reduction reactions, we first calculate the possible combinations between 2,6-DHAQ^4‒^ and K_2_S_4_. We divide the possible configuration into four types according to the angle (*θ*) between two 2,6-DHAQ planes and the distance (*L*) between geometric center of two 2,6-DHAQ molecules (Supplementary Table 1 and Supplementary Figs. 25–28): V_1_-type (*θ* < 90°), V_2_-type (*θ* > 90°), π-type (*θ* < 5°, π–π stacking between DHAQ planes), and C-type (*L* > 10 Å, chain-like connection), whose interaction energies and representative configurations are shown in Fig. 3c, d. The oxygen atoms of 2,6-DHAQ play a key role in all the conformations, suggesting the favor combination site. The energy barriers through V_1_-type, V_2_-type, and π-type binding are 13.80, 14.12, and 12.12 kcal/mol, respectively (Fig. 3e and Supplementary Figs. 29–31). These values are all lower than that of single 2,6-DHAQ^4‒^ catalyzed reaction (14.74 kcal/mol, Fig. 3e and Supplementary Fig. 32). Furthermore, the closer the distance between two 2,6-DHAQ^4‒^ molecules, the lower the energy barriers, confirming the importance of π–π stacking effects (12.12 kcal/mol for π-type) in lowering the reaction barrier and stabilizing the intermediates. We visualize the cleavage of the S‒S bond (purple circle) and the van der Waals interaction (green surfaces) by real space function named interaction region indicator (IRI)^59^ in Fig. 3f, suggesting an enhancement on the overall kinetics of disulfide bond cleavage by π–π channels (Supplementary Figs. 33 and 34 and Supplementary Note 7). Our results emphasized that not only the interactions between molecular catalysts and polysulfide are vital, but also the interactions between molecular catalysts themselves play an important role on the polysulfide catalytic process.

### Relay catalysis in S-Fe flow cells with high capacity and low overpotential

**Fig. 4:**
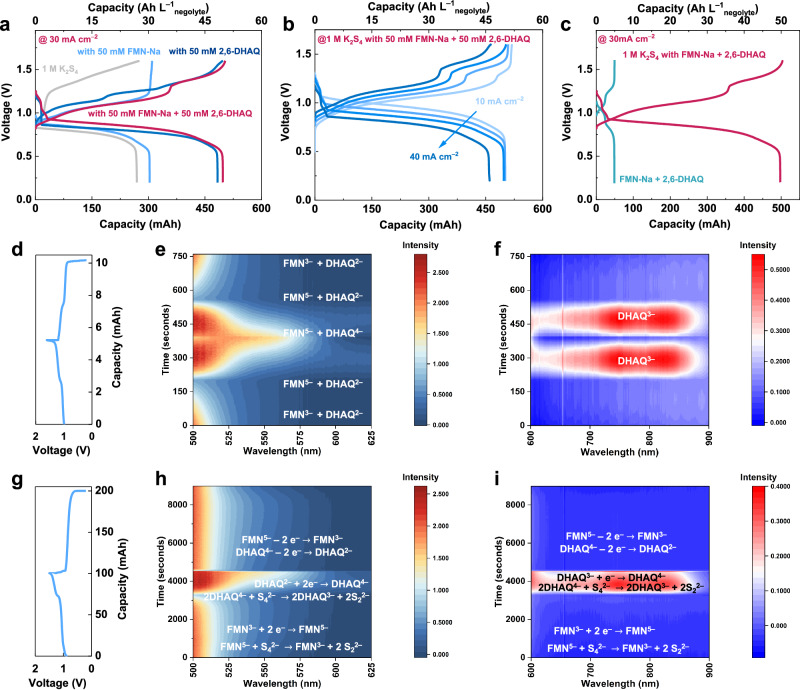
Electrochemical performance and operando UV–vis characterization of polysulfide-ferrocyanide flow cell with or without relay catalysts. **a** The voltage profiles of S-Fe flow cells with or without relay catalysts at 30 mA cm^−2^ (10 mL of 1 M K_2_S_4_–1 M KOH with or without molecular catalysts |Nafion 117 membrane| 40 mL of 0.5 M K_4_[Fe(CN)_6_]–1 M KCl). **b** The voltage profiles of S-Fe flow cells with 50 mM FMN-Na + 50 mM 2,6-DHAQ from 10 to 40 mA cm^−2^ (10 mL of 1 M K_2_S_4_ with 50 mM FMN-Na and 50 mM 2,6-DHAQ–1 M KOH |Nafion 117 membrane| 40 mL of 0.5 M K_4_[Fe(CN)_6_]–1 M KCl). **c** The capacity contribution of FMN-Na + 2,6-DHAQ relay catalysts at 30 mA cm^−2^. **d** The voltage profiles of 5 mM FMN-Na + 5 mM 2,6-DHAQ in a DHAQ + FMN-Fe flow cell at 4 mA cm^−2^ (10 mL of 5 mM FMN-Na and 5 mM 2,6-DHAQ –1 M KOH |Nafion 117 membrane| 15 mL of 0.25 M K_4_[Fe(CN)_6_]–1 M KCl). The operando UV–vis spectra of 2,6-DHAQ + FMN-Na negolyte from **e** 500 to 625 nm and **f** 600 to 900 nm. **g** The voltage profiles of 250 mM K_2_S_4_ with 5 mM FMN-Na and 5 mM 2,6-DHAQ in a S-Fe flow cell at 20 mA cm^−2^ (10 mL of 0.25 M K_2_S_4_–1 M KOH with 5 mM FMN-Na and 5 mM 2,6-DHAQ |Nafion 117 membrane| 15 mL of 0.25 M K_4_[Fe(CN)_6_]–1 M KCl). The operando UV–vis spectra of K_2_S_4_ with FMN-Na and 2,6-DHAQ negolyte from **h** 500 to 625 nm and **i** 600 to 900 nm. The operando tests were conducted at 24 ± 1 °C.

We fabricated polysulfide-ferrocyanide (S-Fe) RFBs (Supplementary Fig. 35) to study the relay-catalysis effect compared with single molecular catalyst. A harsh capacity-balanced condition^2,60^ is employed (capacity of posolyte: negolyte = 1:1). The results shown in Fig. 4a (voltage profiles at 30 mA cm^−2^) verified that the S-Fe RFBs with relay molecular catalysts (FMN-Na + 2,6-DHAQ) successfully increased the achievable capacity without sacrificing energy efficiency compared to the single FMN-Na or 2,6-DHAQ catalyst. The results shown in Fig. 4b and Supplementary Fig. 36 compare them in all current densities. Without molecular catalysts, the S-Fe RFB exhibits a high overpotential due to the sluggish kinetics of polysulfide redox, and the high overpotential leads to early termination of the charging process (grey line, Fig. 4a) and thus a low-capacity utilization of 49% at 30 mA cm^−2^. The premature charging termination is more obvious at higher current density (terminated at around 20 Ah L^−1^_negolyte_ with a low-capacity utilization of 38% and a low EE of 48.1% at 40 mA cm^−2^).

Using FMN-Na alone, the S-Fe RFB showed a much-reduced overpotential but only achieved a volumetric capacity of 30 Ah L^−1^_negolyte_ at 30 mA cm^–2^ (light blue line, Fig. 4a). The limited polysulfide utilization of FMN-Na catalysts is consistent with our operando UV–vis study that the insufficient catalytic rate of FMN-Na leads to pre-termination of the charging process and low polysulfide utilization (Supplementary Figs. 37–41 and Supplementary Note 8). Interestingly, after introducing 2,6-DHAQ into the negolyte, it nearly achieved its theoretical volumetric capacity of 50 Ah L^−1^_negolyte_ with the presence of the second plateau (red line, Fig. 4a). Note that the capacity of 2,6-DHAQ itself was only 2.3 Ah L^−1^_negolyte_ (Supplementary Fig. 42a). Compared with using FMN-Na alone, the FMN-Na + 2,6-DHAQ relay catalysis increased capacity utilization by 67% at 30 mA cm^−2^ with comparable energy efficiency (70.34% vs. 71.96%, Supplementary Fig. 43). Since most of the capacity is achieved at the low-voltage plateau by FMN-Na, the energy efficiency loss at the high-voltage range is minimized. The S-Fe RFB using 2,6-DHAQ alone showed a high volumetric capacity but a high overpotential with a low EE of 61.4% (navy blue line, Fig. 4a), which is lower than the S-Fe RFB with relay catalysis (70.3%). Such a comparison highlights the significance of the relay mechanism in maintaining high energy efficiency, compared with a single high-overpotential catalyst (2,6-DHAQ). At 1.2 M K_2_S_4_ concentration, the volumetric capacity is boosted to 63 Ah L^−1^_negolyte_ with an EE of 72.3% at 30 mA cm^−2^ (Supplementary Fig. 44). The lower CE at higher K_2_S_4_ concentration can be attributed to higher polysulfide crossover rate and a higher E/P ratio (more time for a single cycle). We compare 25 mM FMN + 25 mM DHAQ with 50 mM DHAQ and 50 mM FMN. As shown in Supplementary Fig. 45, the 25 mM FMN-Na + 25 mM DHAQ relay catalysts still show better performance than the 50 mM DHAQ or 50 mM FMN-Na catalysts in terms of capacity and energy efficiency, supporting that the benefit of relay catalysis is greater than the concentration effect. We evaluate the capacity contribution from the molecular catalysts itself at all rates (Fig. 4c and Supplementary Fig. 42), indicating that the capacity contribution from relay catalysts themselves is much lower than the capacity enhancement observed in the S-Fe RFB (Fig. 4c). The four-electrode test indicated that the relay catalysts decrease the negolyte overpotential, and the second plateau is from the lower working potential of the 2,6-DHAQ on the negolyte side (Supplementary Figs. 46 and 47). The iR correction was conducted to see the contribution of the energy loss in cells with or without relay catalysts. Interestingly, the cell with relay catalysts shows greatly decreased overpotential after removing the ohmic loss, while the high overpotential could not be fully eliminated from iR correction (Supplementary Fig. 48), suggesting the main energy efficiency loss in the catalyst-assisted system is from the high resistance of Nafion 117 membrane, but the poor polysulfide kinetics for catalysts-free system. The CVs of K_2_S_4_ with relay catalysts showing two distinct plateaus corresponding to FMN at high-potential and DHAQ at low-potential, respectively, showing this two-step relay catalytic process at different potential ranges via homogeneous catalysis (Supplementary Fig. 49).

The operando UV–vis spectroscopy was conducted to study the relay catalysis process during the flow cell operation. Interestingly, the mixed electrolyte containing dual molecular catalysts showed a distinct trend in their absorbance: FMN-Na declines, while 2,6-DHAQ increases in the same wavelength range. This allowed us to distinguish the two molecular catalysts in the mixed electrolyte during charge (Fig. 4d–f, Supplementary Fig. 50, and Supplementary Note 9) and the reverse trend during discharge. For the S-Fe RFB with relay catalyst, the UV–vis absorbance of FMN^3−^ maintains during most of the initial charging stage, while it decreases quickly once the polysulfide concentration drops below a limiting concentration, at which the reaction rate of FMN could not catch up the electron input rate (Fig. 4g–i and Supplementary Fig. 51). In contrast, 2,6-DHAQ with a higher electron transfer rate start to take over the catalytic process. With the second charging plateau observed in Fig. 4g, the absorbance of DHAQ^3−^ rapidly increases (Fig. 4h, i and pink line in Supplementary Fig. 51), indicating the fast chemical reaction between reduced DHAQ^4−^ and polysulfide species (Eq. (7)). This S-Fe cell achieved 100% SOC with a low overpotential and the presence of FMN-Na greatly delays the onset of the reduction of 2,6-DHAQ (Supplementary Fig. 52). During discharge, 2,6-DHAQ and FMN-Na are oxidized first and the absorbance rapidly jumps back to its original intensity (Fig. 4g–i and Supplementary Fig. 51). In summary, at the beginning of the charge process (high S_4_^2−^ concentration, high electrode potential), FMN-Na is firstly activated to transfer electrons to polysulfide. As the reaction proceeds and polysulfide concentration reduces (low S_4_^2−^ concentration, low electrode potential), the activity of FMN-Na is insufficient to keep up with the applied current, 2,6-DHAQ is then activated to catalyze the polysulfide reduction reaction. Compared with using 2,6-DHAQ alone (high reaction rate but high overpotential), the cooperative relay mechanism minimizes energy loss by firstly utilizing FMN-Na at a lower overpotential and switching to 2,6-DHAQ to avoid premature termination of the charging process. The appearance of the extra plateau, capacity increase, and precise absorbance transition in the UV–vis spectra all verify the relay catalysis from low-rate catalysts FMN-Na to high-rate catalysts 2,6-DHAQ.

**Fig. 5:**
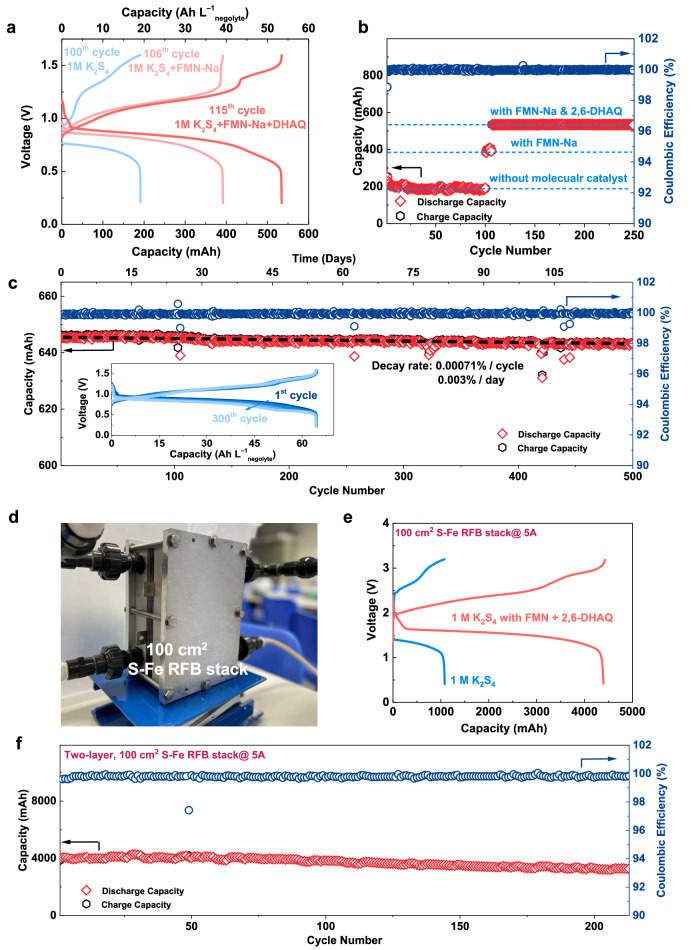
Long-term cycling stability of the S-Fe flow cells with relay catalysts and the application of relay catalysts in a 100 cm^2^ flow cell stack. **a** The voltage profiles upon stepwise addition of 50 mM FMN-Na and 50 mM 2,6-DHAQ into the un-catalyzed S-Fe flow cell after 100 cycles at 20 mA cm^−2^ (10 mL of 1 M K_2_S_4_–1 M KOH |CRIS membrane| 40 mL of 0.5 M K_4_[Fe(CN)_6_]–1 M KCl). The 50 mM FMN-Na and 50 mM 2,6-DHAQ were added at the 100^th^ cycle and 106^th^ cycle. **b** The cycling stability of the S-Fe flow cell after adding 50 mM FMN-Na and 50 mM 2,6-DHAQ. **c** The cycling stability of the high concentration S-Fe flow cell at 20 mA cm^−2^. The inset shows the voltage profiles from the 1 cycle to the 300^th^ cycle. (10 mL of 1.2 M K_2_S_4_–1 M KOH with 50 mM FMN-Na and 50 mM 2,6-DHAQ |CRIS membrane| 24 mL of 1 M ferrocyanide posolyte (0.5 M K_4_[Fe(CN)_6_] + 0.5 M Na_4_[Fe(CN)_6_])). **d** The optical images of the two-layer 100 cm^2^ flow cell stack. **e** The voltage profile comparison of the S-Fe flow cell stack at 5 A with (pink line) or without (blue line) relay catalysts. **f** The cycling stability of the two-layer, 100 cm^2^ S-Fe flow cell stack at 5 A.

The relay catalysis effect was further confirmed by stepwise adding FMN-Na and 2,6-DHAQ catalysts into the un-catalyzed S-Fe flow cell with a charge-reinforced ion-selective (CRIS) membrane^46,61^ for long-term operation. We adopted the CRIS membrane as it was reported to mitigate polysulfide crossover rate and water/OH^−^ migration with restrained membrane swelling. Due to the high overpotential and sluggish kinetics of polysulfide negolyte, only around 19 Ah L^−1^_negolyte_ is achieved in the un-catalyzed S-Fe flow cell (Fig. 5a, b). After 100 cycles cycling, 50 mM FMN-Na is added into the negolyte with the capacity increasing to 39 Ah L^−1^_negolyte_ accompanied by overpotential decrease, but the polysulfide utilization is only 73% (Fig. 5a). After 5 cycles, 50 mM 2,6-DHAQ is added to the negolyte and the cell achieved full capacity utilization of 53 Ah L^−1^_negolyte_ (Fig. 5a, b and Supplementary Fig. 53). Similar effect was observed by adding 2,6-DHAQ to the FMN-catalyzed S-Fe flow cell, after which this battery shows a capacity increase around 40% and a high stability for over additional 250 cycles (Supplementary Fig. 54). This relay strategy was further applied to other catalyst-couples with matched kinetics and potential, like quinone-quinone and quinone-phenazine relay catalysts. Supplementary Fig. 55 shows that by coupling high-potential 1,4-DHAQ and 1,8-DHAQ with high-rate 2,6-DHAQ or 7,8-dihydroxyphenazine-2-sulfonic acid (DHPS), the S-Fe flow cells all achieve a high capacity utilization with low overpotential in three different relay couples (1,4-DHAQ + 2,6-DHAQ, 1,8-DHAQ + 2,6-DHAQ, and 1,4-DHAQ + DHPS), showing its wide universality.

To improve the energy density of the S-Fe RFB, we coupled high-concentration negolyte (1.2 M K_2_S_4_) with a mixed-cation posolyte (0.5 M potassium ferrocyanide + 0.5 M sodium ferrocyanide) to achieve 2.4 M and 1 M effective electron concentration on the negolyte and posolyte, respectively. With the balanced-capacity electrolyte composition, this S-Fe RFB showed a high volumetric capacity of 64 Ah L^−1^_negolyte_ and a low decay rate of 0.00071% per cycle and 0.003% per day over 500 cycles and 2680 h (over 112 days) (Fig. 5c). Since the achieved capacity and energy efficiency are highly related to the catalyst concentration (Supplementary Figs. 56 and 57), the well-maintained discharge capacity and EE (Fig. 5c and Supplementary Fig. 58) verified the high stability of relay catalysts. Furthermore, the capacity contribution from FMN-Na catalysis and 2,6-DHAQ catalysis is almost unchanged, suggesting a high activity of relay catalysts over 112 days (Supplementary Fig. 59). Even at a high current density of 40 mA cm^−2^, the cell still shows a high stability with little capacity decay over 500 cycles with nearly full capacity utilization (Supplementary Figs. 60). We summarized the achieved volumetric capacity and cycling life with the state-of-the-art polysulfide-based redox flow batteries, including S-Fe, S-O_2_, S-I, and S-Mn flow cells (Supplementary Table 2), and the relay-catalysts-assisted cells show a competitive performance. The CRIS membrane enables a high CE (> 99.9%) in long-term operation and eliminate the sulfur passivation on the positive side (Supplementary Figs. 61–63). At the full cell level, the achieved volumetric capacity was 19 Ah L^−1^_posolyte+negolyte_, which is competitive among all reported S-Fe flow batteries (Supplementary Table 2). As a comparison, the S-Fe flow cell with the single FMN-Na catalyst achieved lower capacity and lower stability due to insufficient reaction rate (Supplementary Figs. 64–66).

After demonstrating the effective relay catalysis strategy in the lab-scale 12 cm^2^ flow cell, we scaled up the system to a two-layer, 100 cm^2^ flow cell stack containing 200 mL negolyte and 400 mL posolyte to evaluate its potential in practical engineering applications. One advantage of homogeneous catalysis in large-scale applications is that the simple catalyst dissolution process circumvents the complicated, time-consuming catalyst-electrode preparation/coating process in common heterogeneous catalysis, and it could be easily scaled up by simply dissolving catalysts in more electrolyte within a few seconds. Interestingly, when we increased the cell size to the 100 cm^2^ flow cell stack, two catalytic plateaus were still clearly observed at a high current of 5 A (50 mA cm^−2^), indicating that relay catalysis strategy still functions well in the increased-size cell (Fig. 5d, e). The relay catalyst greatly decreases the overpotential compared with the bare K_2_S_4_ electrolyte (Fig. 5e and Supplementary Fig. 67) and increases the cell capacity by four times to over 4000 mAh. The achieved discharge energy is ~ 8000 mWh. The energy efficiency of the flow cell stack is 73.5% at 30 mA cm^−2^ and 61.3% at 50 mA cm^−2^, which is comparable with the EE in 12 cm^2^ small cells (Supplementary Fig. 68). This 100 cm^2^ flow cell stack shows a stable operation for 200 cycles (over 315 h) at 5 A (Fig. 5f). We further studied the effect of flow rate on electrochemical performance and cell behaviors on the large cell (100 cm^2^ cell). The flow rate has a significant impact on the cell performance. The achieved capacity increased as the flow rate increased from 50 to 200 mL min^−1^ and stayed the same when the flow rate further increased from 200 to 300 mL min^−1^ (Supplementary Fig. 69). These observations suggest that insufficient flow rate could lead to a local concentration gradient and pre-termination of the charging process, while increasing flow rates would lead to a capacity increment but its increment effect would be limited when the mass transfer is sufficient for chemical reactions. Proper design of flow rate could enhance mass transport and eliminate the concentration gradient. A flow rate of 200 mL min^−1^ in our two-layer 100 cm^2^ cell stack yields 1 mL min^−1^ cm^−2^ (flow rate/active area), which is comparable with the all-vanadium flow cell stack and other flow battery systems reported in literature (1–6 mL min^−1^ cm^−2^, Supplementary Table 3). These results all verified that relay catalysis is a facile and highly effective catalytic strategy for practical high-energy, large-scale polysulfide-based flow battery stacks.

We examined the influence of temperature on the relay-catalysis between 10 and 40 °C, in which the relay catalyst promotes the full capacity utilization and decreases overpotential in a wide temperature range (Supplementary Figs. 70–73 and Supplementary Note 10). Considering that the solubility of ferrocyanide posolyte can be increased to 1.5 M at 40 °C, we further improved the energy density of S-Fe RFB by coupling the 1.5 M ferrocyanide (0.75 M K_4_[Fe(CN)_6_] + 0.75 M Na_4_[Fe(CN)_6_]). At 40 °C, this cell achieved a high volumetric capacity of 40.2 Ah L^−1^_posolyte_ based on the posolyte volume and 24.6 Ah L^−1^_posolyte+negolyte_ based on the full cell electrolyte volume (Supplementary Fig. 74). The energy density of this S-Fe RFB full cell reaches 21.65 Wh L^−1^_posolyte+negolyte_, which is more than three times higher than some common polysulfide-based RFBs ( ~ 6–7 Wh L^−1^_posolyte+negolyte_) and comparable with all-vanadium RFBs ( ~ 20 Wh L^−1^_posolyte+negolyte_). Stable cycling of more than 200 cycles was achieved at 60 mA cm^−2^ with a low decay rate of 0.00047% per cycle.

We further verify this relay catalysis approach in polysulfide/iodide redox flow battery (S-I RFB) to show its universality (Supplementary Figs. 75 and 76). The S-I RFB with relay catalysts achieved a high capacity of 32 Ah L^−1^_posolyte+negolyte_ based on full cell level (Supplementary Fig. 76).

### Universality of relay catalysis in redox flow batteries

**Fig. 6:**
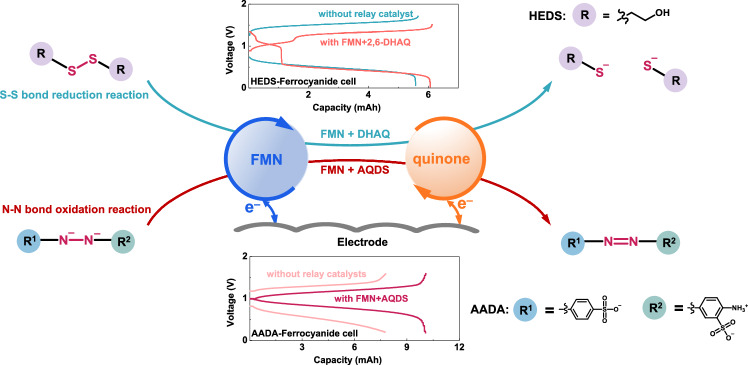
Universal relay catalysis strategy for S–S bond reduction reactions and N–N bond oxidation reactions. The schematic illustration of the relay catalysis strategy on S–S bond reduction reactions and N–N bond oxidation reactions. The FMN-Na and 2,6-DHAQ relay catalysts catalyze S–S bond reduction reactions for organosulfide-based materials like HEDS (light green line). The upper figure shows the voltage profile of HEDS with or without relay catalysts at 10 mA cm^−2^. The FMN-Na and AQDS relay catalysts catalyze N–N bond oxidation reactions of hydrazide-based compounds like reduced AADA (dark red line). The bottom figure shows the voltage profile of AADA with or without relay catalysts at 50 mA cm^−2^.

The relay catalysis strategy can be widely applied to other organic active materials facing the trade-off between catalytic rate and overpotential (low overpotential but low capacity or high capacity but high overpotential). First, we extended this strategy from inorganic polysulfide anions to organosulfides whose structure and properties could be tuned through functional group modification and molecular design (Fig. 6). We selected highly soluble 2-hydroxyethyl disulfide (HEDS)^22,62^ to demonstrate the relay catalysis effect on universal S–S bond cleavage reactions. The reaction mechanism of HEDS is analogous to inorganic polysulfide: during the reduction process, the S–S bond is broken to generate two 2-hydroxyethyl sulfide molecules, while the S–S bond re-forms in the reverse oxidation process. The large size of organosulfides will lead to a lower crossover rate but a high energy barrier to break the S–S bond. Notably, we observed a significant drop in overpotential and improved stability upon introducing the relay catalysts (FMN + 2,6-DHAQ) into the HEDS-ferrocyanide cells (Fig. 6 and Supplementary Fig. 77). The spontaneous chemical reactions between HEDS and reduced FMN and 2,6-DHAQ are verified by the UV–vis spectra, showing that HEDS could oxidize the reduced FMN and 2,6-DHAQ molecules (Supplementary Fig. 78 and Supplementary Note 11).

Beyond sulfur chemistry, we further applied our relay catalysis strategy to nitrogen-based chemistry (Fig. 6), including the oxidation of hydrazide (–N^−^–N^−^–) to azo compound (–N = N–). Modified with amino and sulfonic groups, 4-amino-1,1′-azobenzene-3,4′-disulfonic acid monosodium salt (AADA), an azobenzene derivative, has high solubility and appropriate redox potential as promising negative material candidates but shows insufficient kinetics^21^. The CV tests show AADA has one reduction peak with almost no oxidation peak observed (Supplementary Fig. 79), which corresponds to the sluggish kinetics of the hydrazide oxidation reaction. To catalyze this oxidation reaction, we selected organic molecules with potentials higher than AADA to accelerate it via the fast chemical reaction route. Since electron-withdrawing groups could elevate the redox potential, another anthraquinone derivative, anthraquinone-2,7-disulfonic acid disodium salt (AQDS) with a high potential of −0.29 V vs. SHE (Supplementary Fig. 80a), was adopted as the molecular catalyst to couple with FMN-Na to assist the oxidation of reduced-AADA molecules (Supplementary Fig. 80b). The introduction of relay catalysts (FMN + AQDS) greatly elevated the discharge voltage of AADA-ferrocyanide cell and its discharge capacity at high current density (Fig. 6 and Supplementary Fig. 81). The UV–vis spectra confirmed reduced-AADA could be oxidized by FMN-Na and AQDS (Supplementary Figs. 82 and 83 and Supplementary Note 11). These results indicate that this relay catalysis strategy can be widely applied to various relay-catalyst couples (e.g., FMN + DHAQ, FMN + AQDS, etc.) with matched kinetics and redox potential.

This work greatly broadens the scope of homogeneous catalysis in the redox flow batteries across different materials systems: inorganic polysulfide, organosulfides, and azo compound systems, effectively breaking the trade-off between overpotential and driving force for a single molecular catalyst. We believe this universal relay catalysis strategy can be potentially widely applied to other secondary batteries adopting redox-active catalysts like lithium-oxygen batteries, lithium-sulfur batteries, zinc-manganese batteries, etc. to achieve both high capacity and low overpotential.

## Discussion

We successfully developed a bio-inspired relay catalysis strategy to achieve both high capacity and energy efficiency in aqueous sulfur-based, organosulfide-based, and azo-based redox flow batteries. The operando UV–vis spectra visualized the relay process, revealing the switch of activating catalysts from the low-rate catalyst FMN-Na to the high-rate catalyst 2,6-DHAQ at the limiting concentration. This relay approach was applied to the S-Fe RFB to increase the capacity utilization by 67% at 30 mA cm^−2^ compared with the single catalyst FMN-Na. A high concentration S-Fe RFB containing mixed-cation posolyte demonstrated over 112 days (500 cycles) stability at 64 Ah L^−1^_negolyte_ (19 Ah L^−1^_posolyte+negolyte_) with a low decay rate of 0.00071% per cycle and 0.003% per day. We then scaled up the relay catalysis strategy to a two-layer, 100 cm^2^ battery stack operating at 5 A for over 200 cycles (over 315 h), showing its potential for large-scale long-duration energy storage engineering. We demonstrated its universality in other active materials, including organosulfides and azo-based systems, and verified that quinone molecules are ideal relay catalysts for S–S bond reduction reactions and N–N bond oxidation reactions. This work greatly broadens the scope of homogeneous catalysis in redox flow batteries across different systems for catalytic energy storage applications.

## Methods

### Chemicals and materials

Potassium (poly)sulfide (K_2_S_x_, ≥42% K_2_S basis, *x* is determined to be 2^30^), potassium iodide (KI, ≥ 99%), sulfur (S, reagent grade), 1-methyl-2-pyrrolidinone (NMP, 99.5%) were received from Sigma-Aldrich. Hydrogen peroxide (H_2_O_2_, 30 wt% in H_2_O) was purchased from VWR Chemicals. Riboflavin sodium phosphate (FMN-Na, 93%), potassium chloride (KCl, 99.8%), 1,8-dihydroxyanthraquinone (1,8-DHAQ), HEDS (>90%), and sodium ferrocyanide decahydrate (Na_4_[Fe(CN)_6_]·10H_2_O, 99%) were purchased from Aladdin. 1,4-dihydroxyanthraquinone (1,4-DHAQ, 99%) was received from Dieckmann. Potassium ferrocyanide trihydrate (K_4_[Fe(CN)_6_]·3H_2_O, 99.5%), 7,8-dihydroxyphenazine-2-sulfonic acid (DHPS, 95%), 4-amino-1,1′-azobenzene-3,4′-disulfonic acid monosodium salt (AADA, 90%), and potassium hydroxide (KOH, 95%) were received from Macklin. Anthraquinone-2,7-disulfonic acid disodium salt (AQDS, 96.6%) was received from Leyan. 2,6-dihydroxyanthraquinone (2,6-DHAQ) was received from Bide and Meryer. Nafion membrane (Nafion 117, Dupont) was received from Chemours. Carbon felts (4.6 mm, GFD 4.6EA) were received from SGL Carbon. Ketjen black EC-600JD was received from AkzoNobel. All chemicals were used as received.

### K_2_S_4_ electrolyte preparation

The negolyte was prepared inside the argon-filled glovebox. Potassium hydroxide, sulfur, and K_2_S_2_ were mixed in a molar ratio of 1: 2: 1 and 1: 2.4: 1.2 to form the 1 M K_2_S_4_ and 1.2 M K_2_S_4_ electrolyte with argon-bubbled deionized water. The electrolyte was stirred overnight to form a clear solution. For the cell with FMN-Na and 2,6-DHAQ, the molecular catalysts were dissolved in the K_2_S_4_ electrolyte.

### CRIS membrane preparation

The CRIS membrane was prepared by casting a carbon layer on the treated Nafion membrane. The Nafion membrane was pretreated to K^+^-type before coating. Firstly, the Nafion membranes were treated with 5% H_2_O_2_ for 1 h, and then 5% H_2_SO_4_ for 1 h under 80 °C. 1 M KOH solution was applied to transform the membranes to K^+^-type at 80 °C for 2 h. The treated membranes were rinsed and soaked in DI water overnight before usage. The coating slurry was prepared by mixing KB:PVDF at a ratio of 8:1 in the NMP solution, after which the slurry was sonicated until uniform. The coated membrane was dried at 60 °C for more than 4 h, which was followed by soaking in DI water overnight. The membranes were soaked and stored in DI water before usage.

### Flow cell assembly

The flow cell (Supplementary Fig. 35a) was constructed by using K^+^-type Nafion membrane or CRIS membrane as the separator and carbon felts as the electrodes for both posolyte and negolyte side. The carbon felt was heated at 500 °C to improve its hydrophilicity. The flow cell was composed of stacked layers of carbon felt, carbon plate (with serpentine-type flow channels), and stainless-steel plate on each side (Supplementary Fig. 35b), sandwiching the membrane. The 3 mm PTFE gasket with O-ring was adopted to prevent the electrolyte from leaking. The flow cell was connected to two glass containers with peristaltic pump tubing (Tygon® Chemical) and PTFE tubes. The peristaltic pump (Chuang Rui Precision Pump) was applied to circulate the electrolyte. The geometric area of the electrode is 12 cm^2^ unless otherwise specified. For the S-Fe flow cell with a balanced capacity with a N:P ratio of 1, the posolyte is 40 mL 0.5 M K_4_[Fe(CN)_6_] in 1 M KCl, while the negolyte is 10 mL of 1 M K_2_S_4_ with or without molecular catalysts. For the high concentration mixed cation S-Fe flow cell, 24 mL of mixed 0.5 M K_4_[Fe(CN)_6_] and 0.5 M Na_4_[Fe(CN)_6_] electrolyte was adopted as posolyte, while 10 mL of 1.2 M K_2_S_4_ with 50 mM 2,6-DHAQ and 50 mM FMN-Na was adopted as the negolyte. The high-energy S-Fe flow cell at high-temperature adopted 16 mL of mixed 0.75 M K_4_[Fe(CN)_6_] and 0.75 M Na_4_[Fe(CN)_6_] as posolyte. For the S-I flow cell, the posolyte is 10 mL of 4 M KI or 4.5 M KI solution, and the negolyte composition is the same as the S-Fe test. The flow rate is set as 50 mL min^−1^. All of the flow cells were assembled inside the argon-filled glovebox, and were tested outside the glovebox. The cells were cycled at room temperature under ambient conditions without an environmental chamber, unless otherwise stated.

### Static cell assembly

The static cell assembling process closely resembles that of the flow cell. The carbon plate and carbon felt (${\varnothing}$16 mm) are stacked on each side with a Nafion 117 membrane (3 × 3 cm) as the separator. One (500 ${{{\rm{\mu }}}}$L electrolyte) or two (1000 ${{{\rm{\mu }}}}$L electrolyte) stacked carbon felts were adopted as the electrodes depending on the electrolyte volume. All of the static cells were assembled inside the argon-filled glovebox, and were tested outside the glove box.

### Material characterizations

Scanning electron microscopy (SEM) was conducted by FEI Quanta 400F SEM with an accelerating voltage of 10 kV. The carbon felts were collected from the positive side in S-Fe RFBs after cycling. The samples were washed with deionized water for three times and then dried at room temperature before testing. The Raman spectra were collected by Renishaw inVia Raman microscope.

In situ/operando *UV–vis test:* The SEC2020 UV–visible Spectrophotometer (ALS Co., Ltd) was used to acquire UV–visible spectra. The 1 M KOH solution was adopted as the supporting electrolyte to prepare the sample for the UV–vis test, which was conducted in a thin quartz cell with a 1 mm optical path length. The in situ UV–vis tests were conducted to monitor the chemical reaction rate (Supplementary Fig. 12). The reduced molecular catalysts were collected by reducing them inside a static cell (geometric area of the electrode is 2 cm^2^) under 5 mA cm^−2^ with a cut-off voltage of 1.6 V. After disassembling the static cell, the sample was collected and mixed with K_2_S_4_ electrolyte. The quartz cell was well sealed inside the Ar-filled glovebox before taking the UV–vis test to exclude the possible oxidation from oxygen. For the operando UV–vis spectra, the quartz cell is connected with a flow cell with an active geometric area of 4 cm^2^ (Supplementary Fig. 5). The electrolyte was injected inside the glovebox and carefully sealed before transferring to the outside of the glovebox. For obtaining the operando UV–vis spectra of molecular catalysts themselves (Supplementary Figs. 3 and 4 and Supplementary Figs. 5–7), the negolyte is 1 mM DHAQ in 1 M KOH, and the posolyte is 10 mL 0.25 M K_4_[Fe(CN)_6_] + in 0.05 M K_3_[Fe(CN)_6_] in 1 M KCl. The current density is 3 mA cm^−2^. For the high-concentration molecular catalyst and K_2_S_4_ tests (Figs. 3 and 4), the posolyte composition for the operando UV–vis test is 15 mL 0.25 M K_4_[Fe(CN)_6_] in 1 M KCl (theoretical capacity of 100.5 mAh), while the negolyte composition is 5 mM molecular catalysts, and 250 mM M K_2_S_4_ with or without molecular catalysts.

### Electrochemical measurements

The cyclic voltammetry tests were performed on a VMP3 electrochemical testing unit (Bio-Logic). The three-electrode system consisted of a glassy carbon working electrode, a saturated calomel reference electrode, and a Pt wire counter electrode. The SCE electrode was pre-soaked in a saturated KCl solution and the glassy carbon electrode was polished before testing. The potential ranges of 1,4-DHAQ and 1,8-DHAQ are −0.9 to −0.6 V, whereas that of 2,6-DHAQ is −1.1 V to −0.8 V. The scan ranged from 10 to 50 mV s^−1^. The electrolyte was prepared by dissolving polysulfide, 2,6-DHAQ, 1,8-DHAQ, 1,4-DHAQ, AQDS, FMN-Na, and AADA with 1 M KOH solution. The four-electrode test was conducted by inserting the SCE reference electrode into the electrolyte tank during 4 cm^2^ flow cell operation. The galvanostatic charging tests were performed on a LAND Battery Testing System (LAND, Wuhan Land Electronic Co., Ltd) at room temperature with a current density from 10 to 40 mA cm^−2^. The EIS measurements were performed on the Gamry Interface 1010E testing unit with frequencies ranging from 1000 kHz to 0.1 Hz and six points per decade. The measurements were conducted in potentiostatic mode with an AC amplitude of 10 mV. The S-Fe RFB was terminated with the cut-off voltage of 1.6 V and 0.2 V for charge and discharge process. For the long-term cycling test, the flow cell was pre-cycled with a rate test, in which the cell ran 3 cycles under each current density. The S-Fe battery stack was assembled with two 100 cm^2^ cells in series, in which 200 mL 1 M K_2_S_4_ electrolyte with 50 mM relay catalysts (50 mM FMN-Na + 50 mM 2,6-DHAQ) and 400 mL mixed-cation 1 M ferrocyanide (0.5 M K_4_[Fe(CN)_6_] + 0.5 M Na_4_[Fe(CN)_6_]) electrolyte were adopted as negolyte and posolyte, respectively. Rate tests were conducted prior to the long-cycling test. For the S-I cell, the flow cell was terminated with a capacity limit to avoid the generation of insoluble iodine solid.

### Computational methods

All DFT calculations were performed with the B3LYP/6-311 + G(d,p) level of theory with Grimmes’s DFT-D3 correction method^63^ using the Gaussian 09 software package^64,65^. To mimic the solvent effects, all the highly negative charged structures were fully optimized with an implicit solvation model based on density of water. The true local minima or global minima of interaction configurations were verified by the absence of imaginary vibrational frequency, and the existence of transition state was ensured by the presence of unique imaginary frequency and validated by performing an intrinsic reaction coordinate analysis to confirm that the TS correctly connects the two desired minima. The localized orbital indicator-π and IRI analysis were performed by Multiwfn program and visualized by VMD package^59,66–68^.

## Supplementary information

Supplementary Information

Transparent Peer Review file

## Data Availability

The datasets analysed and generated during the current study are included in the paper and its Supplementary Information. Source data are available at Figshare (10.6084/m9.figshare.31999533). The atomic coordinates of the optimised electronic configuration of the DFT calculations are included within the Source Data file in the same Figshare repository.
