# Supplementary material for: Bio-inspired relay catalysis for aqueous redox flow batteries
Source: Nat Commun. 2026 May 27;17:6882. doi: 10.1038/s41467-026-73670-4 (PMC13389366; doi:10.1038/s41467-026-73670-4)
Supplement: Supplementary file 1 — Supplementary Information [file 41467_2026_73670_MOESM1_ESM.pdf]

Supplementary Materials

Bio-inspired relay catalysis for aqueous redox flow batteries

Jiafeng Lei[1,†], *Yaqin Zhang*[2,†], *Weixing Wu*[3], *Ying Wang*[3], *Jun Fan*[2]*, *and Yi-Chun Lu*[1]*

[1]Electrochemical Energy and Interfaces Laboratory, Department of Mechanical and Automation Engineering, The Chinese University of Hong Kong, Shatin, N. T. 999077, Hong Kong SAR (China)

[2]Department of Materials Science and Engineering, City University of Hong Kong, Hong Kong SAR (China)

[3]Department of Chemistry, The Chinese University of Hong Kong, Shatin, N. T. 999077, Hong Kong SAR (China)

†These authors contributed equally to this work

Corresponding authors:

Correspondence to Jun Fan and Yi-Chun Lu

*E-mail: junfan@cityu.edu.hk; yichunlu@mae.cuhk.edu.hk

Supplementary Note 1 | Thermodynamic analysis of homogeneous chemical reaction of molecular catalyst and polysulfide species

The charging process of polysulfide in the presence of molecular catalyst involves the electrochemical and spontaneous chemical reactions. The cell voltage is mainly dominated by molecular catalyst. The reaction mechanism of polysulfide (PS) and molecular catalyst (MC) and their Nernst equations (Eqn (S1-S4)) are listed below:

$$2\,S_2^{2-} \rightleftharpoons S_4^{2-} + 2\,e^- \tag{S1}$$

$$E_{ps} = E_{ps}^{\circ} + \frac{RT}{nF}\ln\frac{a_{S4^{2-}}}{a_{S2^{2-}}^{2}} \tag{S2}$$

$$MC^{2-} \rightleftharpoons \mathrm{MC} + 2\,e^- \tag{S3}$$

$$E_{MC} = E_{MC}^{\circ} + \frac{RT}{nF}\ln\frac{a_{MC}}{a_{MC^{2-}}} \tag{S4}$$

where E and E^0 represent redox potential and standard potential, R is the universal gas constant, F is Faraday's constant, and a is the activity of active materials. n is the number of electrons involved, which is 2 for polysulfide and molecular catalysts for simplicity. The driving force of the chemical reaction between molecular catalyst and polysulfide (Eqn (S5-S6)) is affected by the potential difference and activities of both polysulfide and molecular catalysts:

$$\Delta E = E_{PS} - E_{MC} = \left(E_{ps}^{\circ} + \frac{RT}{2F}\ln\frac{a_{S4^{2-}}}{a_{S2^{2-}}^{2}}\right) - \left(E_{MC}^{\circ} + \frac{RT}{2F}\ln\frac{a_{MC}}{a_{MC^{2-}}}\right)$$

$$= (E_{ps}^{\circ} - E_{MC}^{\circ}) + \left(\frac{RT}{2F}\ln\frac{a_{S4^{2-}}}{a_{S2^{2-}}^{2}} - \frac{RT}{2F}\ln\frac{a_{MC}}{a_{MC^{2-}}}\right)$$

$$= \Delta E^{\circ} + \frac{RT}{2F}\ln\frac{(a_{S4^{2-}}) * (a_{MC^{2-}})}{(a_{S2^{2-}}^{2}) * a_{MC}} \tag{S5}$$

$$\Delta \mathrm{G} = -\,\mathrm{n}\ \mathrm{F}\ \Delta \mathrm{E} \tag{S6}$$

According to the above equation, the driving force of this chemical reaction is highly dependent on the redox potential of molecular catalyst, that is, a more negative potential of molecular catalyst brings a larger ΔE° but a higher energy loss to drive this reaction, highlighting the trade-off of single molecular catalyst.

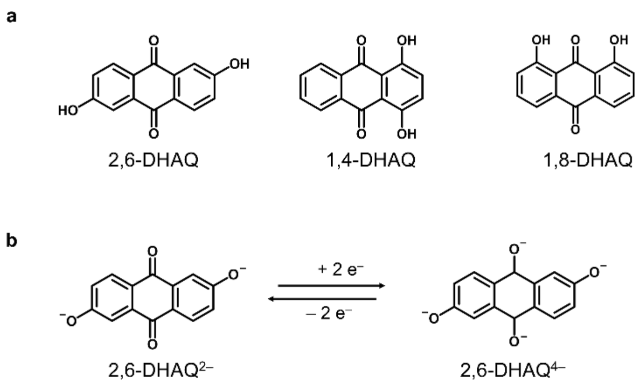

Supplementary Figure 1 | (a) The molecular structure of 2,6-DHAQ, 1,4-DHAQ, and 1,8-DHAQ. (b) Representative reversible two-electron redox reaction of deprotonated $DHAQ^{2-}$ and $DHAQ^{4-}$ in strong alkaline media with 2,6-DHAQ as the example.

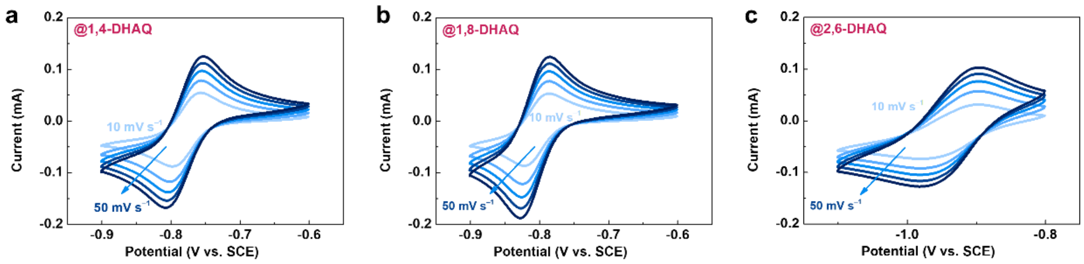

Supplementary Figure 2 | The CV plots of 10 mM (a) 1,4-DHAQ; (b) 1,8-DHAQ; and (c) 2,6-DHAQ.

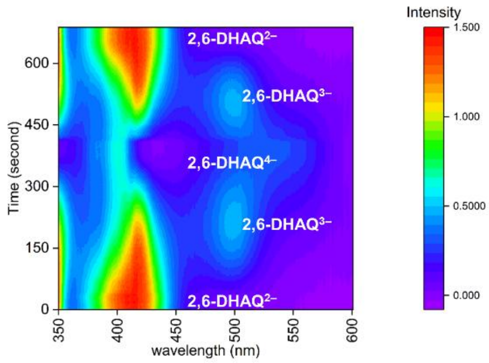

Supplementary Figure 3 | The operando UV-vis spectra of 1 mM 2,6-DHAQ during reduction and oxidation.

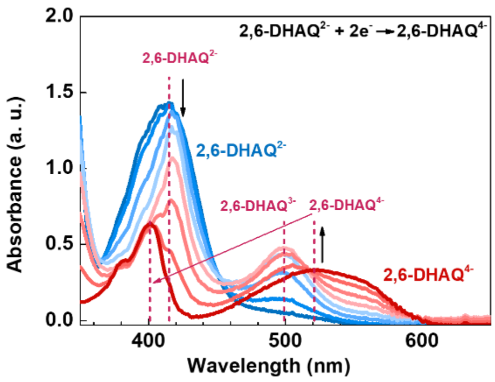

Supplementary Figure 4 | The operando UV-vis spectra of 1 mM 2,6-DHAQ during reduction.

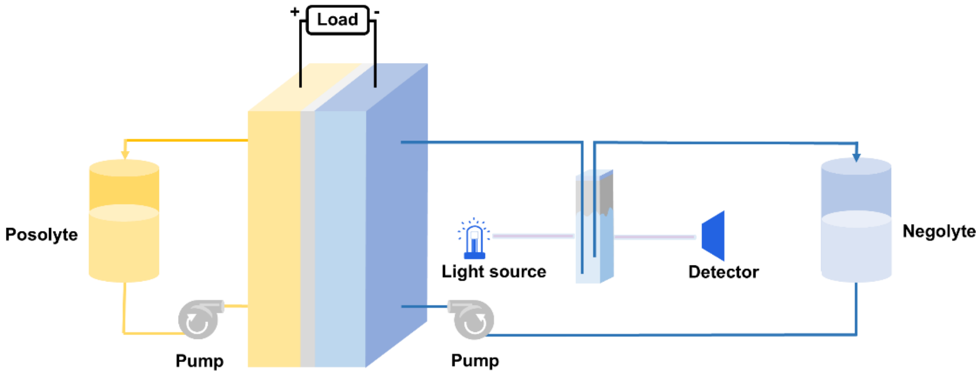

Supplementary Figure 5 | The schematic illustration of the operando UV-vis test.

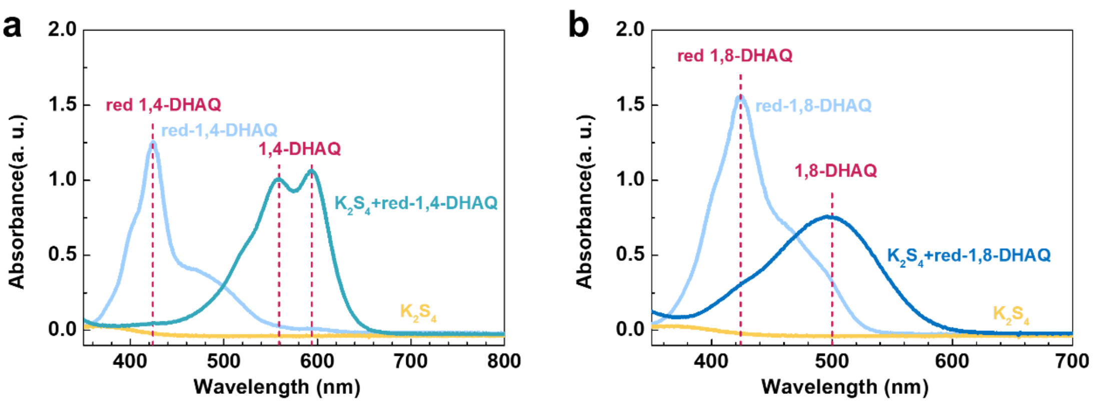

Supplementary Figure 6 | (a) The UV-vis spectra of 1 mM K_2S_4, 1 mM reduced 1,4-DHAQ and a mixed solution of K_2S_4 + reduced 1,4-DHAQ. (b) The UV-vis spectra of 1 mM K_2S_4, 1 mM reduced 1,8-DHAQ and a mixed solution of K_2S_4 + reduced 1,8-DHAQ.

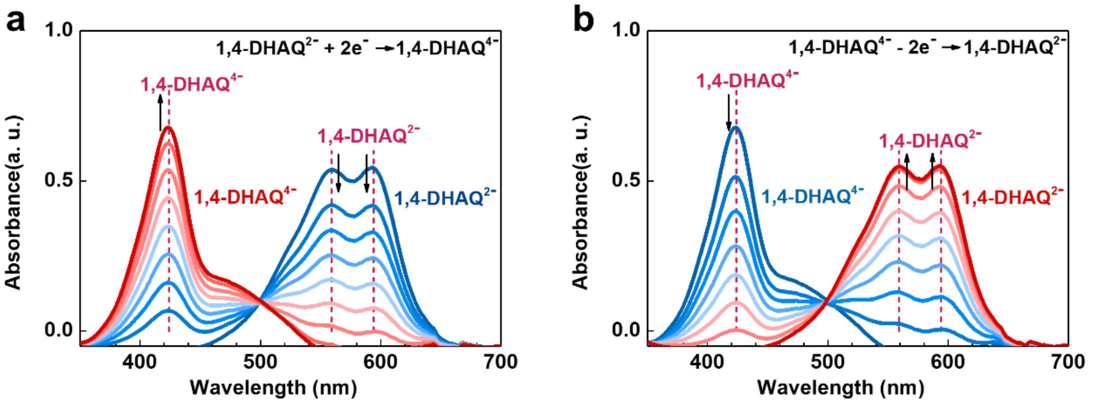

Supplementary Figure 7 | The operando UV-vis spectra of the 1,4-DHAQ during (a) reduction and (b) oxidation.

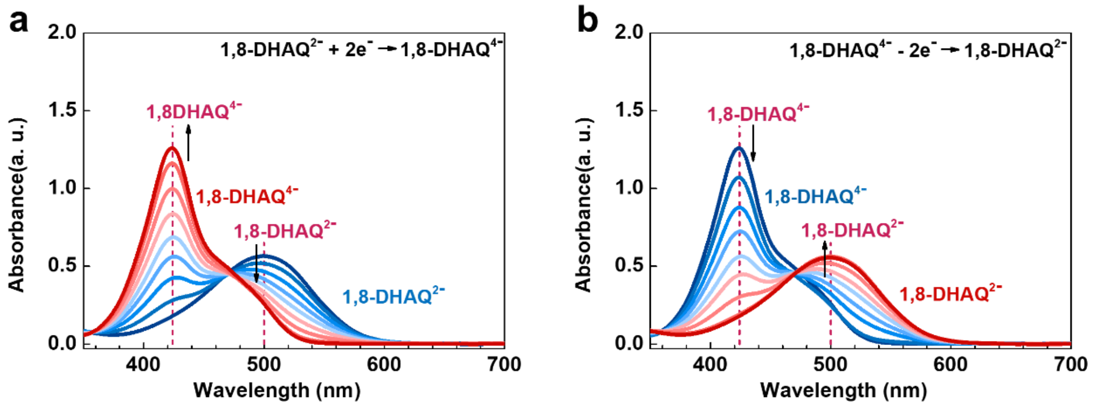

Supplementary Figure 8 | The operando UV-vis spectra of the 1,8-DHAQ during (a) reduction and (b) oxidation.

Supplementary Note 2 | Operando UV-vis spectra analysis of 2,6-DHAQ, 1,4-DHAQ and 1,8-DHAQ with polysulfide.

The operando UV-vis spectra support the chemical reduction of polysulfide by 2,6-DHAQ. During the 2,6-DHAQ oxidation process, the absorbance at 520 nm gradually decreases, while the absorbance at 401 nm shifted to 412 nm (Fig. 2c). This trend is consistent with the chemical oxidation process (Fig. 2b). Interestingly, we further observed the signal from intermediate 2,6-$DHAQ^{3-}$ whose absorbance was centered at 499 nm (Supplementary Figs. 3-4). These changes fully reversed back during the reduction process (Supplementary Fig. 4).

The chemical reactions between the reduced soluble catalyst and K_2S_4 are observed in 1,4-DHAQ (Supplementary Fig. 6a). When mixing the reduced catalyst 1,4-$DHAQ^{2-}$ with K_2S_4, the absorbance of 1,4-$DHAQ^{4-}$ (464 nm) decreases, but the absorbance of 1,4-$DHAQ^{2-}$ (594 nm and 554 nm) increases. The operando UV-vis spectra reveal that the electrochemical reduction of 1,4-DHAQ makes the absorbance at 594 nm and 554 nm decrease accompanied by the increase at 464 nm (Supplementary Fig. 7a). The reversible changes in the absorbance are found in the electrochemical oxidation process (Supplementary Fig. 7b).

The analogous phenomenon of the chemical reaction between the reduced 1,8-$DHAQ^{2-}$ with K_2S_4 was detected. The redshift of the peak from 424nm to 495 nm indicates the chemical oxidation of 1,8-$DHAQ^{4-}$ (Supplementary Fig. 6b), which is supported by our in situ UV-vis spectra. The absorbance of 424 nm and 495 nm is ascribed to 1,8-$DHAQ^{4-}$ and 1,8-$DHAQ^{2-}$ (Supplementary Fig. 8). The results indicate that the reduced $DHAQ^{4-}$ could transfer electrons to K_2S_4 and be oxidized back to $DHAQ^{2-}$, continuing to get electrons and chemically reducing K_2S_4.

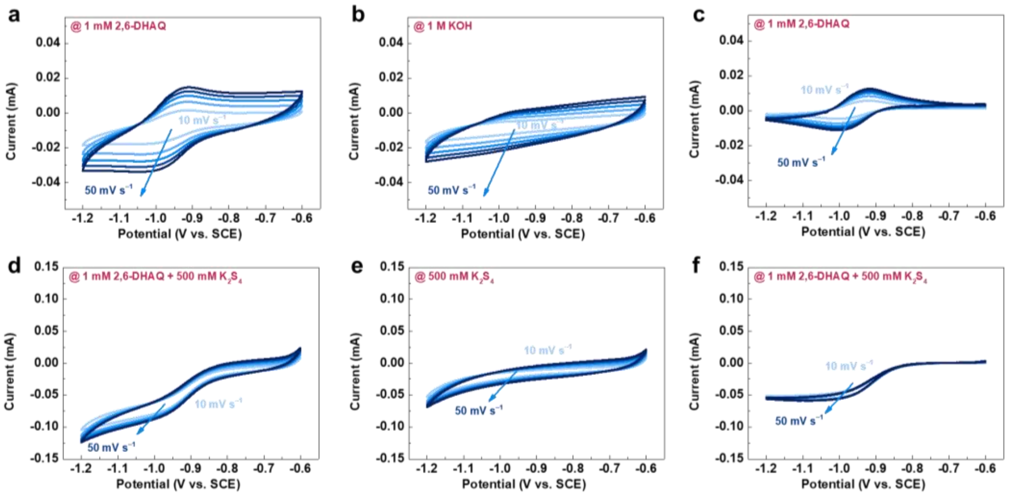

Supplementary Figure 9 | (a) The CV plot of 1 mM 2,6-DHAQ. (b) The CV plot of 1 M KOH. (c) The modified CV plot of 1 mM 2,6-DHAQ after removing the background current from (b). The peak current after background correction was selected as i_p. (d) The CV plot of the mixed solution of 1 mM 2,6-DHAQ + 500 mM K_2S_4. (e) The CV plot of 500 mM K_2S_4. (f) The modified CV plot of the mixed solution of 1 mM 2,6-DHAQ + 500 mM K_2S_4 after removing the background current from (e). The plateau current after background correction was selected as i_{cat}.

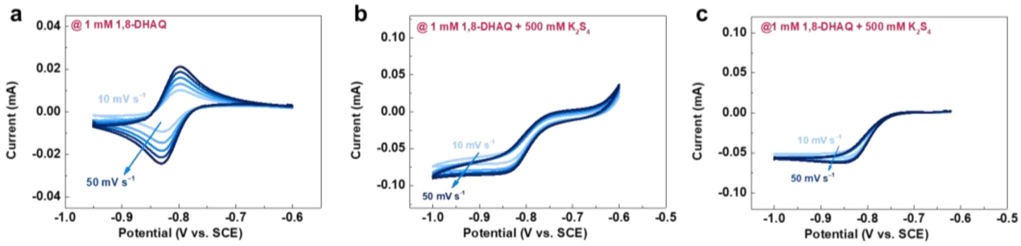

Supplementary Figure 10 | (a) The CV plot of 1 mM 1,8-DHAQ after removing the background current. The peak current after background correction was selected as i_p. (b) The CV plot of the mixed solution of 1 mM 1,8-DHAQ + 500 mM K_2S_4. (c) The modified CV plot of the mixed solution of 1 mM 1,8-DHAQ + 500 mM K_2S_4 after removing the background current. The plateau current after background correction was selected as i_{cat} for calculating the k_{obs} of 1,8-DHAQ.

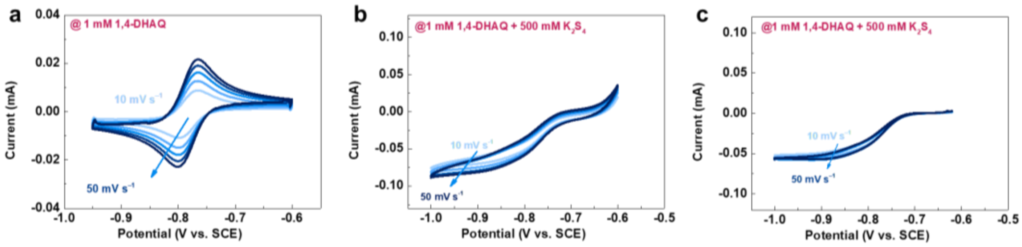

Supplementary Figure 11 | (a) The CV plot of 1 mM 1,4-DHAQ after removing the background current. The peak current after background correction was selected as i_p. (b) The CV plot of the mixed solution of 1 mM 1,4-DHAQ + 500 mM K_2S_4. (c) The modified CV plot of the mixed solution of 1 mM 1,4-DHAQ + 500 mM K_2S_4 after removing the background current. The plateau current after background correction was selected as i_{cat} for calculating the k_{obs} of 1,4-DHAQ.

Supplementary Note 3 | The kinetics analysis of the homogeneous electron transfer rate of 2,6-DHAQ.

We quantified the homogeneous electron transfer rate between DHAQ and polysulfide species. The cyclic voltammogram of 2,6-DHAQ in the presence of high-concentration K_2S_4 shows the disappearance of the oxidation peak, while the reduction current increases significantly, verifying the homogeneous catalysis effect (Supplementary Fig. 9). The observed catalytic plateau reduction current is almost unchanged with scan rate, indicating the chemical reaction rate is the rate-determining step (Supplementary Fig. 9d). At high polysulfide concentration over DHAQ concentration, the catalytic reaction rate could be calculated by Eqn (S7)[1-3]:

$$\frac{i_{cat}}{i_p} = \frac{n}{0.4463\,n'}\sqrt{\frac{RTk_{obs}}{n'\mathrm{F}v}} \tag{S7}$$

where i_{cat} is the plateau catalytic reduction current, i_p is the peak current of DHAQ itself measured without the presence of polysulfide, R is the gas constant, T is the temperature, F is Faraday's constant, v is the scan rate, n' is the electrons transferred to DHAQ in the absence of polysulfide, while n is the electrons transferred from DHAQ to polysulfide, and k_{obs} is the observed rate constant which is also called turnover frequency. n and n' are selected as 2 for simplicity. The background capacitive current is removed for accuracy. The i_p and i_{cat} were picked as 0.0111 mA and 0.0584 mA for 1 mM 2,6-DHAQ electrolyte and 1 mM 2,6-DHAQ + 500 mM K_2S_4 electrolyte, respectively, under a scan rate of 50 mV s^{-1} after background correction (Supplementary Fig. 9), yielding a k_{obs} of 21.4 s^{-1}. However, the k_{obs} of 1,8-DHAQ and 1,4-DHAQ are 5.1 s^{-1} and 4.8 s^{-1} at 500 mM K_2S_4, respectively, showing that 2,6-DHAQ has the highest k_{obs} value among three DHAQ derivatives (Fig. 2c, Supplementary Figs. 9-11). Fig. 2e-f shows a linear increase in k_{obs} of 2,6-DHAQ with respect to the polysulfide concentration, suggesting the catalytic rate has a first-order dependence on the polysulfide concentration. These results all verified 2,6-DHAQ has a high catalytic reaction rate for polysulfide reduction reaction via homogeneous catalysis.

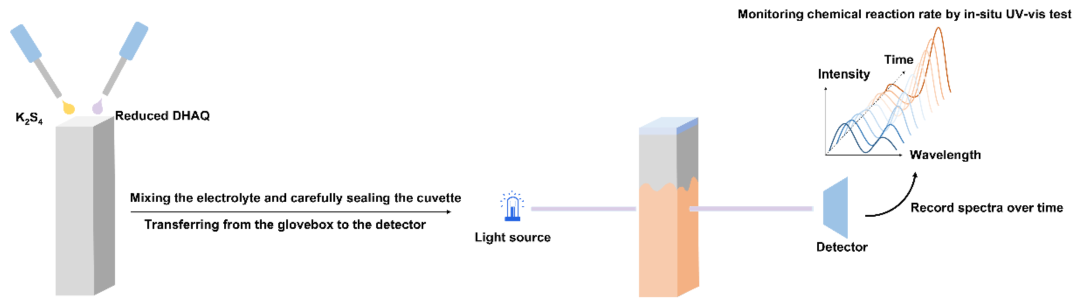

Supplementary Figure 12 | The schematic illustration of the in situ UV-vis test to detect the chemical reaction rate.

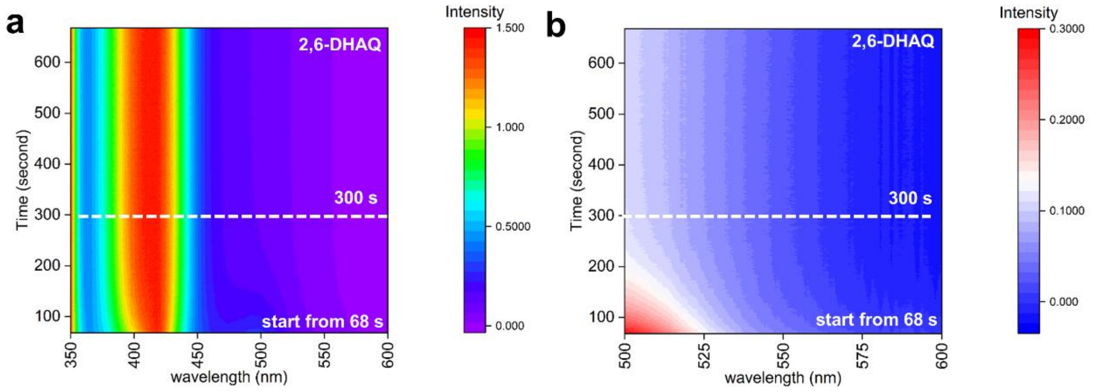

Supplementary Figure 13 | (a) The in situ UV-vis spectra of the mixed solution of 1 mM 2,6-DHAQ + 1 mM K_2S_4. (b) The enlarged region in 500 nm – 600 nm.

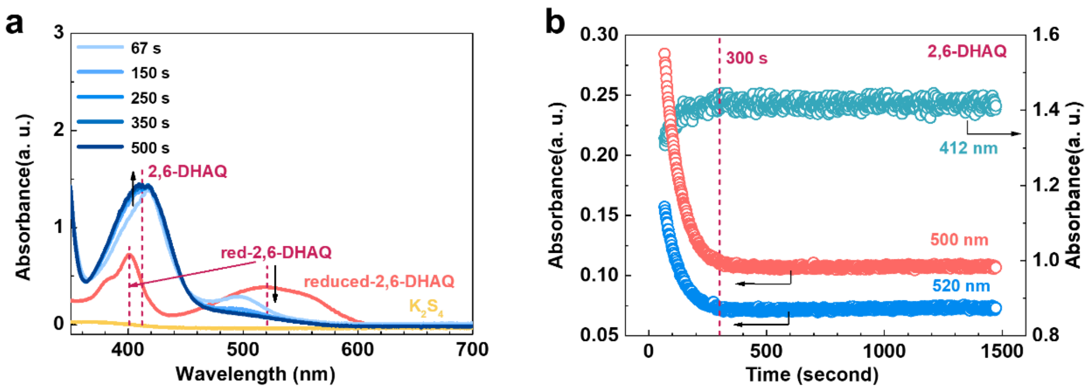

Supplementary Figure 14 | (a) The in situ UV-vis spectra of the mixed solution of K_2S_4 + red-2,6-DHAQ. (b) The absorbance of the mixed solution of 1 mM 2,6-DHAQ + 1 mM K_2S_4 at 412 nm, 500 nm and 520 nm.

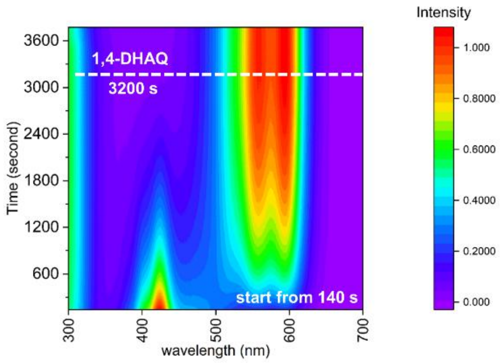

Supplementary Figure 15 |The in situ UV-vis spectra of the mixed solution of 1 mM 1,4-DHAQ + 1 mM K_2S_4.

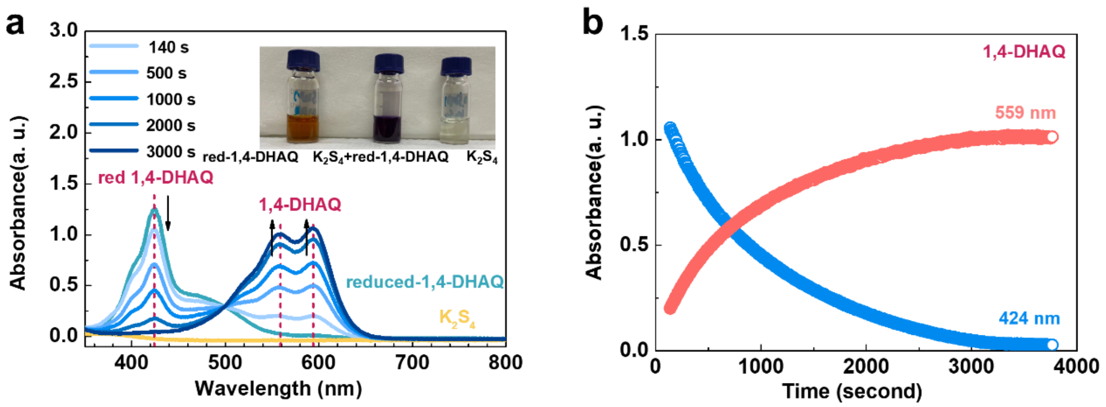

Supplementary Figure 16 | (a) The in situ UV-vis spectra of the mixed solution of K_2S_4 + red-1,4-DHAQ. The inset shows the optical images of 1 mM K_2S_4, 1 mM reduced 1,4-DHAQ (red-1,4-DHAQ) and a mixed solution of K_2S_4 + red-1,4-DHAQ. (b) The absorbance of the mixed solution of 1 mM 1,4-DHAQ + 1 mM K_2S_4 at 424 nm and 559 nm.

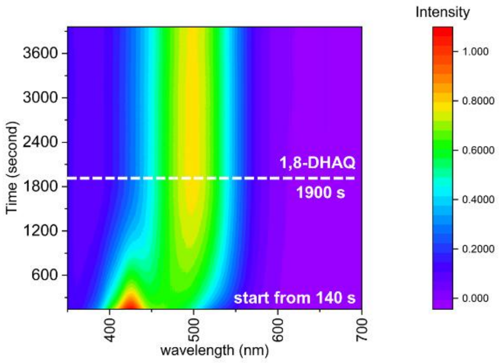

Supplementary Figure 17 |The operando UV-vis spectra of the mixed solution of 1 mM 1,8-DHAQ + 1 mM K_2S_4.

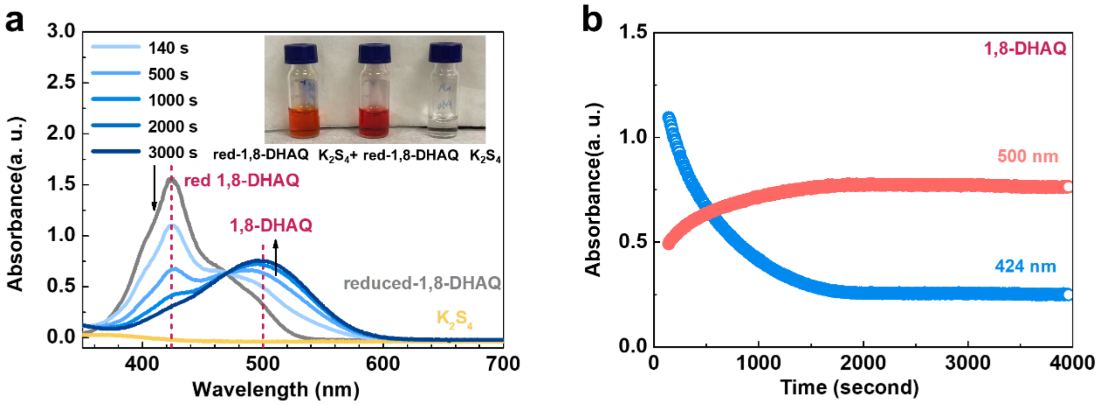

Supplementary Figure 18 | (a) The in situ UV-vis spectra of the mixed solution of K_2S_4 + red-1,8-DHAQ. The inset shows the optical images of 1 mM K_2S_4, 1 mM reduced 1,8-DHAQ (red-1,8-DHAQ) and a mixed solution of K_2S_4 + red-1,8-DHAQ. (b) The absorbance of the mixed solution of 1 mM 1,8-DHAQ + 1 mM K_2S_4 at 424 nm and 500 nm.

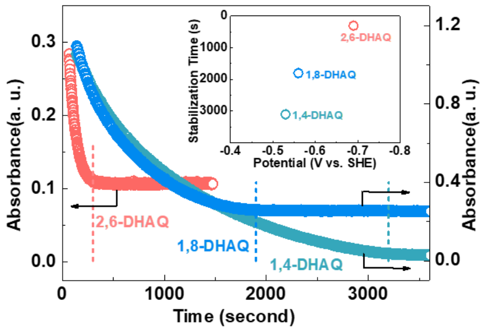

Supplementary Figure 19 | The UV-vis absorbance of 2,6-DHAQ (500 nm), 1,8-DHAQ (424 nm) and 1,4-DHAQ (424 nm). The inset shows the relationship between potential and reaction time.

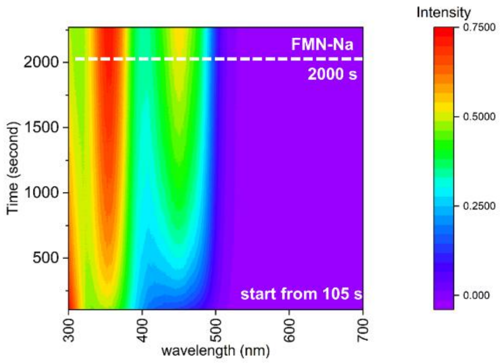

Supplementary Figure 20 |The in situ UV-vis spectra of the mixed solution of 1 mM FMN-Na + 1 mM K_2S_4.

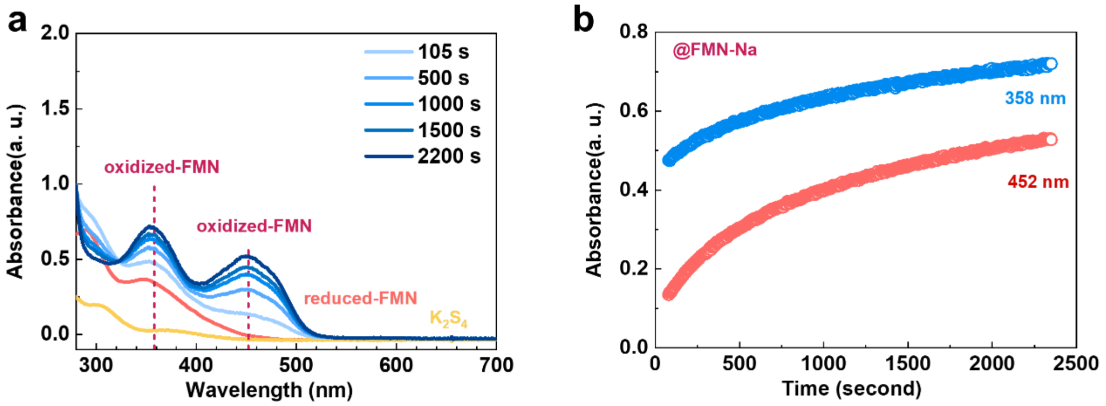

Supplementary Figure 21 | (a) The operando UV-vis spectra of the mixed solution of 1 mM FMN-Na + 1 mM K_2S_4. (b) The absorbance of mixed of the mixed solution of 1 mM FMN-Na + 1 mM K_2S_4 at 358 nm and 452 nm.

Supplementary Note 4 | Comparison of homogeneous electron transfer rates by in situ UV–vis analysis.

The in situ UV-vis test was conducted to study the chemical reaction rate (Supplementary Figs. 12). To fully exclude the possible oxidation from oxygen, we prepared and sealed the sample inside an argon-filled glovebox before taking the UV-vis test. After 67 seconds (the time required for sealing and transferring the sample from the glovebox) from mixing reduced 2,6-DHAQ with K_2S_4, the absorbance peak at 400 nm (2,6-DHAQ^{4-}) is red-shifted to 412 nm (2,6-DHAQ^{2-}) and continuously increases, but the absorbance at 500 nm (2,6-DHAQ^{3-}) and 520 nm (2,6-DHAQ^{4-}) quickly decrease and remain stable after 300 seconds, suggesting 2,6-DHAQ^{4-} is fully oxidized to 2,6-DHAQ^{2-} within this short time period (Supplementary Figs. 13-14). The absorbance of 1,4-DHAQ and 1,8-DHAQ was found to reach the steady state after 3200 seconds and 1900 seconds (Supplementary Figs. 15-18), reflecting the catalytic rate difference of different molecular catalysts, that is 2,6-DHAQ has the highest catalytic rate, while 1,4-DHAQ has the lowest rate (Supplementary Figs. 19). However, the absorbance of the mixed solution of reduced FMN-Na with K_2S_4 showed a gradual increase in absorbance at 358 nm and 452 nm (FMN^{3-}) even after 2,000 seconds (Supplementary Figs. 20-21). The in situ UV-vis spectra analysis suggests 2,6-DHAQ has a high catalytic rate and is well-suited for relay catalysis.

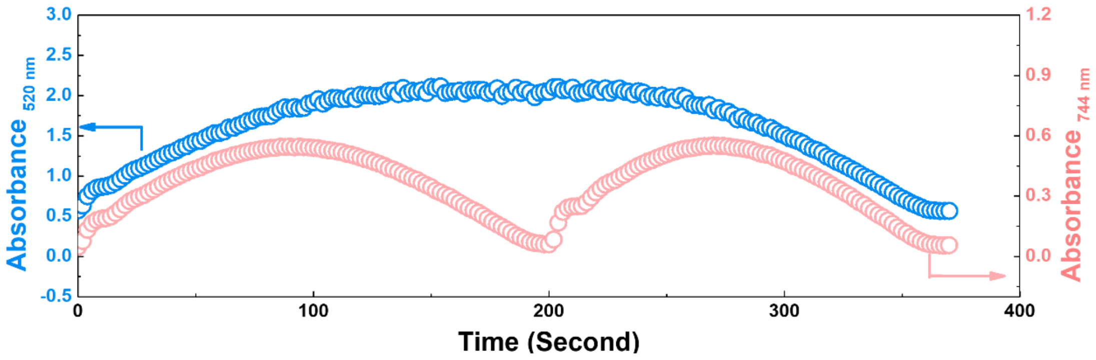

Supplementary Figure 22 | The absorbance of 5 mM 2,6-DHAQ at 520 nm (blue line) and 744 nm (pink line).

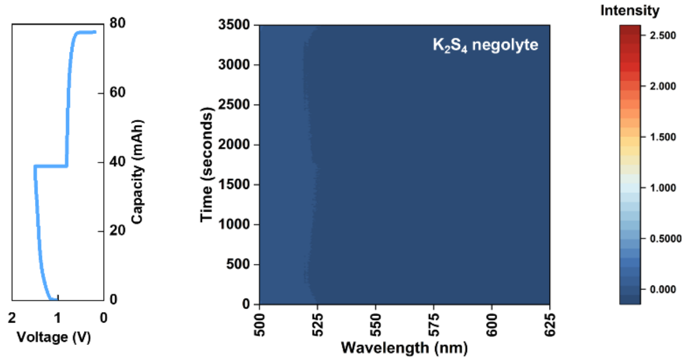

Supplementary Figure 23 | The voltage profiles and operando UV-vis spectra of 250 mM K_2S_4 in a S-Fe flow cell with a cut-off voltage of 1.5 V (10 mL of 250 mM K_2S_4–1 M KOH |Nafion 117 membrane| 15 mL of 0.25 M $K_4[Fe(CN)_6]$–1 M KCl).

Supplementary Note 5 | The operando UV-vis spectra of 2,6-DHAQ

We selected 250 mM K_2S_4 and 5 mM 2,6-DHAQ to ensure proximity to the practical condition while allowing for the detection and differentiation of different states of DHAQ. During charge, the absorbance at 520 nm keeps increasing, corresponding to the progressive reduction from $DHAQ^{2-}$ to $DHAQ^{4-}$ (Fig. 2h). Interestingly, there is an initial increase followed by a complete disappearance of absorbance at 744 nm, which strongly suggests the presence of intermediate $DHAQ^{3-}$ (Fig. 2i). Although a similar trend of $DHAQ^{3-}$ at 500 nm was also observed, the overlapping absorbance from $DHAQ^{4-}$ may interfere our analysis. During discharge, this change is fully reversible, corresponding to the DHAQ oxidation process (Fig. 2h-i). The UV-vis spectra reveal that 2,6-DHAQ undergoes two separate electron transfer processes (Supplementary Fig. 22). However, no noticeable signals were observed for polysulfide species in this region during one cycle (Supplementary Fig. 23).

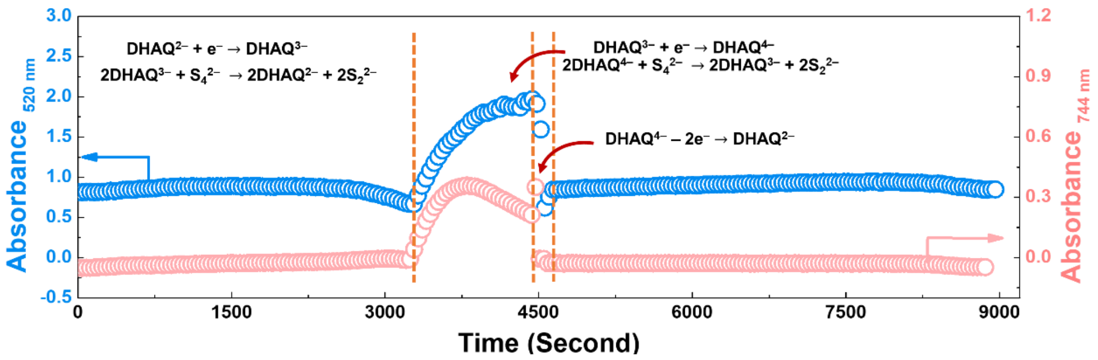

Supplementary Figure 24 | (a) The absorbance of 250 mM K_2S_4 with 5 mM 2,6-DHAQ at 520 nm (blue line) and 744 nm (pink line).

Supplementary Note 6 | Theoretical calculations on the 2,6-DHAQ molecular catalyst

Fig. 3a shows the HOMO (the highest occupied molecular orbital) and LUMO (the lowest unoccupied molecular orbital) energy levels of 2,6-DHAQ, reduced 2,6-DHAQ, and K_2S_4. With the reduction of 2,6-DHAQ, both the HOMO and LUMO levels are elevated, accompanied by the narrowed energy gap between HOMO and LUMO from 0.1306 to 0.0867 a.u.. The narrowed energy gap indicates better electronic conductivity, and the higher HOMO level means easier electron donation. It clearly shows that the HOMO of 2,6-DHAQ^{4-} is the closest to the LUMO of K_2S_4, suggesting an energy favorable orbital interaction. The HOMO of 2,6-DHAQ^{4-} mainly spread around the aromatic rings and the active oxygen atoms, denoting that these rings favor the electron reserving and migrating. The LUMO of K_2S_4 is located at the sulfur atoms, demonstrating that these sulfur atoms can receive electrons to break the disulfide bond. In detail, the HOMO of 2,6-DHAQ^{4-} shows a π-character, thus the conjugation effects of π-electrons are presented by the localized orbital locator-π map (Fig. 3b). The increased π orbital dispersion across the whole molecule (2,6-DHAQ^{4-}) demonstrates the increased degree of molecular conjugation, leading to the narrowed HOMO-LUMO gap.

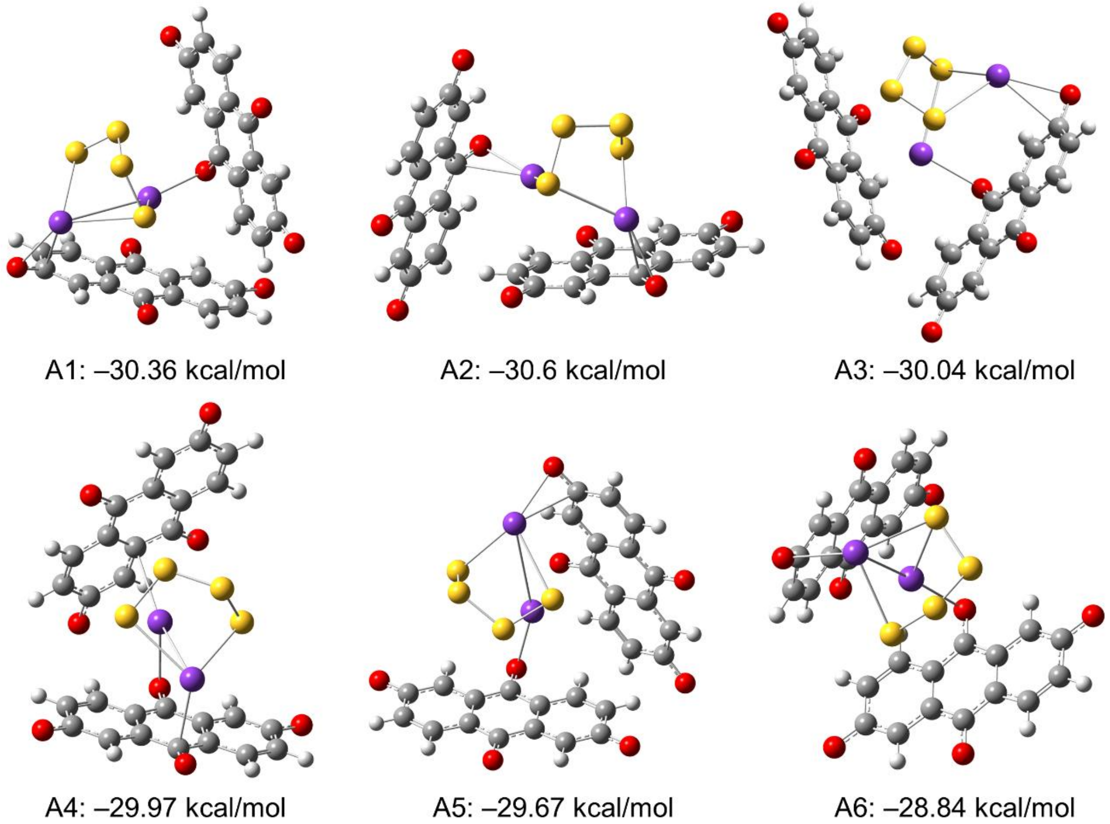

Supplementary Figure 25 | Interacting configurations (K_2S_4-2DHAQ^{4-}) with V_1-type (θ< 90°, L< 10 Å), where the angle between norm of two DHAQ planes is less than 90°, and the distance between the geometric center of the DHAQ molecules is less than 8.5 Å. Atoms are color-coded as follows unless otherwise noted: C (grey), H (white), O (red), S (yellow), and K (purple).

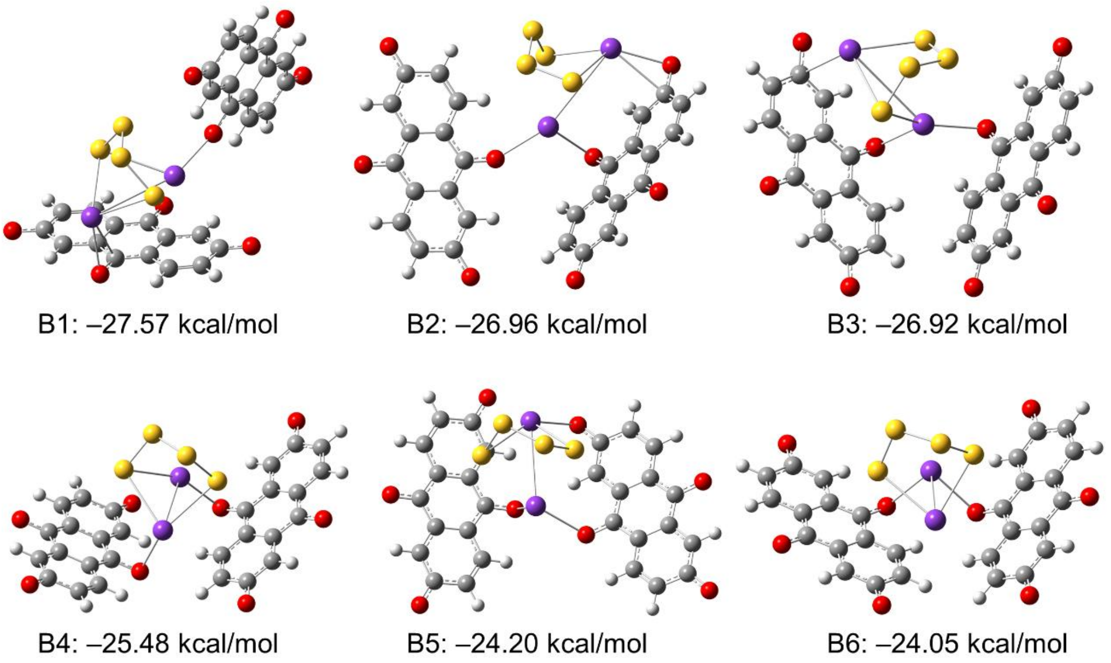

Supplementary Figure 26 | Interacting configurations (K_2S_4-2DHAQ^{4-}) with V_2-type (θ> 90°, L< 10 Å), where the angle between norm of two DHAQ planes is greater than 90°, and the distance between the geometric center of the DHAQ molecules is less than 8.5 Å.

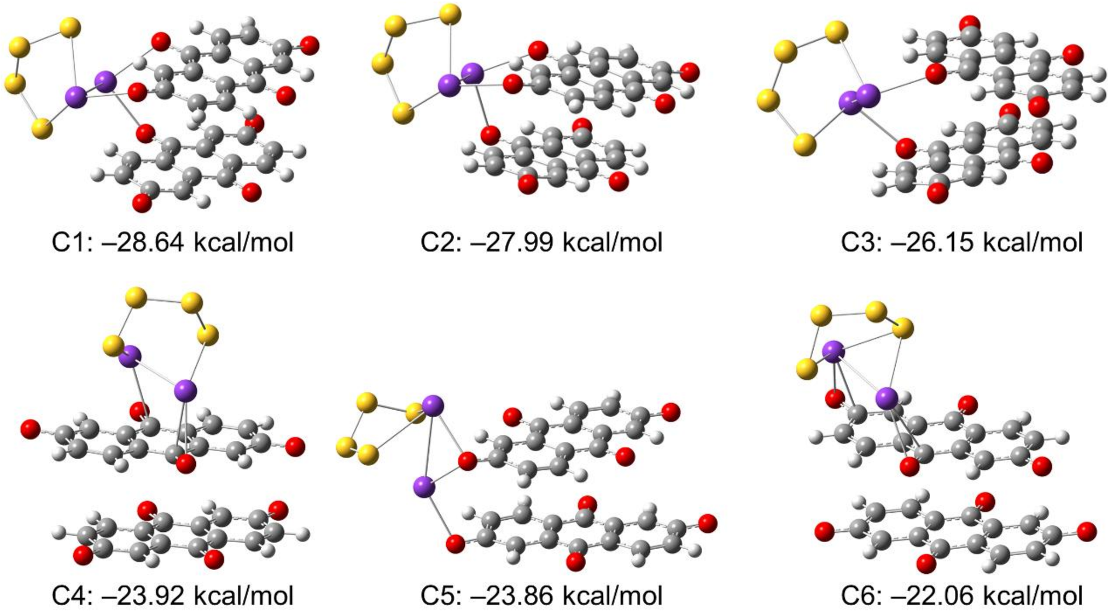

Supplementary Figure 27 | Interacting configurations (K_2S_4-2DHAQ^{4-}) with π-type (θ<5°, π-π stacking between DHAQ planes), where the angle between norm of two DHAQ planes is less than 5°, and the distance between the geometric center of the DHAQ molecules is less than 5 Å.

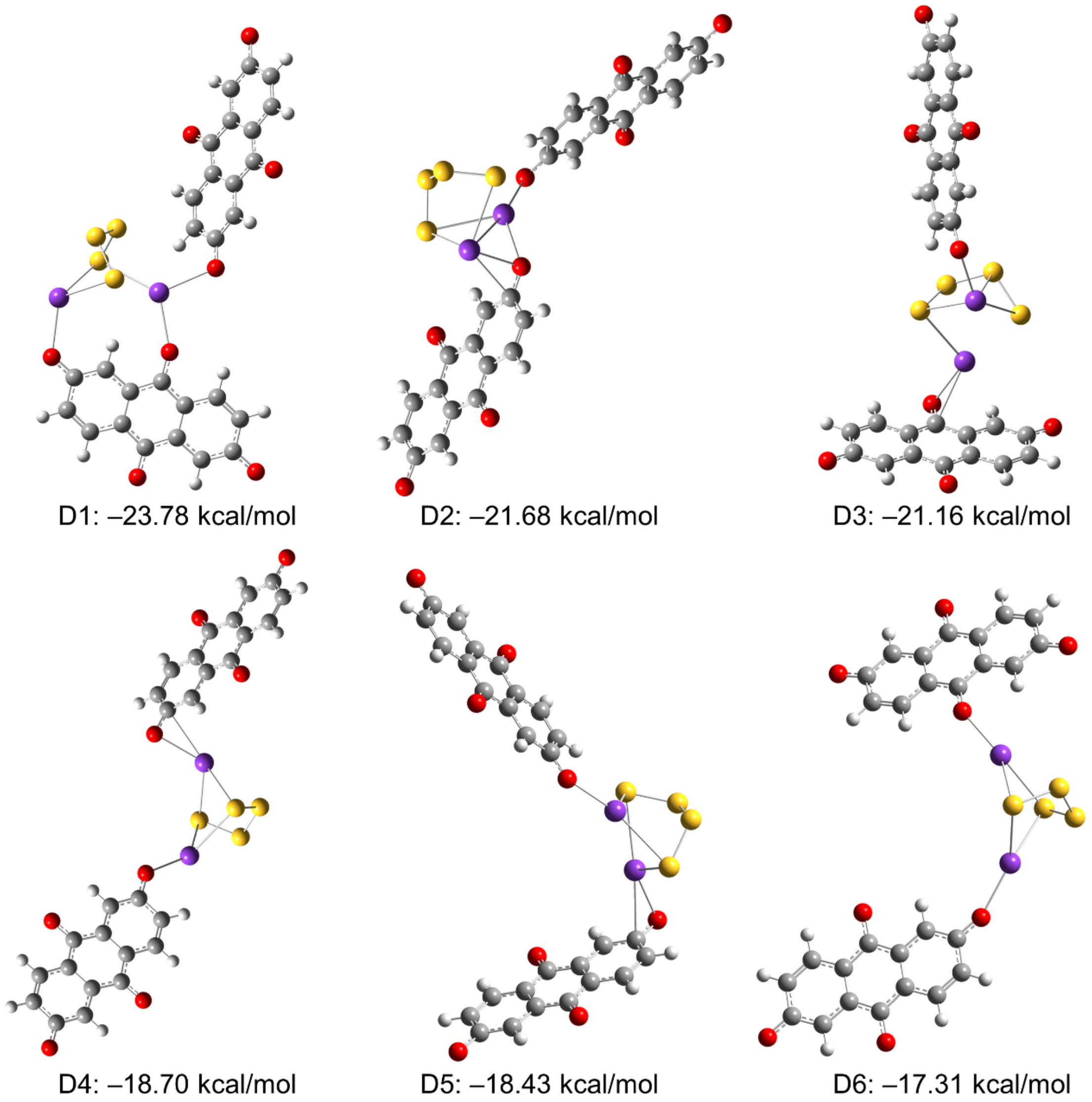

Supplementary Figure 28 | Interacting configurations (K_2S_4-2DHAQ^{4-}) with chain-type (L>10 Å, chain-like connection), where distance between the geometric center of the DHAQ molecules is greater than 10 Å and interaction between rings is weak.

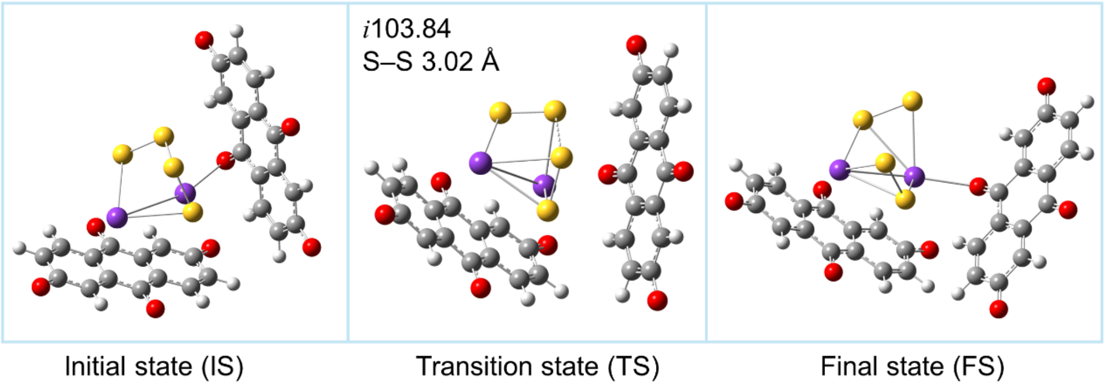

Supplementary Figure 29 | Optimized structures of the intermediate and transition state (TS-A1) catalyzed by two $DHAQ^{4-}$.

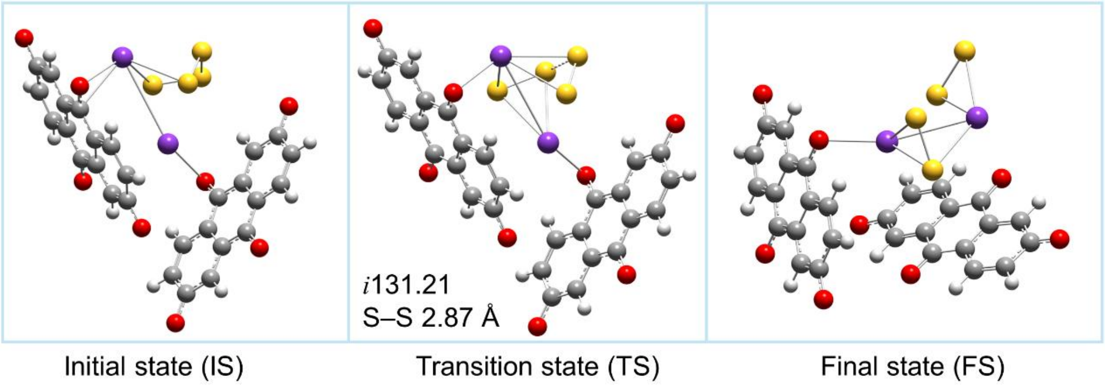

Supplementary Figure 30 | Optimized structures of the intermediate and transition state (TS-B1) catalyzed by two $DHAQ^{4-}$.

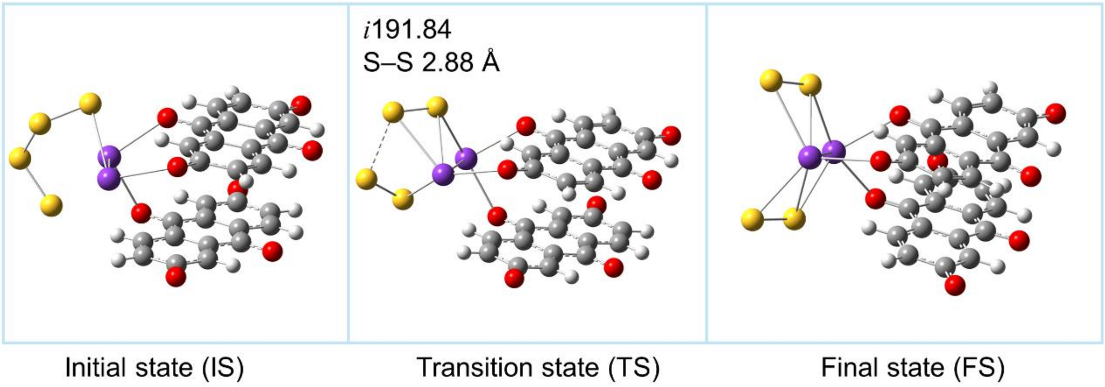

Supplementary Figure 31 | Optimized structures of the intermediate and transition state (TS-C1) catalyzed by two $DHAQ^{4-}$.

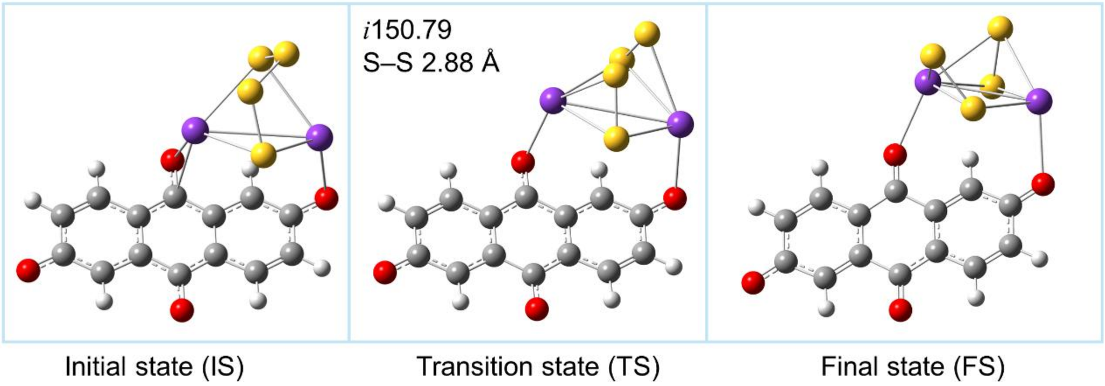

Supplementary Figure 32 | Optimized structures of the intermediate and transition state (TS-single) catalyzed by single $DHAQ^{4-}$.

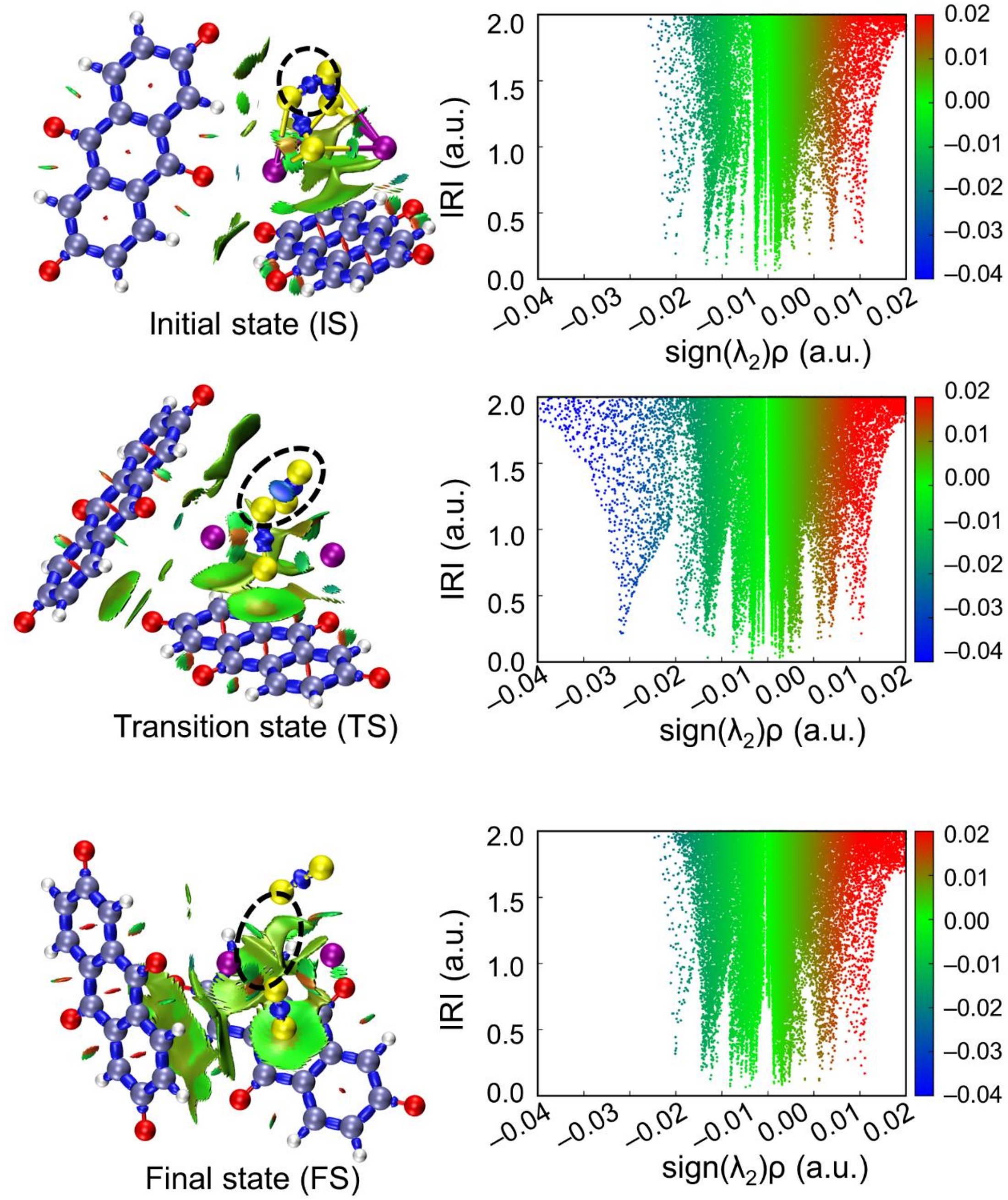

Supplementary Figure 33 | Visualizing chemical bond and non-bond regions by interaction region indicator (IRI) analysis for the intermediates and transition state (TS-B1). The sulfur-sulfur bond is highlighted with black circle and it is broken in final state (FS).

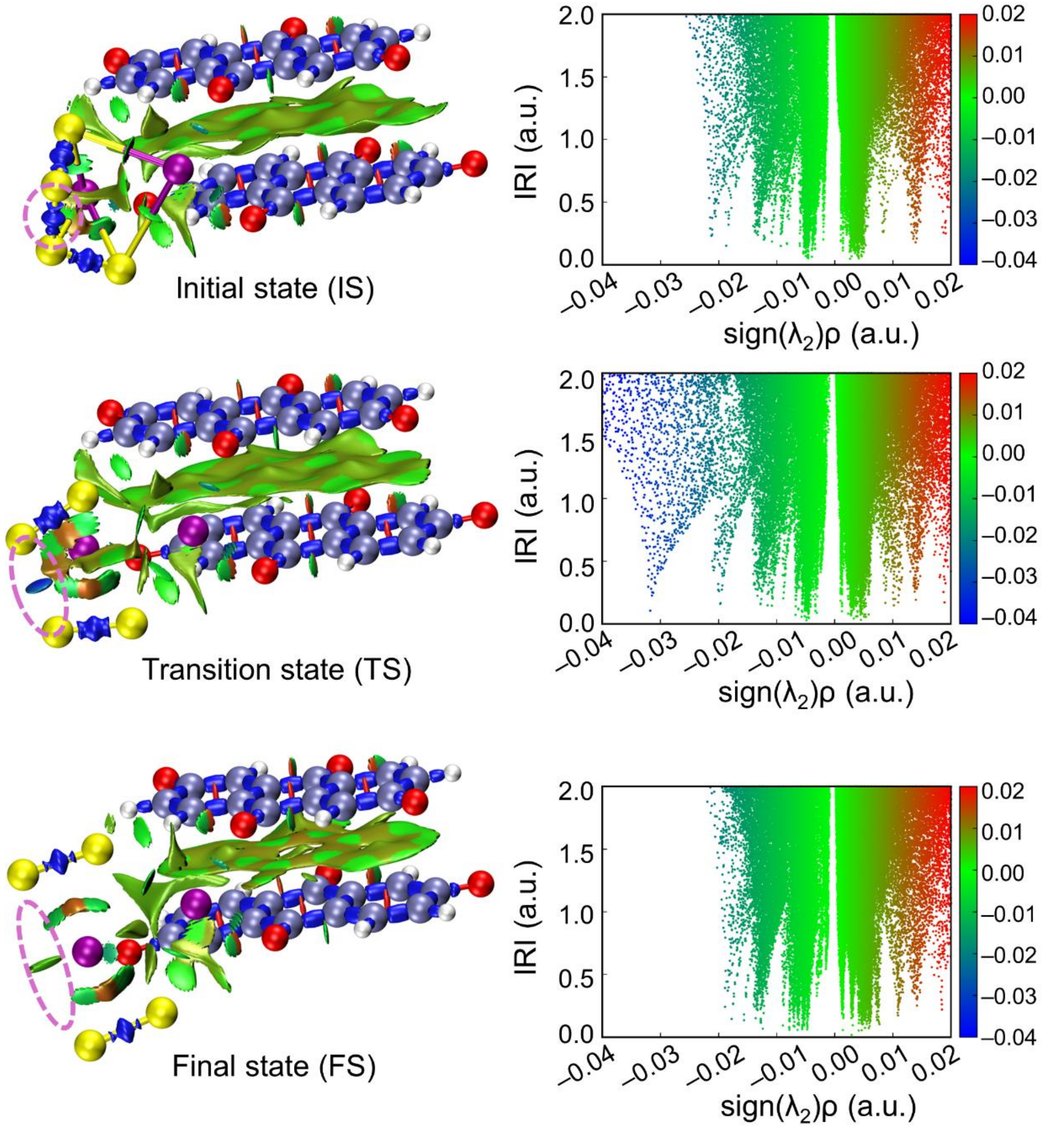

Supplementary Figure 34 | Visualizing chemical bond and non-bond regions by interaction region indicator (IRI) analysis for the intermediates and transition state (TS-C1). The sulfur-sulfur bond is highlighted with purple circle and it is broken in final state (FS).

Supplementary Note 7 | Visualization of chemical bond evolution during 2,6-DHAQ catalyzed polysulfide reduction process

In addition to inner molecular covalent bonding, noncovalent interactions occur between atoms, such as hydrogen bonds, dipole-dipole interactions, and dispersion across a broad range of molecules. These interactions are crucial in the bonding network and are challenging to visualize using conventional methods. Herein, an interaction region indicator (IRI) was introduced to indicate these noncovalent interactions. In Eqn (S8), IRI is essentially the gradient norm of electron density weighted by scaled electron density. The structure was imported into Multiwfn for IRI analysis with the optimization results, and the graph was plotted to obtain a projection of the IRI on the interaction mapping of sign(λ_2)ρ, where ρ represents the electron density and λ_2 denotes the second largest eigenvalue of the Hessian matrix of the electron density. The bonded and non-bonded interactions are distinguished by the sign of λ_2. The bonding area (blue), van der Waals (vdW) forces (green), and spatial effects (red) in the ring structure are all displayed in sign(λ_2)ρ. Weak interactions of the optimized initial states, transition states, and the final states are located in the blue and green intervals (Supplementary Figs. 33-34) using the color projections of sign(λ_2)ρ on the interaction region indicator (IRI) iso-surfaces. Generally, the hydrogen bonds are mapped at $-0.04 < \text{sign}(\lambda_2)\rho < 0$ with light blue iso-surfaces, vdW interactions are mapped at around $\text{sign}(\lambda_2)\rho = 0$ with green iso-surfaces, and steric effects in the ring are mapped at $0 < \text{sign}(\lambda_2)\rho < 0.02$ with red iso-surfaces. The strong covalent bonds are distributed in the region $\text{sign}(\lambda_2)\rho < -0.04$ with blue iso-surfaces between two atoms. Meanwhile, the distribution of different interactions can also be expressed by colored scatter plots. It is easy to recognize the breaking of sulfur-sulfur bonds, which is visualized by the newly appeared blue spike in the transition state and completely disappeared in the final state. In these structures, green regions indicating π-π stacking interaction always exist, proving their significant role in the reaction steps. In Fig. 3f, a blue spike appears at $\text{sign}(\lambda_2)\rho = -0.032$ a.u., which is much lower than the electron density of a chemical bond. Correspondingly a small blue surface appears (marked by a purple circle), indicating the break of the S–S bond. Besides, numerous green spikes and large green surfaces indicate the vdW interaction between K and O atoms ($\text{sign}(\lambda_2)\rho = -0.020$ a.u.), and aromatic rings ($\text{sign}(\lambda_2)\rho \approx 0$ a.u.), suggesting the promoted kinetics by π-π interactions.

$$IRI(r) = \frac{|\nabla \rho(r)|}{\rho(r)^{1.1}} \tag{S8}$$

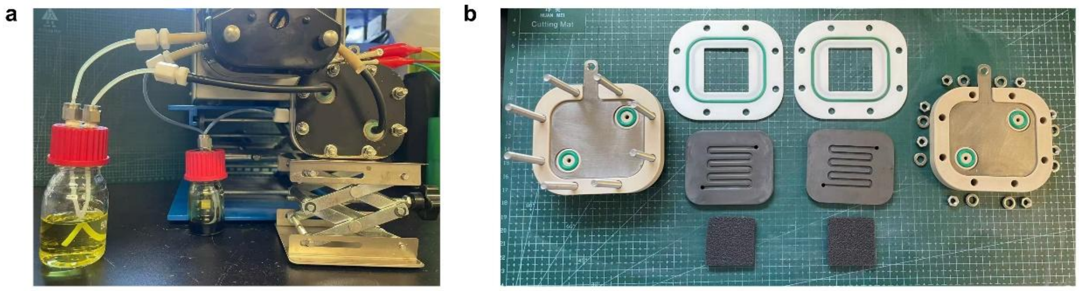

Supplementary Figure 35 | The optical images of (a) the S-Fe flow cell device and (b) cell components.

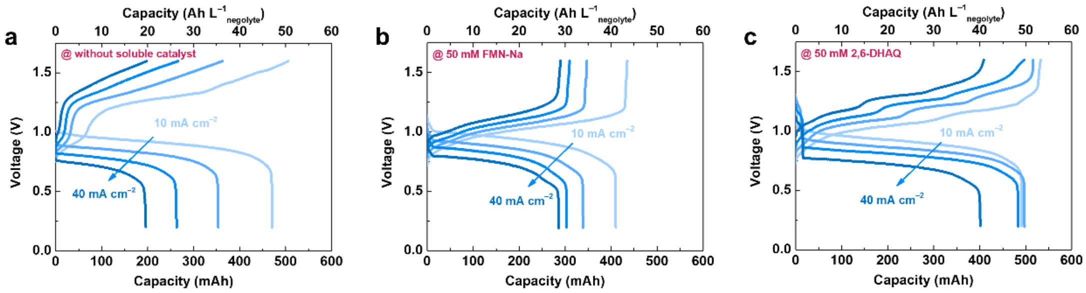

Supplementary Figure 36 | (a) The voltage profiles of S-Fe flow cells without molecular catalysts from 10 to 40 mA cm^{-2} (10 mL of 1 M K_2S_4 –1 M KOH |Nafion 117 membrane| 40 mL of 0.5 M $K_4[Fe(CN)_6]$–1 M KCl). (b) The voltage profiles of S-Fe flow cells with 50 mM FMN-Na from 10 to 40 mA cm^{-2} (10 mL of 1 M K_2S_4 with 50 mM FMN-Na–1 M KOH |Nafion 117 membrane| 40 mL of 0.5 M $K_4[Fe(CN)_6]$–1 M KCl). (c) The voltage profiles of S-Fe flow cells with 50 mM 2,6-DHAQ from 10 to 40 mA cm^{-2} (10 mL of 1 M K_2S_4 with 50 mM 2,6-DHAQ–1 M KOH |Nafion 117 membrane| 40 mL of 0.5 M $K_4[Fe(CN)_6]$–1 M KCl).

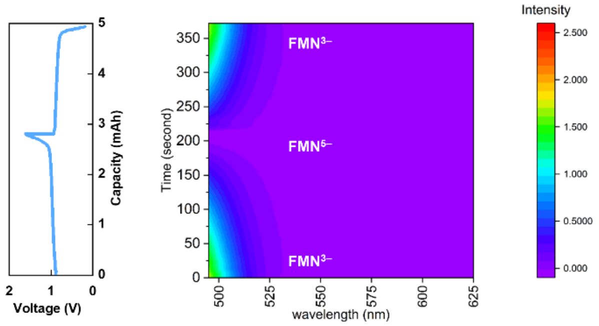

Supplementary Figure 37 | The voltage profiles and in situ UV-vis spectra of 5 mM FMN-Na in a FMN-Na-Fe flow cell at 4 mA cm^{-2} with a cut-off voltage of 1.5 V (10 mL of 5 mM FMN-Na–1 M KOH |Nafion 117 membrane| 15 mL of 0.25 M $K_4[Fe(CN)_6]$–1 M KCl). The operando tests were conducted at 24 ± 1 °C.

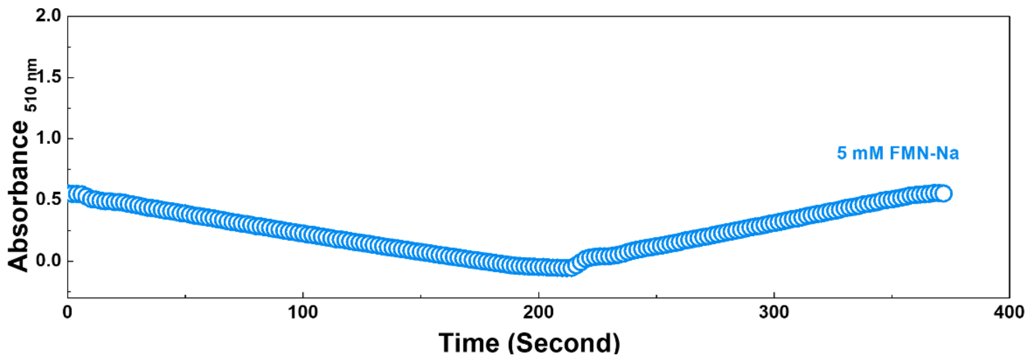

Supplementary Figure 38 | The absorbance of 5 mM FMN-Na at 510 nm.

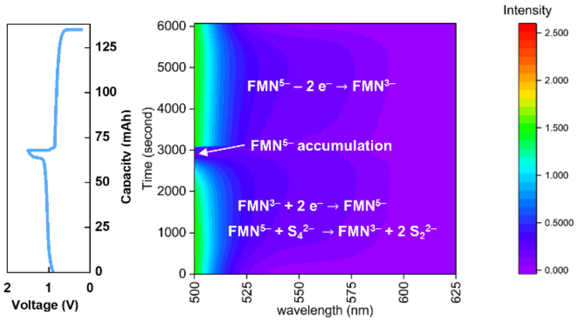

Supplementary Figure 39 | The voltage profiles and operando UV-vis spectra of 250 mM K_2S_4 with 5 mM FMN-Na in a S-Fe flow cell at 20 mA cm^{-2} with a cut-off voltage of 1.5 V (10 mL of 250 mM K_2S_4 with 5 mM FMN-Na–1 M KOH |Nafion 117 membrane| 15 mL of 0.25 M $K_4[Fe(CN)_6]$–1 M KCl). The operando tests were conducted at 24 ± 1 °C.

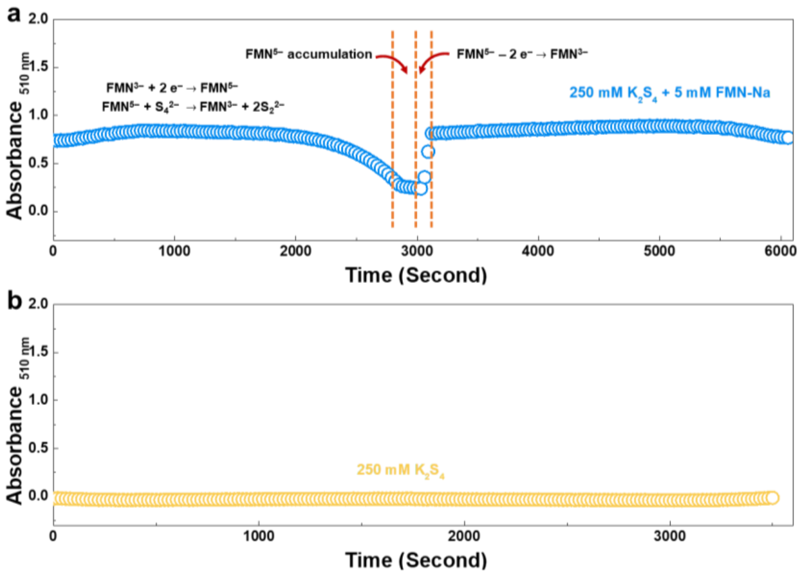

Supplementary Figure 40 | (a) The absorbance of 250 mM K_2S_4 with 5 mM FMN-Na at 510 nm. (b) The absorbance of 250 mM K_2S_4 at 510 nm.

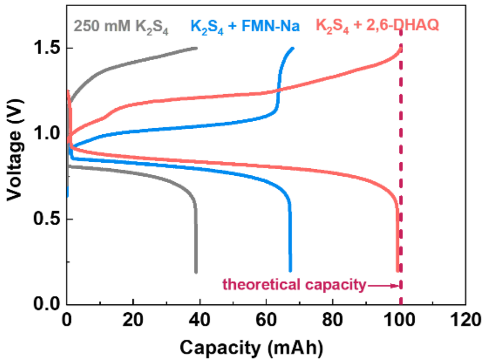

Supplementary Figure 41 | The voltage profile of the polysulfide-ferrocyanide (S-Fe) redox flow battery with a cut-off voltage of 1.5 V (10 mL of 0.25 M K_2S_4–1 M KOH with or without 5 mM molecular catalyst |Nafion 117 membrane| 15 mL of 0.25 M $K_4[Fe(CN)_6]$–1 M KCl). The theoretical capacity of this cell is 100.5 mAh.

Supplementary Note 8 | The origin of limited polysulfide utilization of FMN-Na molecular catalyst.

FMN-Na possesses a suitable potential (–0.52 V vs. SHE) and fast kinetics behaviors, making it a perfect catalyst candidate[4,5]. This homogeneous electron transfer route converts the sluggish electrochemical process into a fast chemical process between reduced FMN^{5-} and S_4^{2-} (Eqn (S9-S10)).

$$FMN^{3-} + 2\ e^{-} \rightarrow FMN^{5-} \quad (S9)$$

$$FMN^{5-} + S_4^{2-} \rightarrow FMN^{3-} + 2\ S_2^{2-} \quad (S10)$$

We conducted operando UV-vis spectra to carefully study the origin of the insufficient polysulfide capacity utilization. We could clearly differentiate oxidized FMN^{3-} due to the $\pi \rightarrow \pi^*$ transitions of flavin structure[6], while FMN^{5-} shows no absorbance in 500 - 625 nm. For the 5 mM FMN-Na-ferrocyanide cell, the intensity at 510 nm linearly decreased with the charging time and increased back at the end of discharge, suggesting the reversible transition from FMN^{3-} to FMN^{5-} during charge and converting back to its original state after discharge (Supplementary Figs. 37-38). Interestingly, for the K_2S_4 negolyte with 5 mM FMN-Na, the intensity of FMN^{3-} remained stable in the initial stage because the reduced FMN^{5-} is quickly chemically oxidized to FMN^{3-} (Supplementary Figs. 39-40). However, the intensity gradually decreased at higher SOC, indicating that the regenerated rate of FMN^{3-} could not catch up the current density. At the end of the charge, the intensity of FMN^{3-} almost disappears, meaning that the depletion of FMN^{3-} and the accumulation of the reduced FMN^{5-}, which leads to the pre-termination of the charging process (Supplementary Figs. 39-40). Even though FMN-Na molecular catalysts showed excellent catalytic performance with decrease overpotential, the catalytic rate is insufficient to achieve high polysulfide utilization (Supplementary Fig. 41). The achieved capacity (67.2 mAh) is lower than the full capacity (100.5 mAh) of this cell.

Since we found that the homogeneous electron transfer rate of FMN-Na molecular catalysts has a linear relationship with S_4^{2-} concentration[5], we assumed this limited capacity utilization comes from a low reaction rate at high SOC with low S_4^{2-} concentration. At low SOC with high S_4^{2-} concentration, the regeneration rate is faster than the electron input rate (only depends on current density) so we didn't observe FMN^{3-} intensity change initially. With increasing the SOC, the chemical reaction rate gradually decreases until it could not catch up to the electron input rate, from which moment the chemical reaction between reduced molecular catalysts and S_4^{2-} starts to be the rate-determining step. The accumulation of reduced molecular catalysts results in the pre-termination of the charging process with low capacity utilization. Thus, exploiting molecular catalysts with a higher catalytic rate is believed to increase polysulfide utilization.

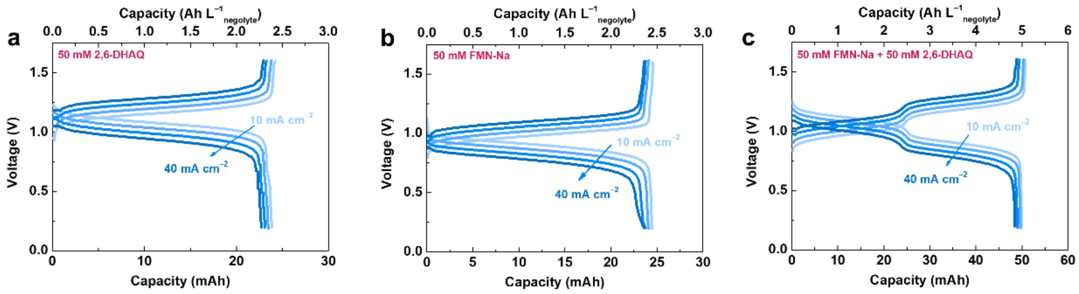

Supplementary Figure 42 | (a) The voltage profile of 50 mM 2,6-DHAQ-ferrocyanide flow cell. (10 mL of 50 mM 2,6-DHAQ–1 M KOH |Nafion 117 membrane| 10 mL of 0.5 M $K_4[Fe(CN)_6]$–1 M KCl). (b) The voltage profile of 50 mM FMN-Na-ferrocyanide flow cell. (10 mL of 50 mM FMN-Na–1 M KOH |Nafion 117 membrane| 10 mL of 0.5 M $K_4[Fe(CN)_6]$–1 M KCl). (c) The voltage profile of 50 mM FMN and 50 mM 2,6-DHAQ-Na-ferrocyanide flow cell. (10 mL of 50 mM FMN-Na and 50 mM 2,6-DHAQ–1 M KOH |Nafion 117 membrane| 10 mL of 0.5 M $K_4[Fe(CN)_6]$–1 M KCl).

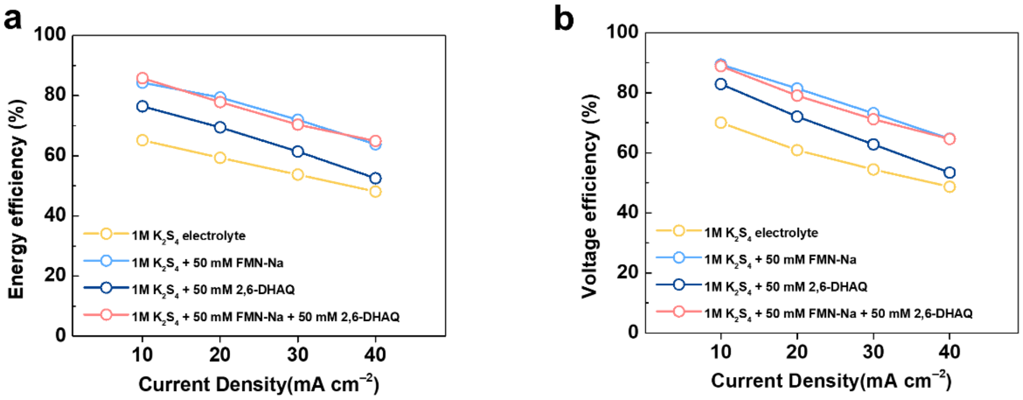

Supplementary Figure 43 | (a) The energy efficiency (EE) of S-Fe flow cells with or without molecular catalysts from 10 to 40 mA cm^{-2}. (b) The voltage efficiency (VE) of S-Fe flow cells with or without molecular catalysts from 10 to 40 mA cm^{-2}.

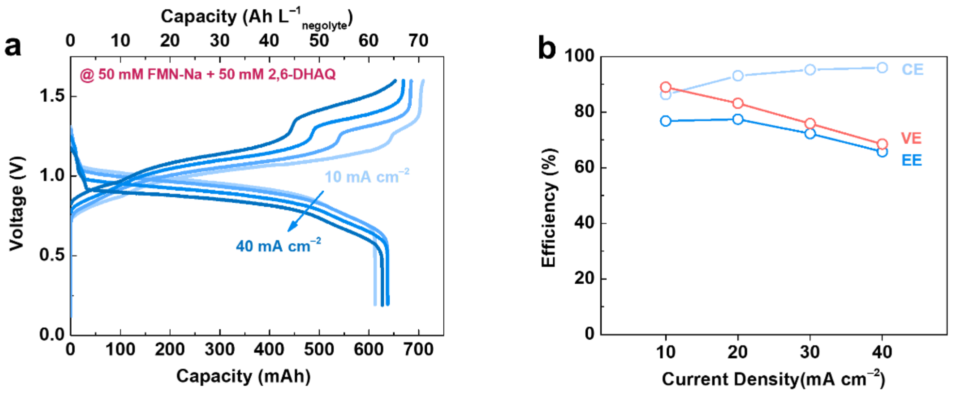

Supplementary Figure 44 | (a) The voltage profiles of high 1.2 M S-Fe flow cell with relay catalysts from 10 to 40 mA cm^{-2} (10 mL of 1.2 M K_2S_4 with 50 mM FMN-Na + 50 mM 2,6-DHAQ–1 M KOH |Nafion 117 membrane| 48 mL of 0.5 M $K_4[Fe(CN)_6]$–1 M KCl). (b) The coulombic efficiency, EE, and VE of the 1.2 M S-Fe flow cell.

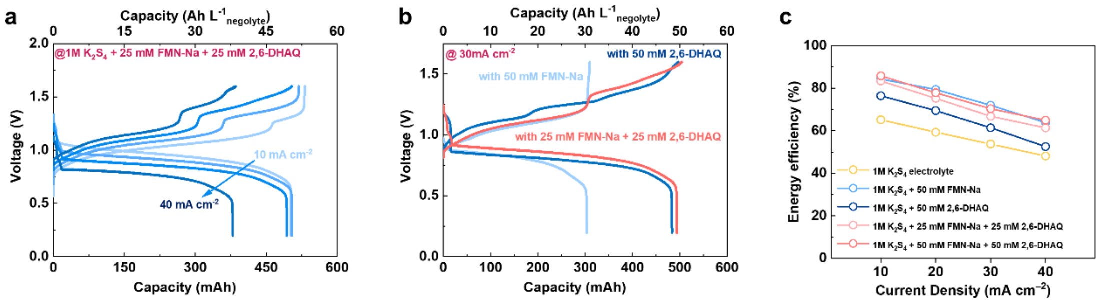

Supplementary Figure 45 | (a) The voltage profiles of the S-Fe flow cell with relay catalysts from 10 to 40 mA cm^{-2} (10 mL of 1 M K_2S_4 with 25 mM FMN-Na and 25 mM 2,6-DHAQ–1 M KOH |Nafion 117 membrane| 40 mL of 0.5 M $K_4[Fe(CN)_6]$–1 M KCl). (b) The voltage profiles of the S-Fe flow cell with 50 mM molecular catalyst at 30 mA cm^{-2}. (c) The energy efficiency comparison of the S-Fe flow cell with 50 mM molecular catalysts.

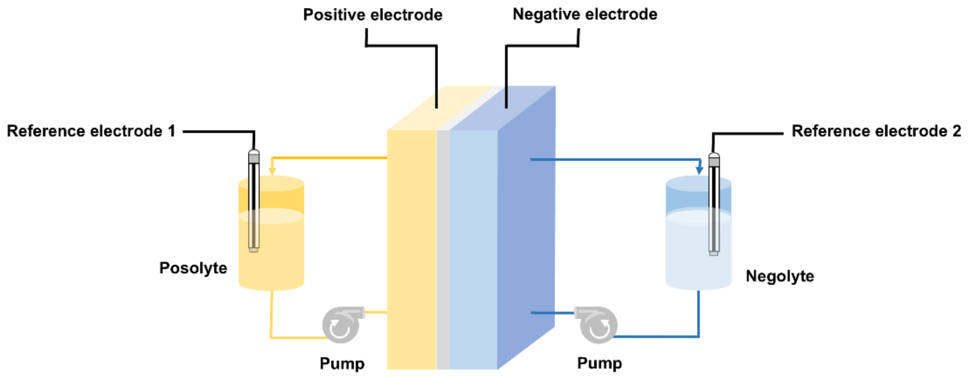

Supplementary Figure 46 | The schematic illustration of the four-electrode test.

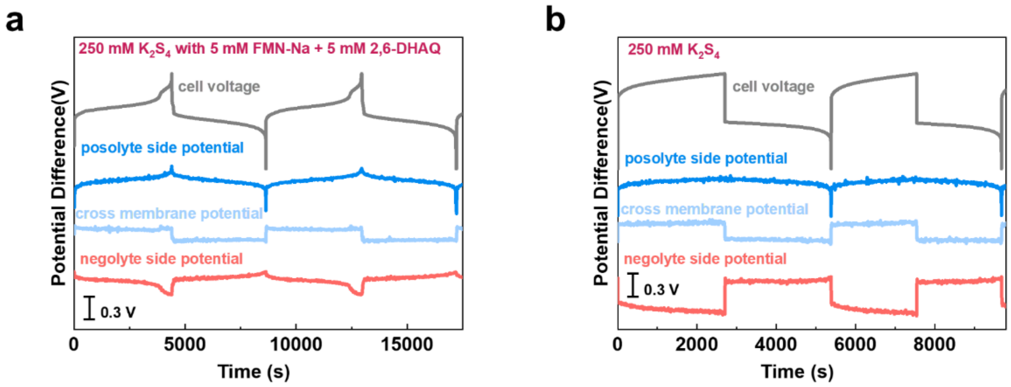

Supplementary Figure 47 | The four-electrode voltage profile of S-Fe flow cell (a) with relay catalysts and (b) without molecular catalysts at 20 mA cm^{-2} (10 mL of 250 mM K_2S_4 with or without 5 mM FMN-Na + 5 mM 2,6-DHAQ–1 M KOH |Nafion 117 membrane| 15 mL of 0.25 M $K_4[Fe(CN)_6]$–1 M KCl).

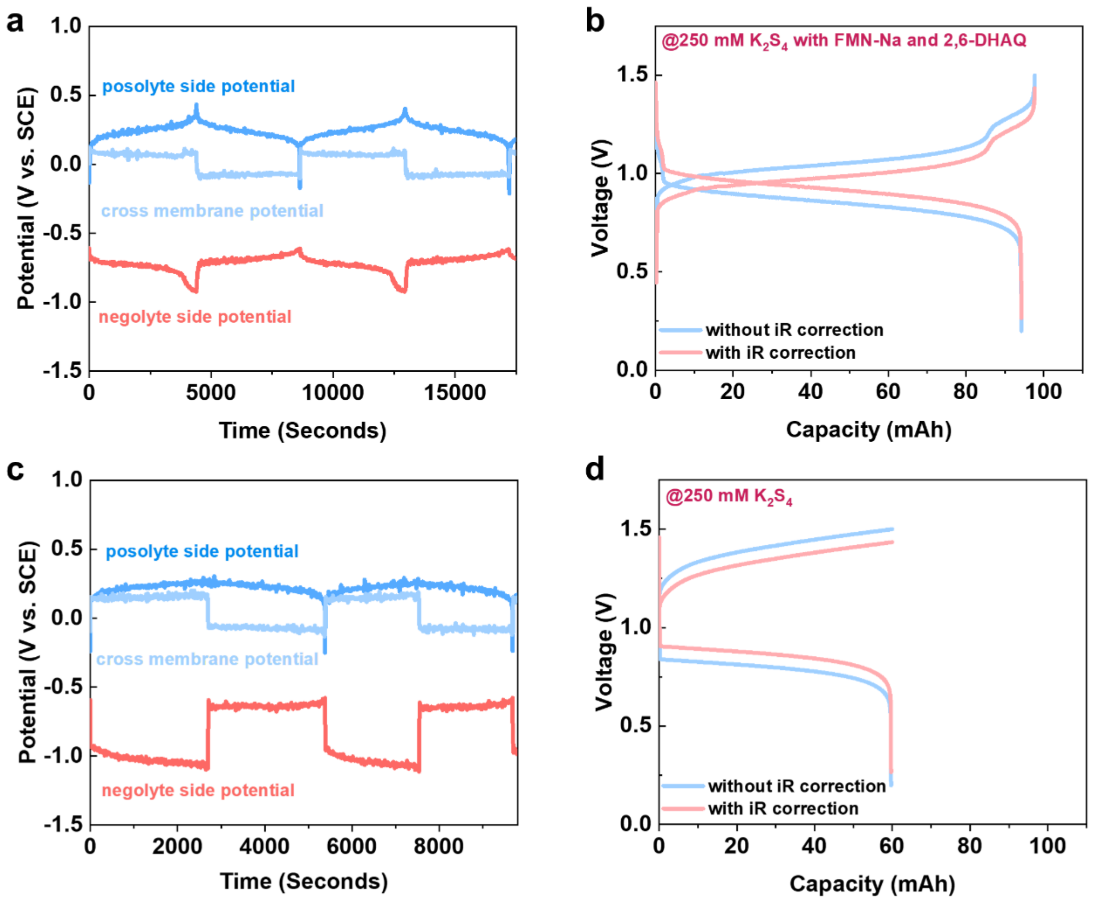

Supplementary Figure 48 | (a) The four-electrode voltage profile (vs. SCE) of the S-Fe flow cell with relay catalysts. (b) The iR correction for the S-Fe flow cell with relay catalysts. (c) The four-electrode voltage profile (vs. SCE) of the S-Fe flow cell without relay catalysts. (d) The iR correction for the S-Fe flow cell without relay catalysts. The resistance of the Nafion 117 membrane in the S-Fe flow cell[5] is around 3.3 Ω cm^2.

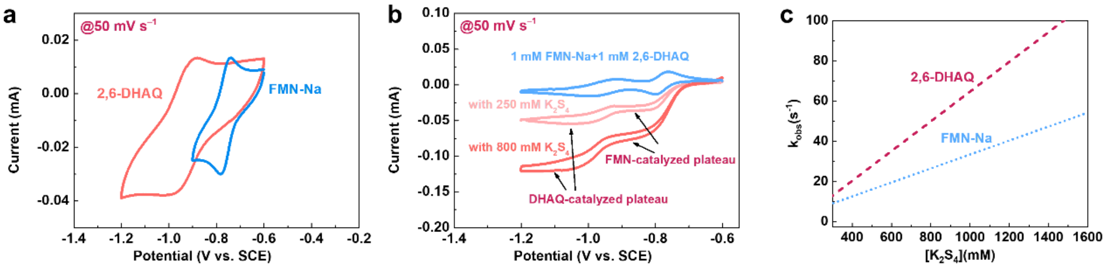

Supplementary Figure 49 | (a) The CVs of individual 1 mM 2,6-DHAQ and 1 mM FMN-Na. (b) The modified CV plot of the mixed solution of 1 mM FMN-Na + 1 mM 2,6-DHAQ with 250 mM or 800 mM K_2S_4 after removing the background current. (c) The fitted k_{obs} of 2,6-DHAQ and FMN-Na[5] over polysulfide concentration.

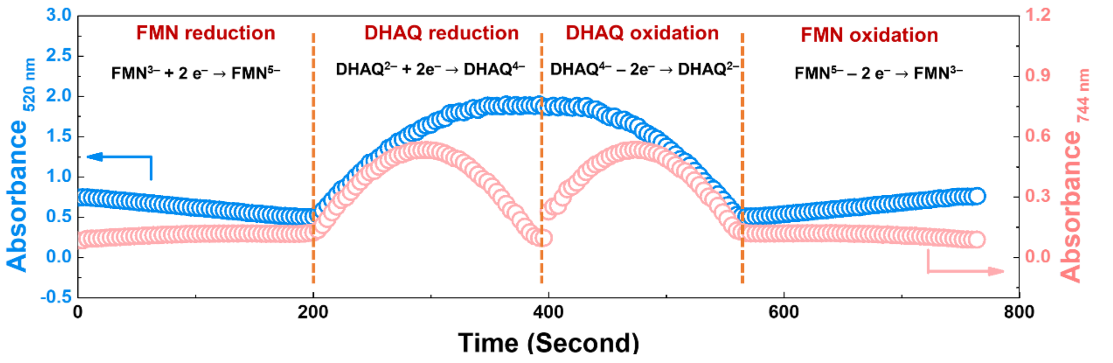

Supplementary Figure 50 | The absorbance of 5 mM 2,6-DHAQ and 5 mM FMN-Na at 520 nm (blue line) and 744 nm (pink line).

Supplementary Note 9 | Operando UV-vis spectra of mixed electrolyte of 5 mM FMN and 5 mM 2,6-DHAQ.

Fortunately, even though the absorbance of FMN-Na and 2,6-DHAQ are all detected from 500 nm to 600 nm, their totally different evolution trend and their perfectly matched intensity make it possible for us to differentiate them. For the 2,6-DHAQ and FMN-Na mixed cell, the absorbance at 520 nm keeps decreasing in the first half charging process, corresponding to the reduction of FMN^{3-} to FMN^{5-} (Fig. 4e-f and Supplementary Fig. 50). There is no change in 744 nm in this region. Then, after the turning point (between two charging plateaus) appears in the voltage profile (Fig. 4d), the absorbance at 520 nm starts to increase, while the absorbance at 744 nm shows an initial increase followed by a decrease, reflecting the two-step reduction from $DHAQ^{2-}$ to $DHAQ^{3-}$ intermediate, and then to $DHAQ^{4-}$ (Fig. 4f and Supplementary Fig. 50). The discharge process evolves in the converse way (Fig. 4e-f and Supplementary Fig. 50).

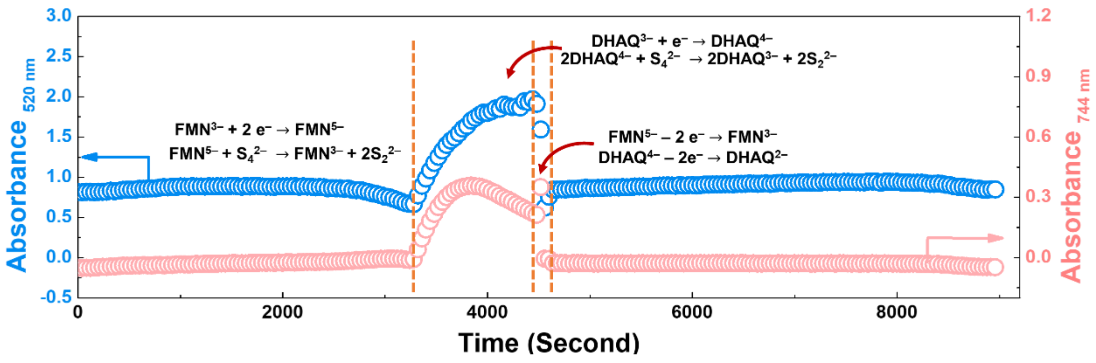

Supplementary Figure 51 | The absorbance of 250 mM K_2S_4 with 5 mM 2,6-DHAQ at 520 nm (blue line) and 744 nm (pink line). The corresponding reaction equation is shown in Fig. 4h-i.

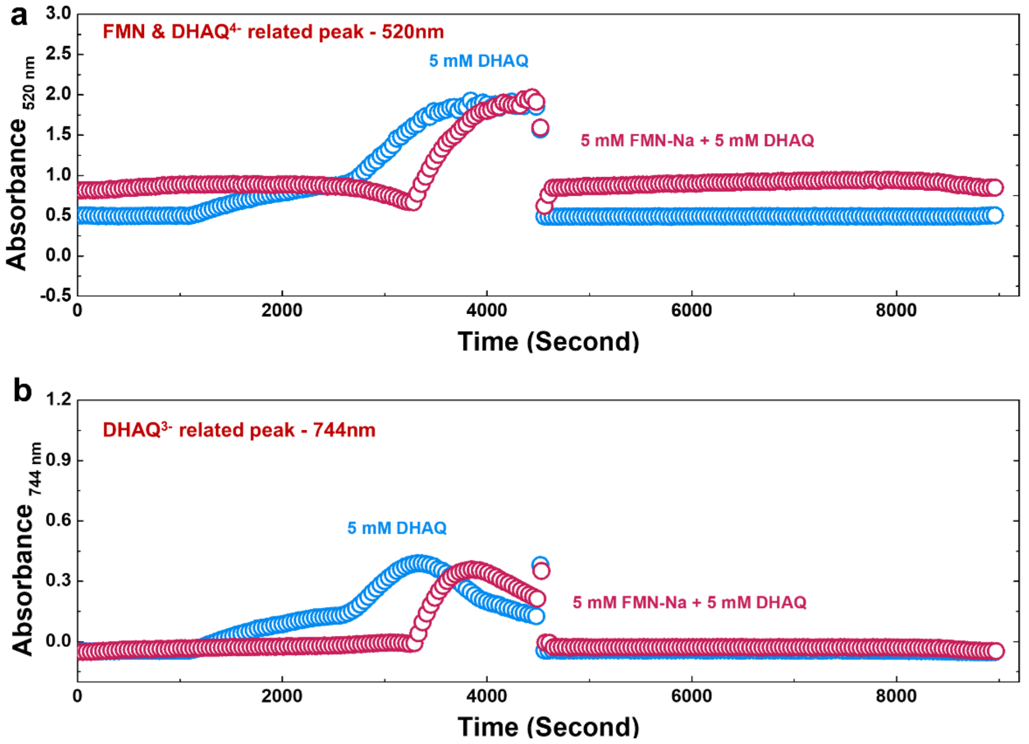

Supplementary Figure 52 | The absorbance comparison of 250 mM K_2S_4 + 5 mM 2,6-DHAQ negolyte with (deep red line) or without 50 mM FMN-Na (blue line) at (a) 520 nm and (b) 744 nm.

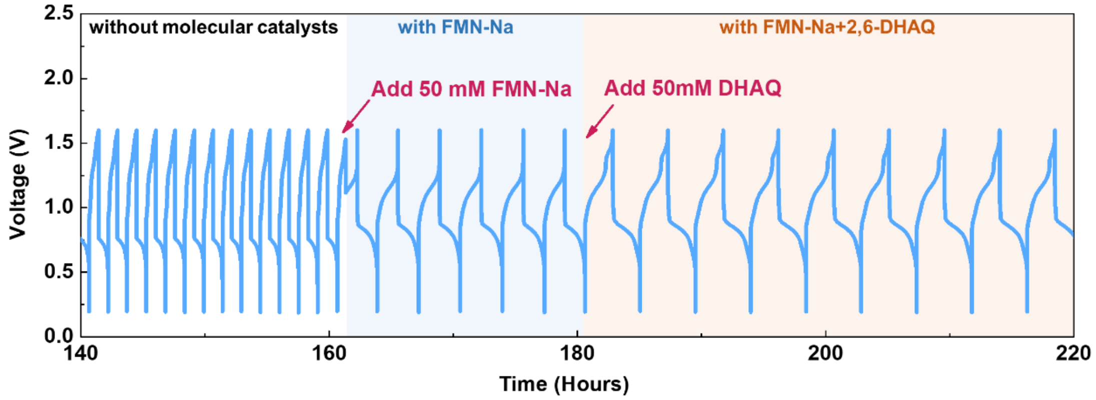

Supplementary Figure 53 | The voltage profiles of stepwise adding 50 mM FMN-Na and 50 mM 2,6-DHAQ to the un-catalyzed S-Fe flow cell after 100 cycles at 20 mA cm^{-2}. (10 mL of 1 M K_2S_4–1 M KOH |CRIS membrane| 40 mL of 0.5 M $K_4[Fe(CN)_6]$–1 M KCl). The 50 mM FMN-Na and 50 mM 2,6-DHAQ were added at the 100th cycle and 106th cycle.

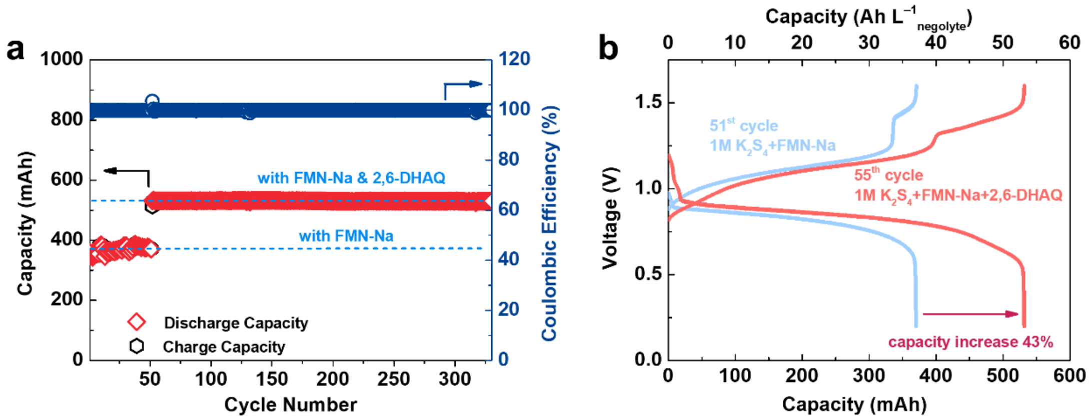

Supplementary Figure 54 | (a) The capacity and Coulombic efficiency of the S-Fe flow cell after adding 50 mM 2,6-DHAQ to the cell with 50 mM FMN-Na (10 mL of 1 M K_2S_4 with 50 mM FMN-Na (and 50 mM 2,6-DHAQ after 52nd cycle)–1 M KOH |CRIS membrane| 40 mL of 0.5 M $K_4[Fe(CN)_6]$–1 M KCl). (b) The voltage profiles of the S-Fe flow cell before and after adding 2,6-DHAQ.

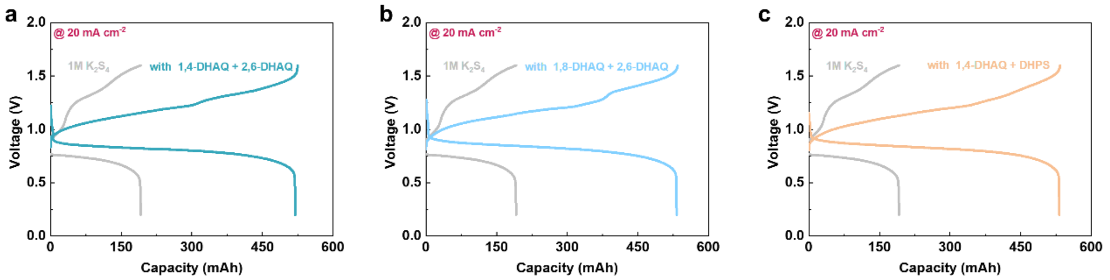

Supplementary Figure 55 | (a) The voltage profiles of S-Fe flow cells with or without relay catalysts at 20 mA cm^{-2} (10 mL of 1 M K_2S_4 with or without 10 mM 1,4-DHAQ and 10 mM 2,6-DHAQ–1 M KOH |CRIS membrane| 40 mL of 0.5 M $K_4[Fe(CN)_6]$–1 M KCl). (b) The voltage profiles of S-Fe flow cells with or without relay catalysts at 20 mA cm^{-2} (10 mL of 1 M K_2S_4 with or without 10 mM 1,8-DHAQ and 10 mM 2,6-DHAQ–1 M KOH |CRIS membrane| 40 mL of 0.5 M $K_4[Fe(CN)_6]$–1 M KCl). (c) The voltage profiles of S-Fe flow cells with or without relay catalysts at 20 mA cm^{-2} (10 mL of 1 M K_2S_4 with or without 10 mM 1,4-DHAQ and 10 mM DHPS–1 M KOH |CRIS membrane| 40 mL of 0.5 M $K_4[Fe(CN)_6]$–1 M KCl).

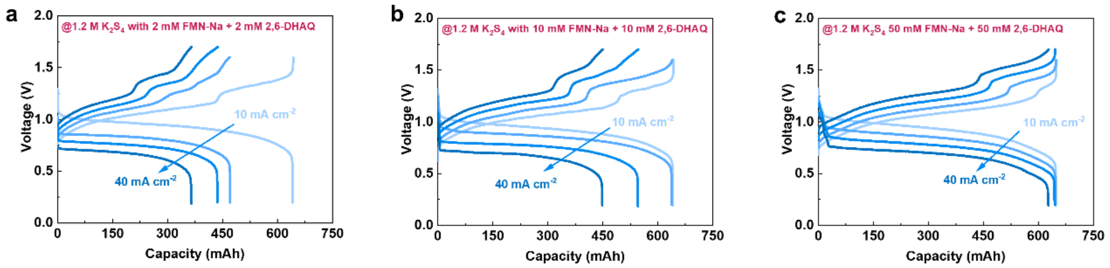

Supplementary Figure 56 | The voltage profiles of 1.2 M S-Fe flow cells with (a) 2 mM FMN-Na + 2 mM 2,6-DHAQ, (b) 10 mM FMN-Na + 10 mM 2,6-DHAQ, and (c) 50 mM FMN-Na + 50 mM 2,6-DHAQ from 10 to 40 mA cm^{-2} (10 mL of 1.2 M K_2S_4 with FMN-Na and 2,6-DHAQ–1 M KOH |Nafion 117 membrane| 24 mL of 1 M ferrocyanide posolyte (0.5 M $K_4[Fe(CN)_6]$ + 0.5 M $Na_4[Fe(CN)_6]$).

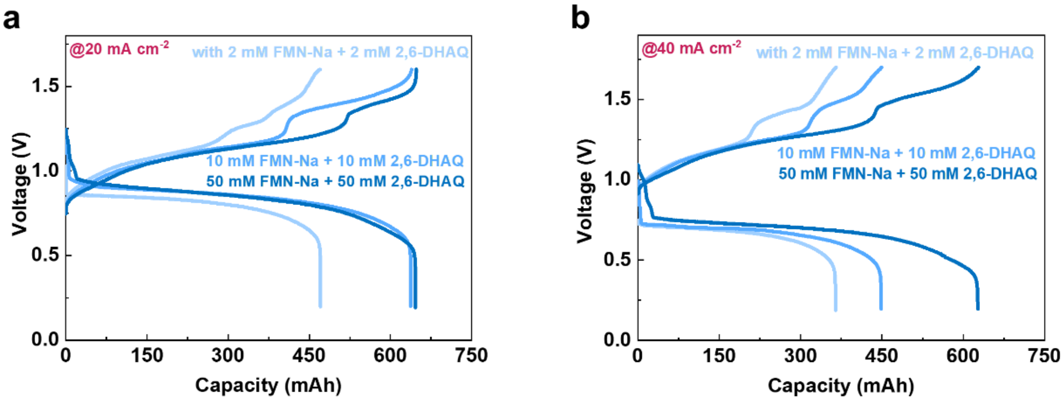

Supplementary Figure 57 | The voltage profiles of 1.2 M S-Fe flow cells with different concentrations of relay catalysts at (a) 20 mA cm^{-2} and (b) 40 mA cm^{-2}.

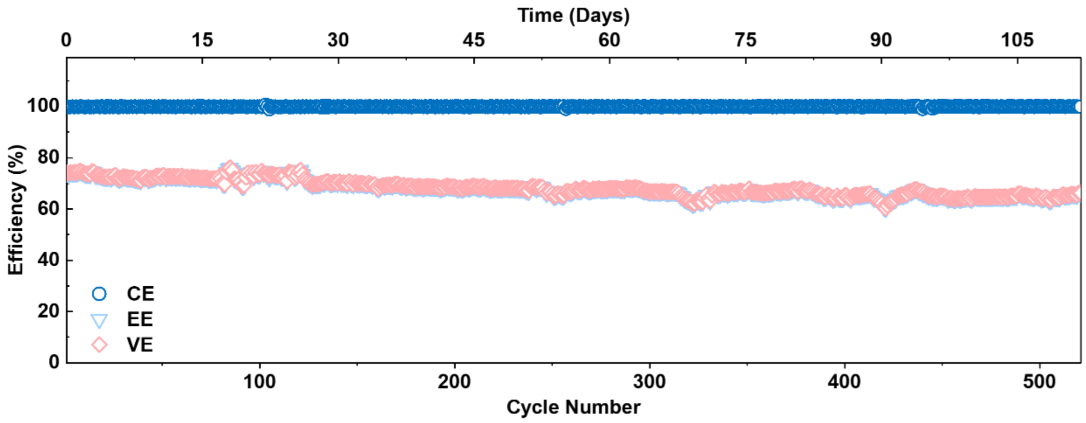

Supplementary Figure 58 | The CE, EE, and VE of the 1.2 M S-Fe flow cell.

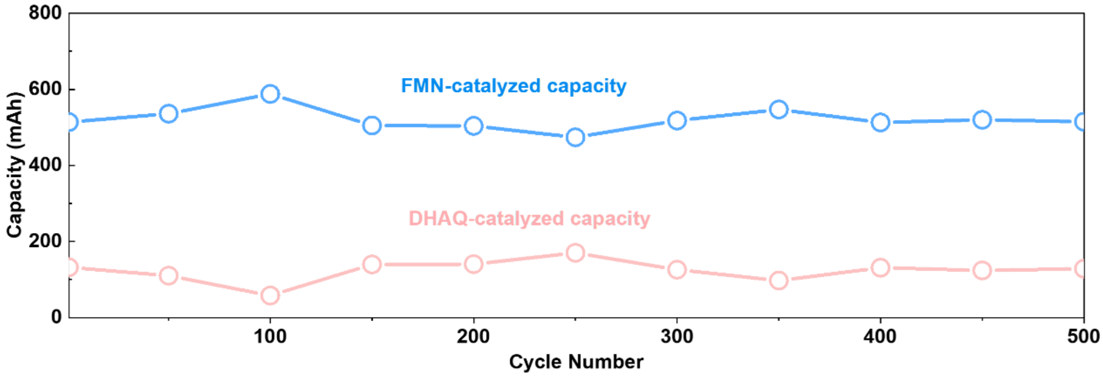

Supplementary Figure 59 | The capacity contribution from two charging plateaus over 500 cycles. The charging capacity below 1.3 V is ascribed to FMN-catalyzed capacity, and the charging capacity above 1.3 V is ascribed to DHAQ-catalyzed capacity. The small fluctuations are ascribed to temperature change over the 112 days.

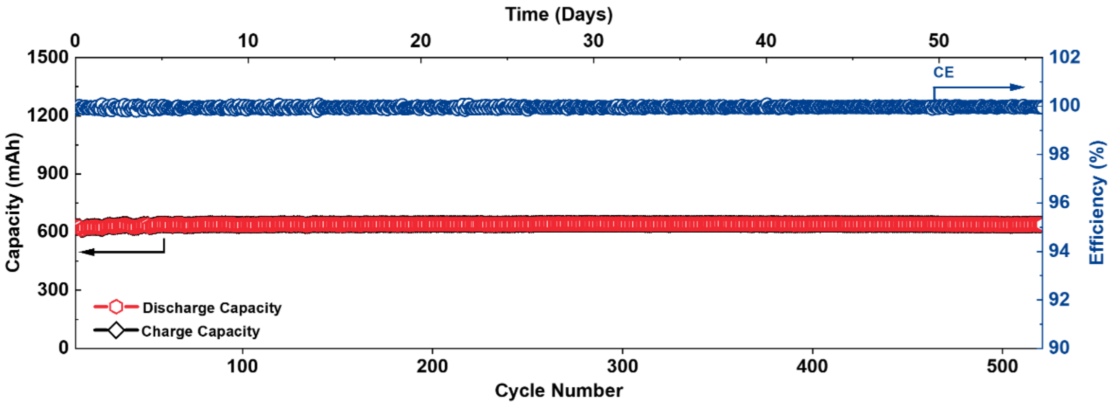

Supplementary Figure 60 | The long-term cycling stability of high-concentration S-Fe flow cell at 40 mA cm^{-2} with a cut-off voltage of 1.7 V and 0.2 V (10 mL of 1.2 M K_2S_4–1 M KOH with 50 mM FMN-Na and 50 mM 2,6-DHAQ |CRIS membrane| 24 mL of 1 M ferrocyanide posolyte (0.5 M $K_4[Fe(CN)_6]$ + 0.5 M $Na_4[Fe(CN)_6]$).

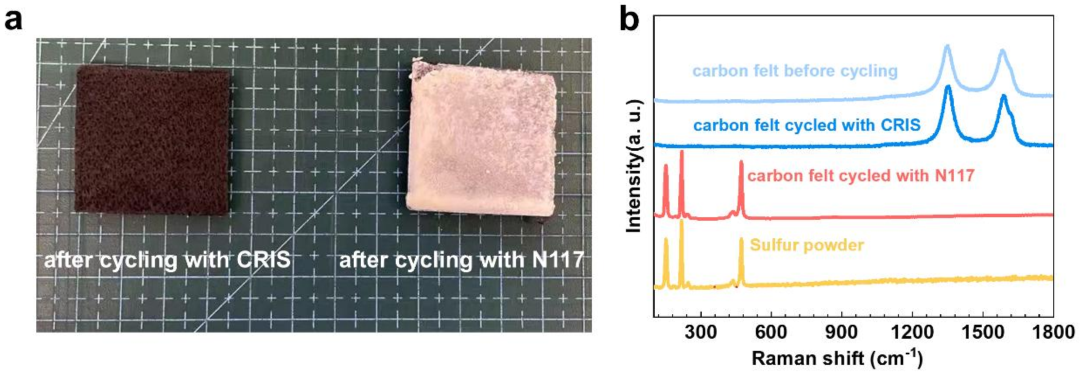

Supplementary Figure 61 | (a) The optical images of carbon felts after cycling (500 cycles) with CRIS membrane (left one) or Nafion 117 membrane (right one). (b) The Raman spectra of pristine carbon felt, sulfur powder, and carbon felt after cycling.

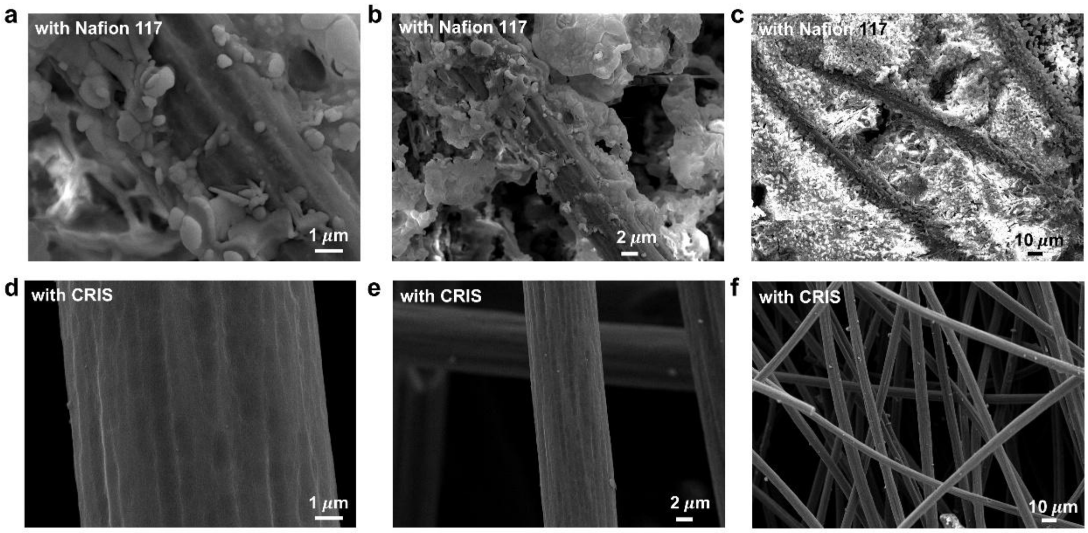

Supplementary Figure 62 | The SEM images of carbon felt on the positive side after cycling (500 cycles) with (a-c) Nafion 117 membrane or (d-f) CRIS membrane.

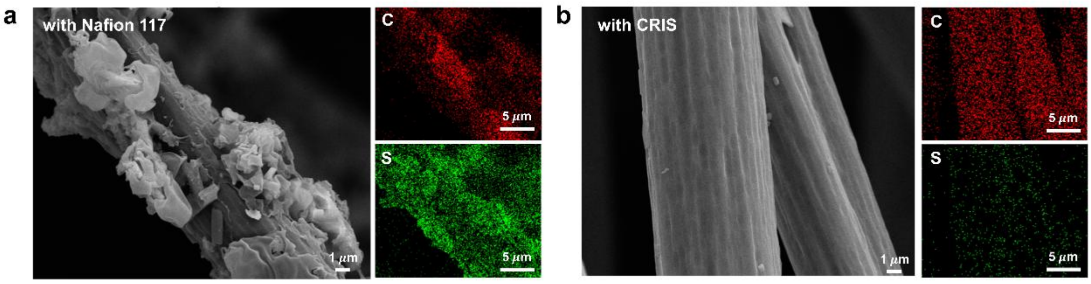

Supplementary Figure 63 | The SEM image and EDX mapping of solid deposited on the carbon fiber on the positive side after cycling (500 cycles) with (a) Nafion 117 membrane or (b) CRIS membrane. The carbon elements are labeled with red color and sulfur elements are labeled with green color.

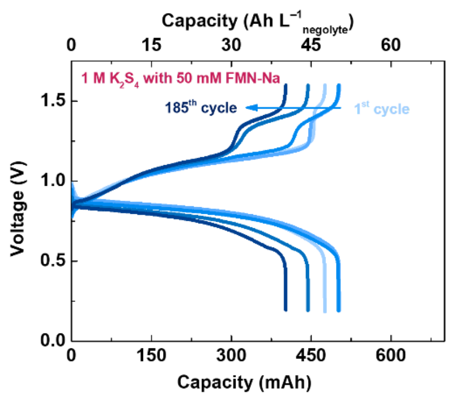

Supplementary Figure 64 | The voltage profiles of the high-concentration S-Fe flow cell with FMN-Na single molecular catalyst.

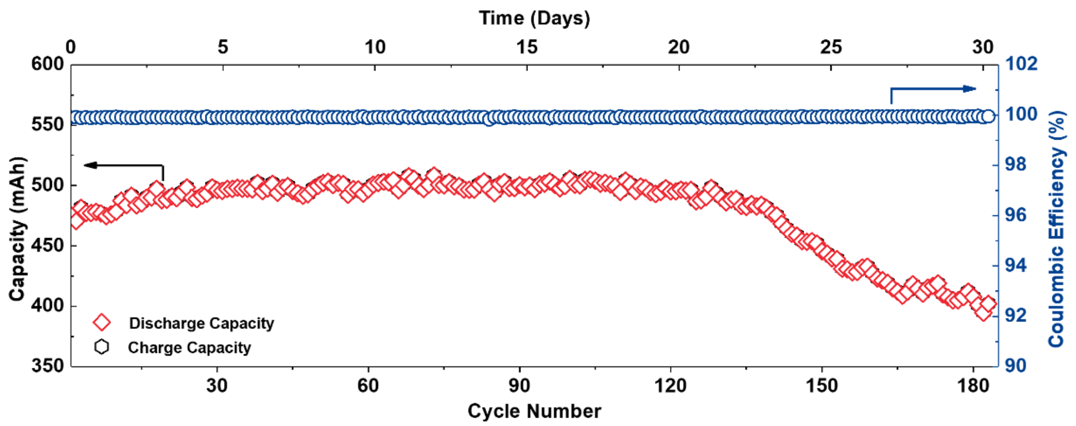

Supplementary Figure 65 | Capacity retention and coulombic efficiency of the high-concentration S-Fe flow cell with FMN-Na single molecular catalyst.

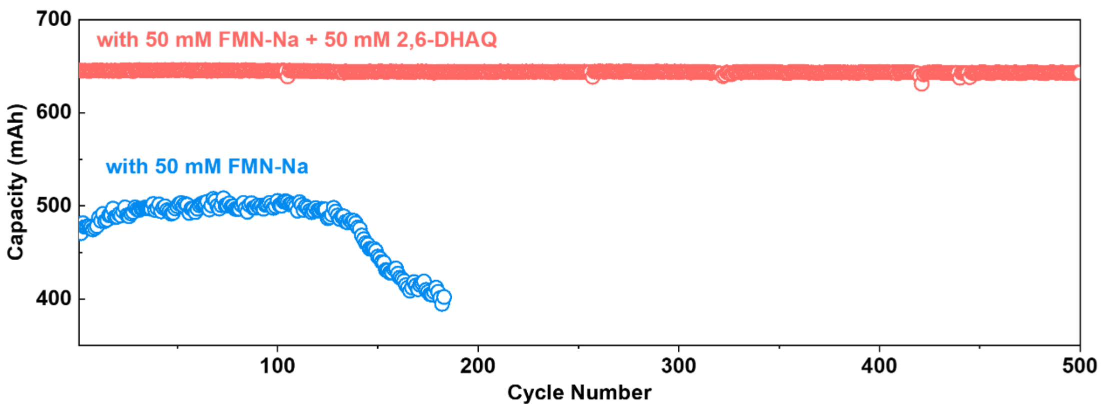

Supplementary Figure 66 | Comparison of the cycling stability of the high concentration S-Fe cell with FMN-Na single catalyst and FMN+DHAQ relay catalysts.

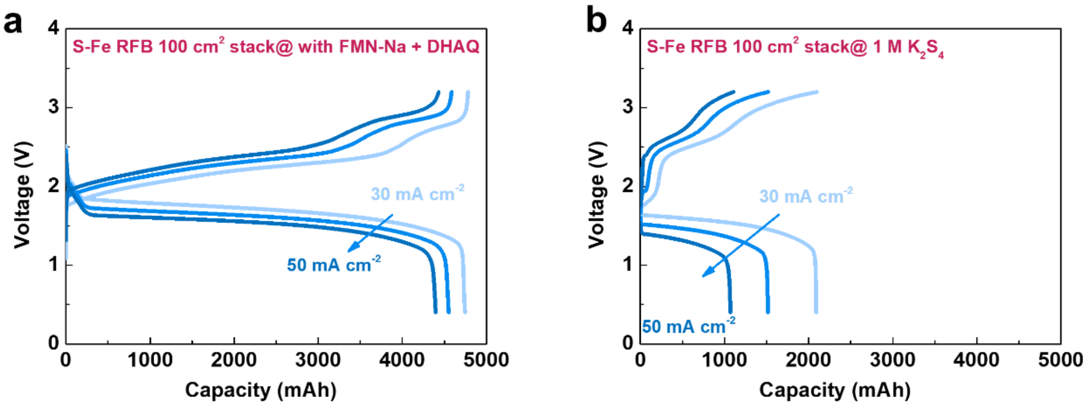

Supplementary Figure 67 | Voltage profiles of 100 cm^2 S-Fe flow cell stack (a) with and (b) without relay catalysts from 3 A (30 mA cm^{-2}) to 5 A (50 mA cm^{-2}).

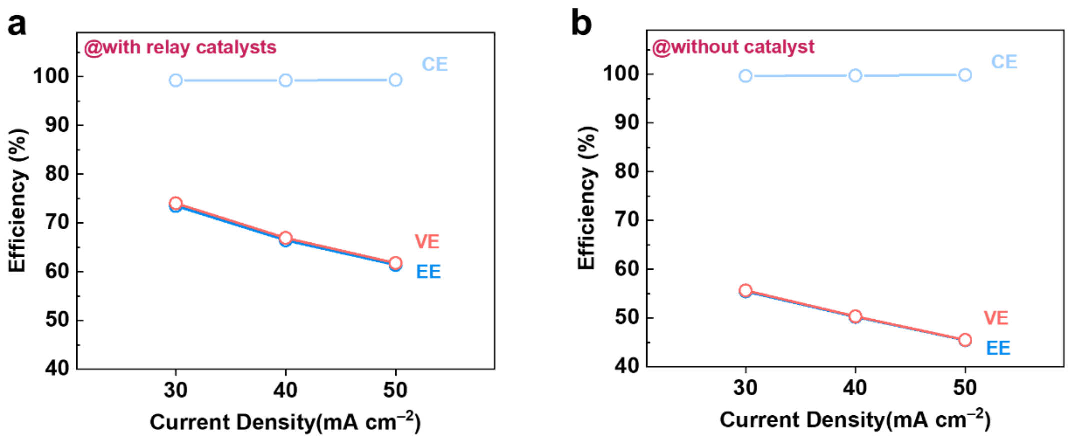

Supplementary Figure 68 | (a) The CE, EE, and VE of the 100 cm^2 S-Fe flow cell stack with relay catalysts from 30 to 50 mA cm^{-2}. (b) The CE, EE, and VE of the 100 cm^2 S-Fe flow cell stack without catalysts from 30 to 50 mA cm^{-2}.

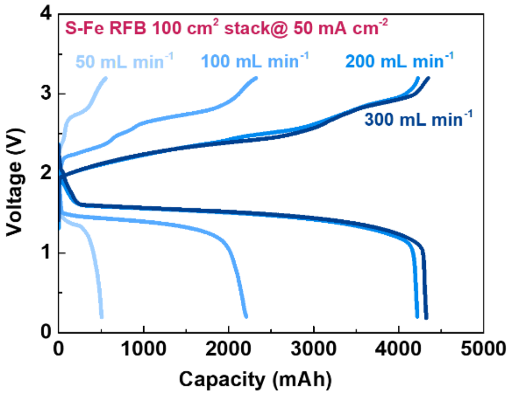

Supplementary Figure 69 | Voltage profiles of the 100 cm^2 S-Fe flow cell stack at flow rates from 50 mL min^{-1} to 300 mL min^{-1}.

Supplementary Note 10 | Temperature effect of relay catalysts.

We examined the influence of temperature on relay catalysis between 10 – 40 ℃. At the fixed current density of 20 mA cm^{-2}, we increased the temperature from 10 ℃ to room temperature (21 ℃), and then to 40 ℃ to study the temperature effect in S-Fe RFB with or without relay catalysts (Supplementary Figs. 70-71). For the cell without relay catalysts, the K_2S_4 electrolyte showed a very high overpotential and low capacity at 10 ℃ (Supplementary Fig. 70). When the temperature is elevated to room temperature, the capacity increases with a low-voltage plateau (related to long-chain polysulfide, e.g. S_6^{2-}) appeared. At 40 ℃, the cell achieved a higher capacity, which could be attributed to the generation of more long-chain polysulfide. However, high overpotentials were observed at all temperatures. In contrast, cells with relay catalysts achieved full capacity utilization across the entire temperature range (10 – 40 ℃). The capacity contribution from FMN-Na itself is only around 2 Ah L^{-1} which accounts for 3.7% of the total capacity, suggesting that the capacity enhancement and energy efficiency improvements are mainly from the catalysis effect (Supplementary Fig. 72). At 10 ℃, the plateau of FMN is shortened compared to room temperature, while full capacity was still achieved at the plateau of 2,6-DHAQ, which is consistent with faster reaction kinetics of 2,6-DHAQ catalyst (75 s^{-1} at 1 M K_2S_4). The kinetics of both catalysts are promoted at elevated temperatures with decreased overpotentials. Nevertheless, temperature affects not only the homogeneous chemical reaction rate, but also affects the electrolyte conductivity, viscosity, polysulfide composition, membrane pore size, etc., all of which synergistically result in improved overall cell performance at higher temperatures. The EIS spectra shows that the intercepts of the curve decreased from 4.89 Ω cm^2 at room temperature to 3.52 Ω cm^2 at 40 ℃, verifying such a synergistic effect at elevated temperature (Supplementary Fig. 73).

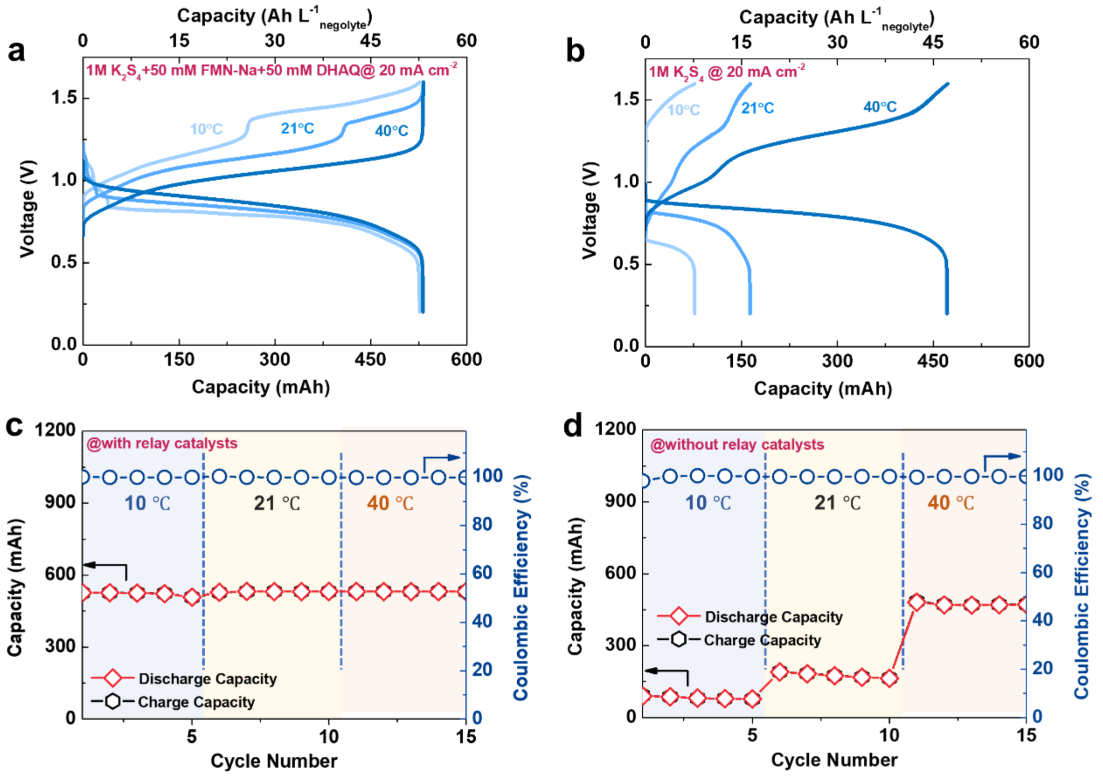

Supplementary Figure 70 | Voltage profiles of the S-Fe flow cell (a) with or (b) without relay catalysts from 10 ℃ to 40 ℃ at 20 mA cm^{-2}. The cycling stability of the S-Fe flow cell (c) with or (d) without relay catalysts from 10 ℃ to 40 ℃ (10 mL of 1 M K_2S_4 with 50 mM FMN-Na + 50 mM 2,6-DHAQ–1 M KOH |CRIS membrane| 40 mL of 0.5 M $K_4[Fe(CN)_6]$–1 M KCl).

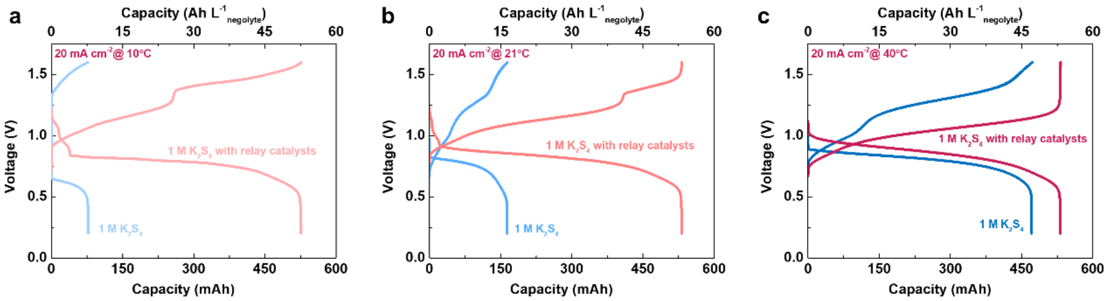

Supplementary Figure 71 | Voltage profiles of the S-Fe flow cell with or without relay catalysts at (a) 10 ℃, (b) room temperature (21 ℃), and (c) 40 ℃.

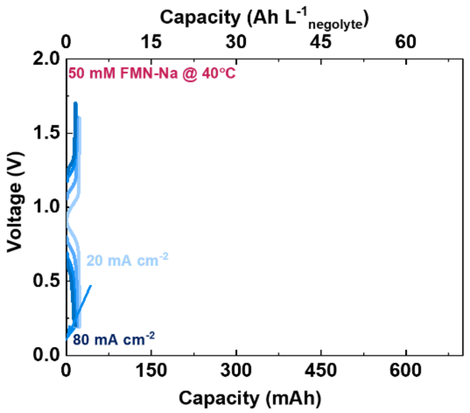

Supplementary Figure 72 | Voltage profiles of the FMN-Fe flow cell from 20 mA cm^{-2} to 80 mA cm^{-2} at 40 ℃ (10 mL of 50 mM FMN-Na–1 M KOH |CRIS membrane| 40 mL of 0.5 M $K_4[Fe(CN)_6]$–1 M KCl).

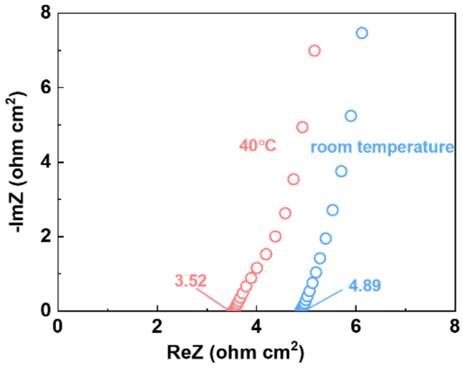

Supplementary Figure 73 | EIS Nyquist plots of S-Fe flow cell with relay catalyst at room temperature (blue curve) or 40 ℃ (red curve) (10 mL of 1 M K_2S_4 with 50 mM FMN-Na + 50 mM 2,6-DHAQ–1 M KOH |CRIS membrane| 40 mL of 0.5 M $K_4[Fe(CN)_6]$–1 M KCl).

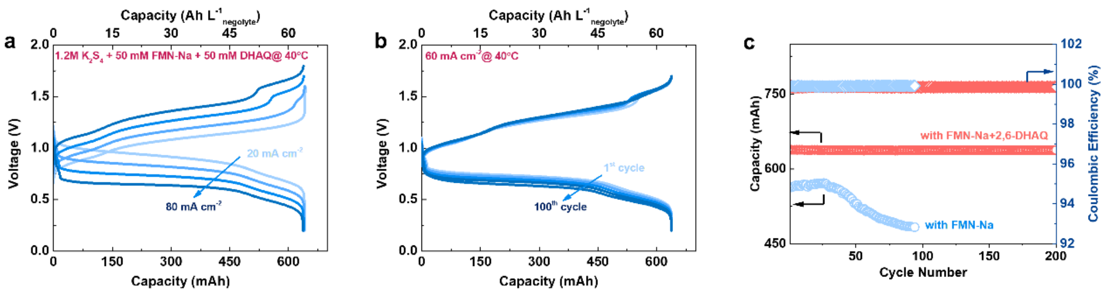

Supplementary Figure 74 | (a) Voltage profiles of the S-Fe flow cell with relay catalysts from 20 mA cm^{-2} to 80 mA cm^{-2} at 40 ℃. The (b) voltage profiles and (c) cycling stability of the S-Fe flow cell with relay catalysts (pink line) or single FMN-Na catalyst (blue line) at 60 mA cm^{-2}. (10 mL of 1.2 M K_2S_4–1 M KOH with 50 mM FMN-Na and 50 mM 2,6-DHAQ |CRIS membrane| 16 mL of 1.5 M ferrocyanide posolyte (0.75 M $K_4[Fe(CN)_6]$ + 0.75 M $Na_4[Fe(CN)_6]$). For the cell with single catalyst, only 50 mM FMN-Na is added in the negolyte side.

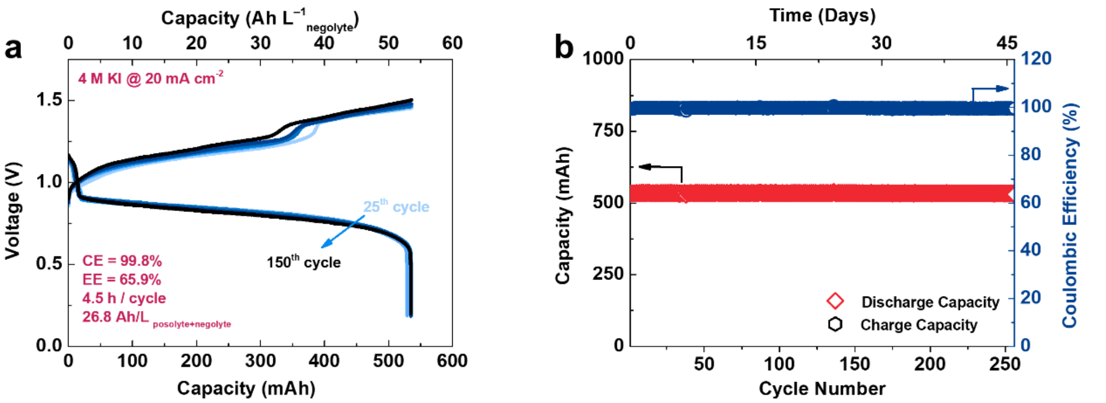

Supplementary Figure 75 | (a) The voltage profiles of the S-I flow cell. (b) The capacity and Coulombic efficiency of the S-I flow cell with relay catalysts (10 mL of 1 M K_2S_4 with 50 mM 2,6-DHAQ and 50 mM FMN-Na–1 M KOH |CRIS membrane| 10 mL of 4 M KI).

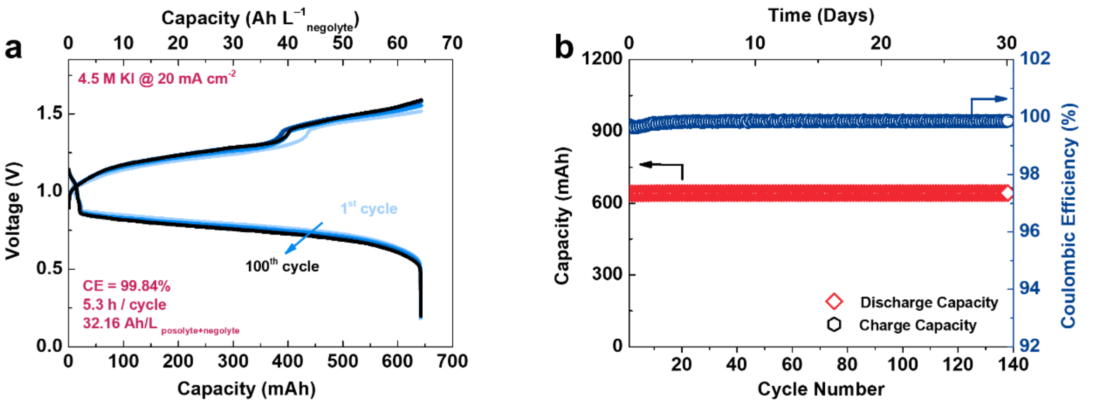

Supplementary Figure 76 | (a) The voltage profiles of the high concentration S-I flow cell. (b) The capacity and Coulombic efficiency of the S-I flow cell with relay catalysts (10 mL of 1.2 M K_2S_4 with 50 mM 2,6-DHAQ and 50 mM FMN-Na–1 M KOH |CRIS membrane| 10 mL of 4.5 M KI).

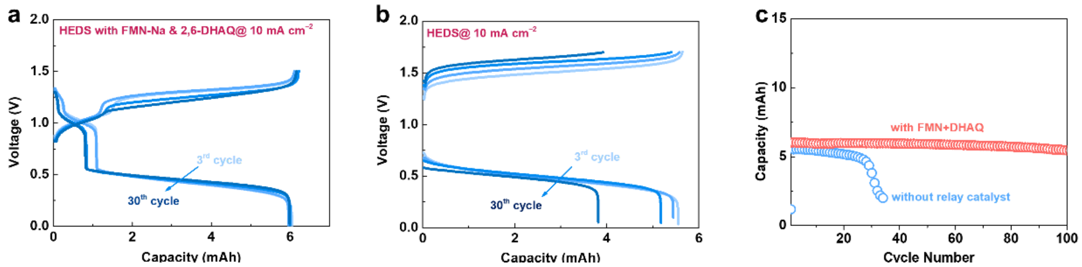

Supplementary Figure 77 | The voltage profiles of the HEDS-Fe static cell (a) with or (b) without relay catalysts. (c) The cycling stability of the HEDS-Fe cell (500 µL of 2 M HEDS with or without relay catalysts (50 mM 2,6-DHAQ and 50 mM FMN-Na)–1 M KOH | Nafion 117 membrane | 500 µL of 0.5 M $K_4[Fe(CN)_6]$–1 M KCl).

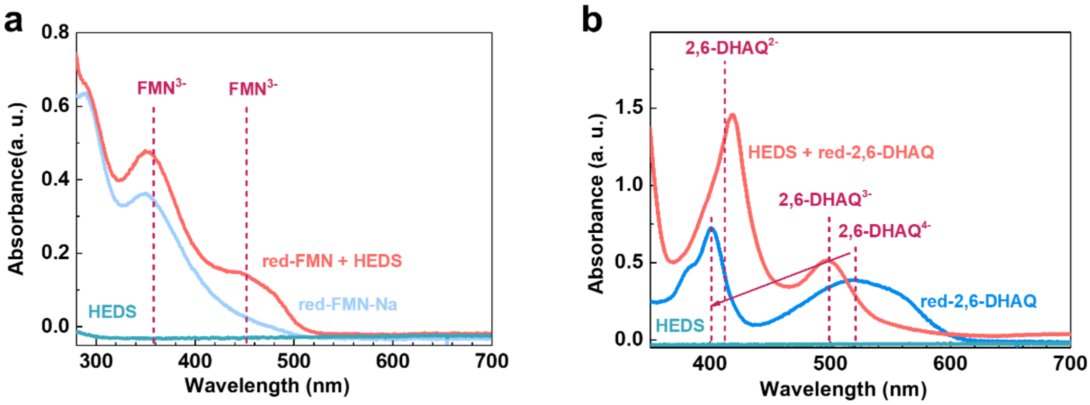

Supplementary Figure 78 | (a) The UV-vis spectra of the mixed solution of HEDS + red-FMN. (b) The UV-vis spectra of the mixed solution of HEDS + red-2,6-DHAQ.

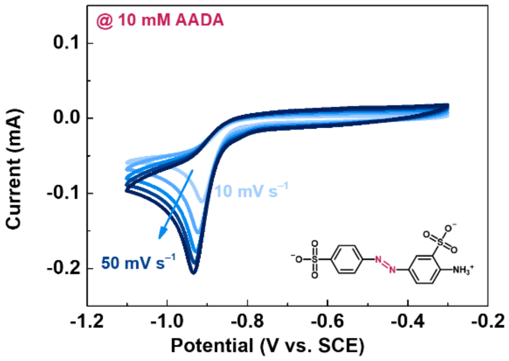

Supplementary Figure 79 | The CV plots of 10 mM AADA.

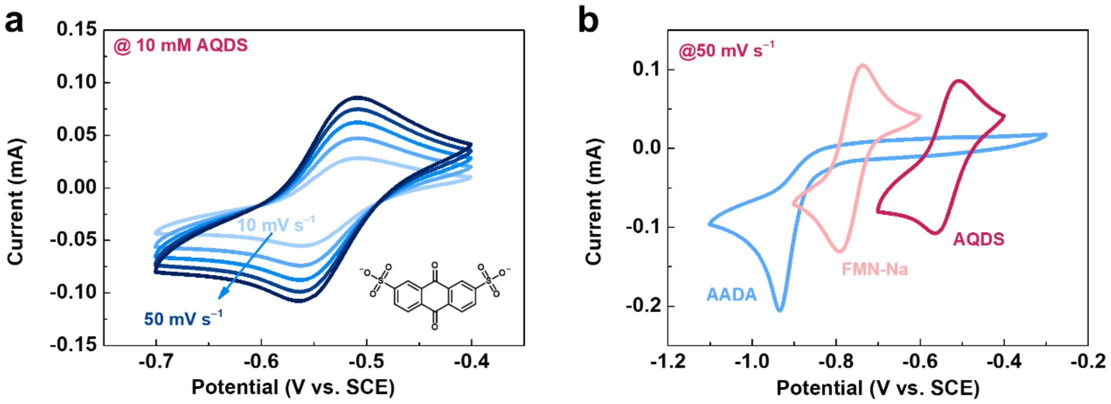

Supplementary Figure 80 | (a) The CV plots of 10 mM AQDS. (b) The CV plots of AADA, FMN-Na, and AQDS at 50 mV s^{-1}.

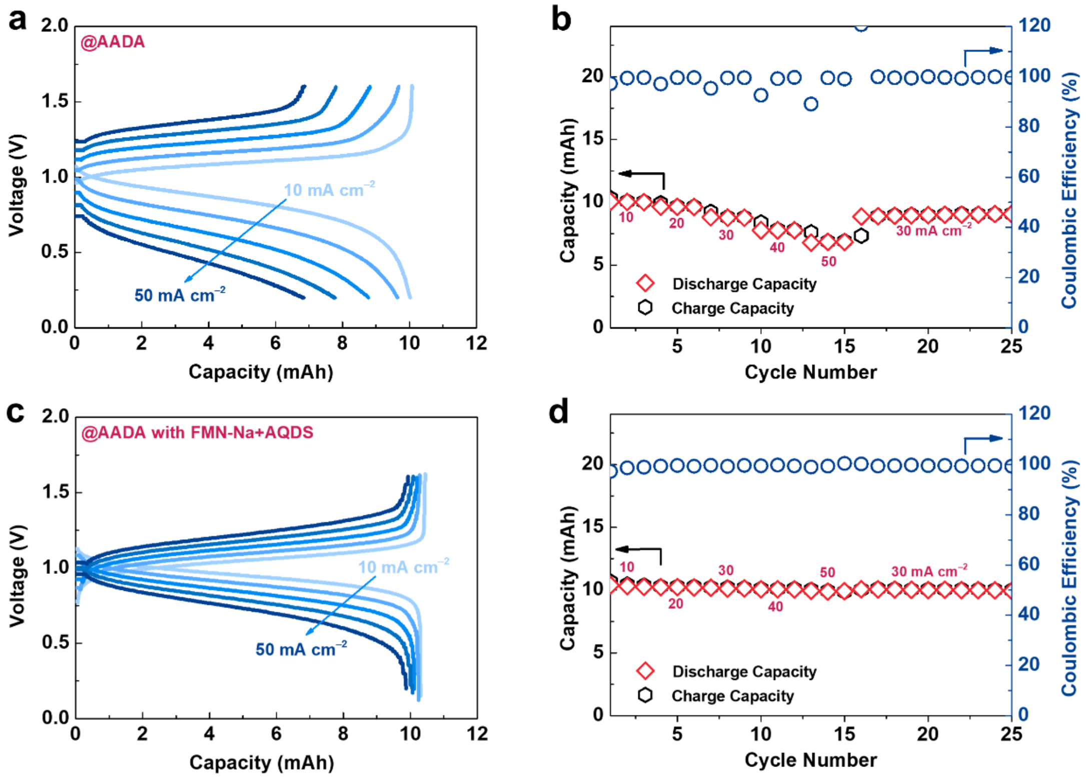

Supplementary Figure 81 | (a) The voltage profiles and (b) the rate performance of the AADA-Fe static cell (500 μL of 0.5 M AADA–1 M KOH | Nafion 117 membrane | 1000 μL of 0.5 M $K_4[Fe(CN)_6]$–1 M KCl). (c) The voltage profiles and (d) the cycling stability of the AADA-Fe static cell with relay catalysts (500 μL of 0.5 M AADA with 50 mM AQDS and 50 mM FMN-Na–1 M KOH | Nafion 117 membrane | 1000 μL of 0.5 M $K_4[Fe(CN)_6]$–1 M KCl).

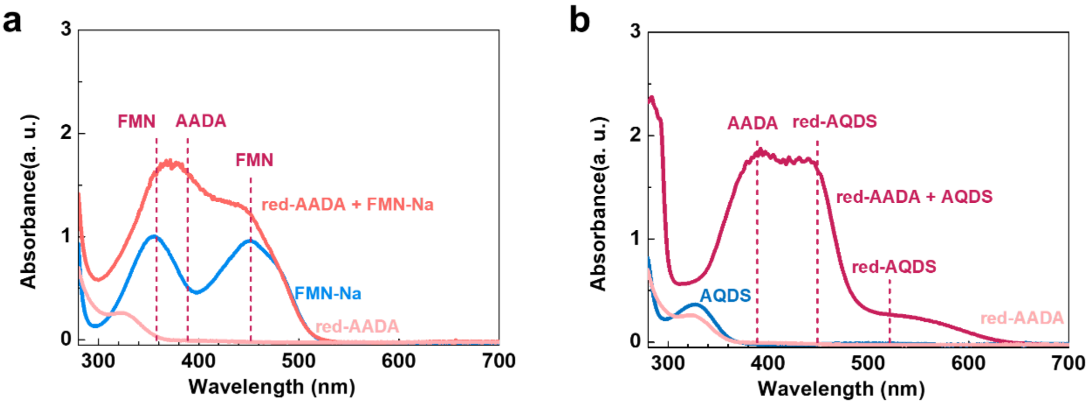

Supplementary Figure 82 | (a) The UV-vis spectra of the mixed solution of FMN-Na + red-AADA. (b) The UV-vis spectra of the mixed solution of AQDS + red-AADA.

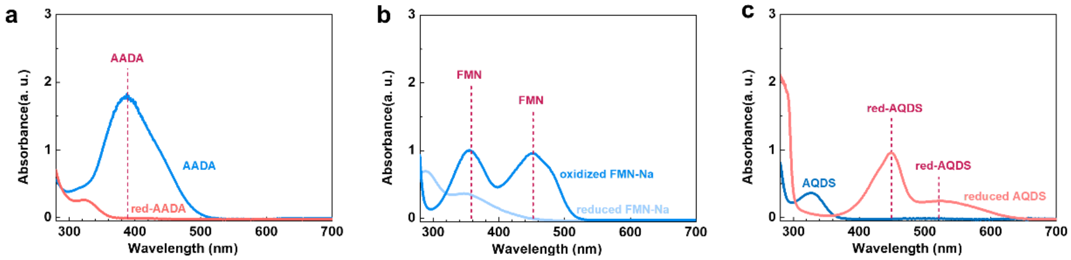

Supplementary Figure 83 | (a) The UV-vis spectra of AADA (blue line) and its reduced form (pink line). (b) The UV-vis spectra of FMN-Na (blue line) and its reduced form (light blue line). (c) The UV-vis spectra of AQDS (dark blue line) and its reduced form (light pink line).

Supplementary Note 11 | The reaction mechanism of HEDS and AADA with FMN and quinone molecular catalysts.

The S-S bond reduction reaction of organosulfides by molecular catalysts is verified by the UV-vis spectra. After mixing the reduced FMN-Na (FMN^{5-}) with HEDS, the absorbance of oxidized FMN^{3-} at 358 nm and 452 nm increases, indicating the oxidation process of FMN (Supplementary Fig. 78a). While HEDS shows almost no absorbance at the same wavelength region. For 2,6-DHAQ, the absorbance at 401 nm shifts to 419 nm, and the absorbance at 499 nm increases, suggesting the oxidation of 2,6-$DHAQ^{4-}$ and the appearance of the 2,6-$DHAQ^{3-}$ intermediate (Supplementary Fig. 78b). These results confirm the spontaneous chemical reaction between reduced relay catalysts (reduced FMN-Na and reduced 2,6-DHAQ) and HEDS.

We further investigated the reaction mechanism between reduced AADA (red-AADA) and molecular catalysts to evaluate whether red-AADA could be oxidized chemically. After mixing the red-AADA with FMN^{3-} (oxidized state), the absorbance at 389 nm increases (Supplementary Fig. 82a), attributed to the oxidation of red-AADA (Supplementary Fig. 83a). The reduction of FMN^{3-} would lead to a decrease on the absorbance at the same wavelength (Supplementary Fig. 83b) but the absorbance from oxidized AADA is dominant at the same concentration. The mixed solution of red-AADA and AQDS shows doublet at 389 nm and 449 nm due to the oxidation of red-AADA and the reduction of AQDS (Supplementary Fig. 83c). The UV-vis spectra substantiated that FMN and AQDS can chemically oxidize red-AADA while being reduced, which is followed by re-oxidation on the electrode and continuing this cycle to catalyze the oxidation reaction of red-AADA.

Supplementary Table 1 | The summary of configurations (K_2S_4-2DHAQ^{4-}), including corresponding interaction energy, angle between norm of two DHAQ planes, and distance between geometric center of two DHAQ molecules.

Configurations	Interaction energy ΔE (kcal/mol)	Ring-ring angle θ (°)	Ring-ring distance L (Å)
A1	-30.36	78.09	6.53
A2	-30.16	73.29	7.24
A3	-30.04	70.36	7.38
A4	-29.97	76.09	7.47
A5	-29.67	84.56	6.86
A6	-28.84	74.55	6.63
B1	-27.57	104.34	8.34
B2	-26.96	116.96	8.13
B3	-26.92	98.93	7.68
B4	-25.48	131.28	8.41
B5	-24.20	147.90	8.40
B6	-24.05	135.77	8.20
C1	-28.64	1.67	3.79
C2	-27.99	2.85	3.73
C3	-26.15	4.30	4.44
C4	-23.92	1.57	3.76
C5	-23.86	2.92	3.81
C6	-22.06	0.90	3.76
D1	-23.78	127.02	11.82
D2	-21.68	109.66	13.34
D3	-21.16	97.20	13.29
D4	-18.70	154.11	16.63
D5	-18.43	62.14	13.25
D6	-17.31	156.57	13.52

Supplementary Table 2 | The comparison of the cycling stability and negolyte volumetric capacity of polysulfide-based redox flow batteries.

Negolyte composition	Posolyte composition*	Catalyst and membrane	Volumetric capacity$_{negolyte}$	Volumetric capacity$_{negolyte+posolyte}$	Cycling stability	Reference
10 mL of 1.2 M K_2S_4	24 mL of 0.5M $K_4[Fe(CN)_6]$ + 0.5M $Na_4[Fe(CN)_6]$	FMN+DHAQ relay catalysts with CRIS membrane[a]	**64 Ah L^{-1}**	18.8 Ah L^{-1}	**2689 hours** (500 cycles) with EE of 74% @ 20 mA cm^{-2}	This work
10 mL of 1 M K_2S_4	10 mL of 4 M KI (75%SOC)	FMN+DHAQ relay catalysts with CRIS membrane	**53.6 Ah L^{-1}**	26.8 Ah L^{-1}	**1000 hours** (250 cycles) with EE of 66% @ 20 mA cm^{-2}	This work
10 mL of 1 M K_2S_4	10 mL of 0.5M $K_4[Fe(CN)_6]$	FMN-Na with CRIS membrane	**13 Ah L^{-1}**	6.5 Ah L^{-1}	**1200 hours** (2000 cycles) with EE of 61% @ 40 mA cm^{-2}	5
10 mL of 1 M K_2S_4	10 mL of 2M KI (50%SOC)	FMN-Na with CRIS membrane	**17.8 Ah L^{-1}**	8.9 Ah L^{-1}	**960 hours** (1300 cycles) with EE of 59% @ 40 mA cm^{-2}	5
10 mL of 0.1 M Na_2S_2	50 mL of 1 M NaOH with O_2	Ni-foam[c] with N117+FAA-3-PK-130 membrane[b]	**2.68 Ah L^{-1}**	0.45 Ah L^{-1}	**450 hours** (80 cycles) with EE of 40% @ 1 mA cm^{-2}	7
5 mL of 2 M Na_2S_2	5 mL of 1.5 M $NaI_{1.5}$ (50%SOC)	CoS_2/CoS with N117+N115 membrane	**13 Ah L^{-1}**	6.5 Ah L^{-1}	**270 hours** (60 cycles) with EE of ~70% @ 20 mA cm^{-2}	8
10 mL of 2 M K_2S_2	10 mL of 4 M KI (50%SOC)	Ni foam[c] with CRIS membrane	**35.7 Ah L^{-1}**	17.8 Ah L^{-1}	**2200 hours** (500 cycles) with EE of 60% @ 10 mA cm^{-2}	9
5 mL of 0.5 M Na_2S_4	5 mL of 1.5 M NaI (50%SOC)	CoS_2 with N117 membrane	**13 Ah L^{-1}**	6.5 Ah L^{-1}	**530 hours** (200 cycles, refresh electrolyte 3 times) with EE of 55% @ 20 mA cm^{-2}	10
10 mL of 1 M Li_2S_4	10 mL of 0.5 M Li_2SO_4	Ni mesh[c] with LiSICON membrane	–	–	**960 hours** (40 cycles) with EE of 42% @ 0.325 mA cm^{-2}	11
40 mL of 2 M K_2S	25 mL of 0.8M $K_4[Fe(CN)_6]$	No catalyst with N212 membrane	**13 Ah L^{-1}**	8 Ah L^{-1}	**280 hours** (70 cycles) with EE of 80% @ 20 mA cm^{-2}	12

60 mL of 1 M Na_2S_2	40 mL of 1M $K_3[Fe(CN)_6]$ (70%SOC)	Co-GF with N117 membrane	**12 Ah L^{-1}**	7.2 Ah L^{-1}	**750 hours** (100 cycles) with EE of 75% @ 20 mA cm^{-2}	13
10 mL of 1 M K_2S_4	10 mL of 3 M KI (47%SOC)	Cu_7S_4/CNT with N115 membrane	**25 Ah L^{-1}**	12.5 Ah L^{-1}	**500 hours** (31 cycles) with EE of 65% @ 30 mA cm^{-2}	14
10 mL of 2 M K_2S_2	10 mL of 1.5 M $Mn(Ac)_2$ (25%SOC)	Ni foam[c] with CRIS membrane	**20 Ah L^{-1}**	10 Ah L^{-1}	**720 hours** (75 cycles) with EE of 61% @ 10 mA cm^{-2}	15
5 mL of 2 M Na_2S_2	5 mL of 2 M $NaI_{1.5}$ (25%SOC)	Cu_7S_4 with N117+N115 membrane	**13 Ah L^{-1}**	6.5 Ah L^{-1}	**280 hours** (60 cycles) with EE of 75% @ 20 mA cm^{-2}	16
10 mL of 1 M Na_2S_x	6 mL of 0.5 M $Na_4[Fe(CN)_6]$	Co and N doped carbon with N115 membrane	**8 Ah L^{-1}**	5 Ah L^{-1}	**180 hours** (300 cycles) with EE of 60% @ 60 mA cm^{-2}	17
5 mL of 2 M Na_2S_2	5 mL of 2 M $NaI_{1.5}$ (25%SOC)	Cu_2CoGeS_4 with N117+N115 membrane	**13 Ah L^{-1}**	6.5 Ah L^{-1}	**280 hours** (60 cycles) with EE of 70% @ 20 mA cm^{-2}	18
45 mL of 2 M Na_2S_2	45 mL of 1 M $NaMnO_4$	Ni foam[c] with N212 membrane	**24 Ah L^{-1}**	12 Ah L^{-1}	**227 hours** (100 cycles) with EE of 78% @ 20 mA cm^{-2}	19

* The S-Fe flow cell cycled with a voltage-cut mode, while S-O_2, S-I, and S-Mn flow cells cycled with a capacity-cut (SOC control) mode.

a. CRIS membrane is the abbreviation of charge-reinforced ion-selective membrane.

b. In this work, two membrane are adopted to build a three-chamber system, in which Nafion 117 (N117) is the cation-exchange membrane and Fumasep FAA-3-PK-130 are adopted as anion-exchange membrane.

c. Ni-foam and Ni mesh are pretreated with sulfide solution to form NiS_x on the surface as catalyst

Supplementary Table 3 | Flow rate comparison of S-Fe flow cell stack and other representative flow battery systems

	Flow rate ($mL\ min^{-1}$)	Cell size	Flow rate/ Active area ($mL\ min^{-1}\ cm^{-2}$)	Reference
S-Fe flow cell stack	200	100 cm^2 * 2	1	This work
S-Fe flow cell	50	12 cm^2	4.17	
S-Fe flow cell	50	12 cm^2	4.17	5
S-I flow cell	10	1.69 cm^2	5.92	8
S-O_2 flow cell	50	5 cm^2	10	7
S-Mn flow cell	50	12 cm^2	4.17	15
Zn-I_2 flow cell	60	5 cm^2	12	20
Zn-Mn flow cell	50	4 cm^2	12.5	21
Viologen-Fe flow cell	40	5 cm^2	8	22
Quinone-Fe flow cell	40	7 cm^2	5.71	23
All-vanadium flow cell	37.5-300	25 cm^2	1.5-12	24
All-vanadium flow cell stack	-	2714 cm^2	1	25

Supplementary References

1 Lee, K. J., Elgrishi, N., Kandemir, B. & Dempsey, J. L. Electrochemical and spectroscopic methods for evaluating molecular electrocatalysts. *Nat. Rev. Chem.* **1**, 0039, (2017).

2 Helm, M. L., Stewart, M. P., Bullock, R. M., DuBois, M. R. & DuBois, D. L. A Synthetic Nickel Electrocatalyst with a Turnover Frequency Above 100,000 s^{-1} for H_2 Production. *Science* **333**, 863-866, (2011).

3 Schöfberger, W. *et al.* A Bifunctional Electrocatalyst for Oxygen Evolution and Oxygen Reduction Reactions in Water. *Angew. Chem. Int. Ed.* **55**, 2350-2355, (2016).

4 Orita, A., Verde, M. G., Sakai, M. & Meng, Y. S. A biomimetic redox flow battery based on flavin mononucleotide. *Nat. Commun.* **7**, 13230, (2016).

5 Lei, J. *et al.* An active and durable molecular catalyst for aqueous polysulfide-based redox flow batteries. *Nat. Energy* **8**, 1355-1364, (2023).

6 Heelis, P. The photophysical and photochemical properties of flavins (isoalloxazines). *Chem. Soc. Rev.* **11**, 15-39, (1982).

7 Xia, Y. *et al.* A cost-effective alkaline polysulfide-air redox flow battery enabled by a dual-membrane cell architecture. *Nat. Commun.* **13**, 2388, (2022).

8 Ma, D. *et al.* Highly active nanostructured CoS_2/CoS heterojunction electrocatalysts for aqueous polysulfide/iodide redox flow batteries. *Nat. Commun.* **10**, 3367, (2019).

9 Li, Z. & Lu, Y.-C. Polysulfide-based redox flow batteries with long life and low levelized cost enabled by charge-reinforced ion-selective membranes. *Nat. Energy* **6**, 517-528, (2021).

10 Su, L., Badel, A. F., Cao, C., Hinricher, J. J. & Brushett, F. R. Toward an Inexpensive Aqueous Polysulfide–Polyiodide Redox Flow Battery. *Ind. Eng. Chem. Res.* **56**, 9783-9792, (2017).

11 Li, Z. *et al.* Air-Breathing Aqueous Sulfur Flow Battery for Ultralow-Cost Long-Duration Electrical Storage. *Joule* **1**, 306-327, (2017).

12 Long, Y. *et al.* A neutral polysulfide/ferricyanide redox flow battery. *iScience* **24**, 103157, (2021).

13 Wei, X. *et al.* An aqueous redox flow battery based on neutral alkali metal ferri/ferrocyanide and polysulfide electrolytes. *J. Electrochem. Soc.* **163**, A5150, (2015).

14 Qin, Y., Li, X., Liu, W. & Lei, X. High-performance aqueous polysulfide-iodide flow battery realized by an efficient bifunctional catalyst based on copper sulfide. *Mater. Today Energy* **21**, 100746, (2021).

15 Lei, J., Yao, Y., Huang, Y. & Lu, Y.-C. A Highly Reversible Low-Cost Aqueous Sulfur–Manganese Redox Flow Battery. *ACS Energy Lett.* **8**, 429-435, (2023).

16 Zai, J. *et al.* Sandwiched Cu_7S_4@graphite felt electrode for high performance aqueous polysulfide/iodide redox flow batteries: Enhanced cycling stability and electrocatalytic dynamics of polysulfides. *Mater. Chem. Phys.* **250**, 123143, (2020).

17 Lan, J. *et al.* Hierarchical Nano-Electrocatalytic Reactor for High Performance Polysulfides Redox Flow Batteries. *ACS Nano* **17**, 20492-20501, (2023).

18 Zhu, Y. *et al.* Cu_2CoGeS_4 nanocrystals for high performance aqueous polysulfide/iodide redox flow batteries: enhanced selectively towards the electrocatalytic conversion of polysulfides. *Sustain. Energy Fuels* **4**, 2892-2899, (2020).

19 Ding, M. *et al.* A Stable and Energy-Dense Polysulfide/Permanganate Flow Battery. *ACS Nano* **17**, 16252-16263, (2023).

20 Wang, C. *et al.* High-voltage and dendrite-free zinc-iodine flow battery. *Nat. Commun.* **15**, 6234, (2024).

21 Lei, J., Yao, Y., Wang, Z. & Lu, Y.-C. Towards high-areal-capacity aqueous zinc–manganese batteries: promoting MnO_2 dissolution by redox mediators. *Energy Environ. Sci.* **14**, 4418-4426, (2021).

22 Li, X. *et al.* Symmetry-breaking design of an organic iron complex catholyte for a long cyclability aqueous organic redox flow battery. *Nat. Energy* **6**, 873-881, (2021).

23 Wang, A. *et al.* Selective ion transport through hydrated micropores in polymer membranes. *Nature* **635**, 353-358, (2024).

24 Monteiro, R., Leirós, J., Boaventura, M. & Mendes, A. Insights into all-vanadium redox flow battery: A case study on components and operational conditions. *Electrochim. Acta* **267**, 80-93, (2018).

25 Park, D.-J., Jeon, K.-S., Ryu, C.-H. & Hwang, G.-J. Performance of the all-vanadium redox flow battery stack. *J. Ind. Eng. Chem.* **45**, 387-390, (2017).
