# Supplementary material for: Bio-inspired relay catalysis for aqueous redox flow batteries
Source: Nat Commun. 2026 May 27;17:6882. doi: 10.1038/s41467-026-73670-4 (PMC13389366; doi:10.1038/s41467-026-73670-4)
Supplement: Supplementary file 2 — Transparent Peer Review file [file 41467_2026_73670_MOESM2_ESM.pdf]

nature portfolio

Peer Review File

Bio-inspired relay catalysis for aqueous redox flow batteries

Corresponding Author: Professor Yi-Chun Lu

This file contains all reviewer reports in order by version, followed by all author rebuttals in order by version.

Version 0:

Reviewer comments:

Reviewer #1

(Remarks to the Author)
In this paper, Lei et al. reported a universal relay-catalysis strategy for aqueous redox flow batteries (RFBs) to achieve high volumetric capacity with minimum energy loss. Inspired by the natural respiratory chain, the author proposed isoalloxazine-quinone relay catalysts to catalyze polysulfide reduction reactions, delivering a high volumetric capacity of 64 AhL-1 with long-term stability for over 100 days (500 cycles). The reaction mechanism is well studied by operando UV-vis spectrum and DFT calculations. The high energy density of 21Wh L-1 based on both sides for S-Fe RFB at high temperature is impressive and the demonstration of flow battery stack all suggests this technology has great potential in practical applications. This work introduces a novel quinone-based catalyst for polysulfide reduction reactions, and the proposed relay catalysis strategy brings insights to the design of homogeneous catalysis for redox flow batteries. I suggest the work to be published in Nature Communications after minor revision.

Comments:
1. The main idea of relay strategy is reasonable and interesting by coupling multiple molecular catalysts with matched kinetics and potential, as demonstrated in polysulfide-based RFB with FMN-Na and 2,6-DHAQ as an example. Could this strategy be further applied to other catalyst couples to verify its universality (e.g., quinone-quinone couple for polysulfide reduction reactions)?
2. In Fig. 4, the relay catalysts show a higher capacity utilization and energy efficiency, compared with a single catalyst like FMN-Na or 2,6-DHAQ alone. However, an additional experiment of controlling the catalyst concentration at the same level (50 mM) is suggested to exclude the concentration effect (e.g., 25 mM FMN-Na + 25 mM 2,6-DHAQ).
3. It is meaningful to study the temperature effect on the catalysis process, while the capacity contribution from FMN-Na itself is unclear at various current densities and temperatures, which should be provided for showing the effectiveness of relay mechanism.
4. Some advanced Fe-S redox flow batteries should be cited in main text. (please refer to iScience, 2021, 24, 103157. Energy Mater. Adv., 2022, 9865618. J. Power Sources, 2024, 591, 233856. ChemSusChem, 2023, 16, e202300710.Advanced Energy Materials, 2025, e02374. Rare Met., 2024, 43, 5039-5047)
5. The author claimed that the membrane resistance is decreased at high temperature. However, no evidence and related data are provided to quantify this contribution.
6. The supplementary Table 2 summarized various polysulfide-based RFB systems.The authors should compared to S-permanganate RFB(ACS Nano, 2023, 17, 16, 16252-16263) and it is also suggested to include the catalyst and membrane for better comparison.

Reviewer #2

(Remarks to the Author)
This manuscript reports a bio-inspired relay catalysis strategy for aqueous redox flow batteries (RFBs), in which two homogeneous molecular catalysts with distinct redox potentials and kinetic properties operate sequentially to overcome the long-standing trade-off between overpotential and reaction rate. Inspired by biological electron-transfer chains, the authors demonstrate that a low-overpotential but slower catalyst (FMN-Na) initiates the reaction at high reactant concentration, while a higher-activity, lower-potential catalyst (anthraquinone derivatives, particularly 2,6-DHAQ) takes over at higher state of charge.

The most noteworthy results include the following points:

1. Nearly 100% polysulfide utilization with a decent volumetric capacity up to ~64 Ah L^{-1} (negolyte).

2. High energy efficiency maintained despite full capacity utilization.

3. Excellent long-term stability (>500 cycles, multi-month operation).

4. Operando UV–vis spectroscopy directly visualizing the relay transition between catalysts.

Significance and relation to existing literature

The work is highly significant for the aqueous RFB field and more broadly for electrochemical energy storage using earth-abundant redox couples. Polysulfide-based and organic aqueous RFBs are widely recognized as cost-effective alternatives to vanadium systems, but sluggish kinetics and poor capacity utilization have severely limited their practical relevance. This manuscript addresses that core bottleneck in a conceptually new way.

Compared to established literature:

Previous homogeneous catalysis or redox-mediated strategies typically rely on single mediators, which inherently face a trade-off between kinetics and overpotential.

Dual-catalyst systems reported previously usually assign catalysts to separate charge/discharge roles, rather than sequential activation during a single half-reaction. The present work distinguishes itself by demonstrating a concentration- and potential-triggered relay process, supported by direct operando evidence, though I think it's still based on a straightforward extension of single mediator concept.

A few minor points could improve clarity but do not prohibit publication:

The distinction between “relay catalysis” and other multi-catalyst or mediator approaches could be stated more explicitly to avoid conceptual ambiguity for non-specialist readers ; Quantitative comparison with representative state-of-the-art polysulfide or organic aqueous RFB systems would further contextualize the performance gains ; Minor inconsistencies in notation of catalyst redox states across figures and text should be checked.

These issues are minor and can be addressed through revision without altering the main conclusions.

Reviewer #3

(Remarks to the Author)
The authors show a very interesting bioinspired approach for increasing the kinetics of sluggish flow battery materials by employing redox molecules with facile kinetics as homogenous catalysts to enhance the performance of polysulfide flow batteries. Previous work from the same group utilized a single mediator (ref. 18), enhancing the polysulfide utilization only slightly. In this work they adopt another mediator at lower potential to access almost 100 % utilization of polysulfide, resulting in unpreceded charge storage densities for such an inexpensive chemistry. This approach is shown to be applicable to other chemistries as well. Much of the focus of the work is on UV/Vis measurements to illustrate how the system operates. I appreciate this idea, and must congratulate the authors on well-done experimental work. As a minor point, the flow of the text should be improved as discussed below. Therefore I feel that the work is publishable in this journal.

However, the COMSOL simulations do not make any sense, as the simulation is not representative of the flow battery conditions, so they only serve to confuse the readers. Either the simulations have to be done properly, or they should be completely left out and explored separately. There is enough content in the paper as it is without the simulations.

Some points to be addressed:
It is claimed that energy efficiencies of batteries reported in the literature remain at 50-60 %. Energy efficiencies of >80% have been demonstrated in literature, albeit at low current density of 20 mA/cm2. https://doi.org/10.1016/j.isci.2021.103157

It is not easy to follow how the experiments were done. In UV/Vis investigations, please refer to relevant sections of the experimental part. For operando UV/Vis, what was the current density for battery testing?

For four-electrode test, do you mean that a reference electrode was included in both tanks? Please show the actual values of the measured potential differences in Fig. S42. Also please check the caption, as there concentration of K2S4 is mentioned as 250 mM or 1 M. The claim that main energy efficiency loss in this system is from the high resistance of Nafion 117 membrane is not well justified. The SCE-SCE potential allows directly measuring the Ohmic drop, so please use this value to evaluate what is the contribution of the membrane to the energy loss.

What is the energy efficiency of the 100 cm2 stack, and how does it compare to smaller cell?

How are the capacity fade rates for the larger cell, and at different temperatures.

It is stated that carbon felt was used as a current collector, but this is incorrect, as this is an electrode, while carbon plate is the current collector. What kind of pump and tubing were used, and what was the gasket material? How is the electrolyte introduced into the electrode, i.e. is there a flow channel in the carbon plate? Would be nice to have photos of the cell design in the supplementary material?

Eq. S10 is not properly balanced (there should be 2 S22-)

COMSOL simulations
The COMSOL simulations are very unrealistic, so they should be completely removed or done properly with a 2D electrode, as COMSOL example for vanadium. At the moment it does not bring any added value to the work, and I feel that the simulation could be a follow-up work. In the current simulation the electrode potential varies along the electrode, mass transport does not represent the experimental flow cell work, and kinetic expressions are unrealistic.

For COMSOL simulations, negative half-cell and tank should be considered, but for simplicity, membrane and the positive side could be ignored and membrane-electrode interface can be just considered a source or sink of K+. The tank should be considered as homogenous reactions between polysulfide and mediator can also take place in the tank. Here, the tank could be assumed as ideally stirred reactor. The convection should also be modelled in the cell.

Nernst-Planck equations are valid only for dilute electrolytes, and correct treatment of such complicated electrolytes is very challenging, so this could be mentioned.

As I understand the set-up, the felt electrode is pressed against the carbon plate, so why would there be a diffusion layer in the COMSOL model? Or is there a flow channel in the plate? Why is the thickness of the diffusion layer 50 um and electrode 100 um? It seems that the geometry was adopted from ref. 8, but this does not make any sense as there the electrode was on the bottom, and it was approached with a microelectrode from the solution, without any convection.

Why the catalytic processes (S18 and S19) are considered irreversible? The correct way would be to derive an equilibrium constant from equation S5 and then evaluate the forward and backward reactions from equilibrium constant and UV/Vis data, as K = kf/kb (or other way depending on how K is defined).

Why take only cathodic Tafel equation and not consider the full Butler-Volmer? This is incorrect, as the potential range is such that both the anodic and cathodic branch should be considered. Also polysulfide reactions at the electrode are not considered.

The boundary conditions and domain equation for current flow required for Nernst-Planck equation are not given.

Reviewer #4

(Remarks to the Author)
The authors have designed relay-catalysts to improve the capacity and stability of polysulfide-ferrocyanide-based RFBs. It is comprehsive works which includes mechanistic study, molecular modelling, electrochemical characterization, and multi-physics simulations. It suits Nat. Com. well. Nevertheless, I have the following two points that should be clarified by the authors during the revisions.

1) For aqueous RFBs, the energy density is usually limited by the solubility. How does the reply catalysis change the solubility of polysulfide-ferrocyanide?

2) I understand that the system with FMN-Na and 2,6-DHAQ can last over 500 cycles. But does this depend on the concentration of FMN-Na and 2,6-DHAQ? In other words, how stable are these relay-catalysts? What can happen is that a high concentration of relay-catalysts masks their instability, so the RFBs can last as long as these relay-catalysts are not yet decomposed.

3) For the COMSOL simulation, there is no list of parameters for the reaction-diffusion flow. This makes the work unreproducible. Therefore, a table that lists all physical parameters used in the COMSOL simulations should be provided in SI.

Version 1:

Reviewer comments:

Reviewer #1

(Remarks to the Author)
The authors have adequately addressed the comments by this reviewer and significantly improved the manuscript. I recommend to accept this work at its present form.

Reviewer #2

(Remarks to the Author)

I am satisfied with the revised manuscript and recommend its acceptance in the current form

Reviewer #3

(Remarks to the Author)
The authors have adressed all the comments by the reviewers in a satisfactory manner, and I beleieve that the work can be accepted for publication.

Reviewer #4

(Remarks to the Author)
The authors have revised the manuscript sufficiently in response to the referees' comments. The computational aspects (DFT calculations) are also robust. Therefore, I recommend it for publication.

Open Access This Peer Review File is licensed under a Creative Commons Attribution 4.0 International License, which permits use, sharing, adaptation, distribution and reproduction in any medium or format, as long as you give appropriate credit to the original author(s) and the source, provide a link to the Creative Commons license, and indicate if changes were made.
In cases where reviewers are anonymous, credit should be given to 'Anonymous Referee' and the source.
The images or other third party material in this Peer Review File are included in the article’s Creative Commons license, unless indicated otherwise in a credit line to the material. If material is not included in the article’s Creative Commons license and your intended use is not permitted by statutory regulation or exceeds the permitted use, you will need to obtain permission directly from the copyright holder.
To view a copy of this license, visit https://creativecommons.org/licenses/by/4.0/

Here we address the comments of Reviewer #1 in detail:

In this paper, Lei et al. reported a universal relay-catalysis strategy for aqueous redox flow batteries (RFBs) to achieve high volumetric capacity with minimum energy loss. Inspired by the natural respiratory chain, the author proposed isoalloxazine-quinone relay catalysts to catalyze polysulfide reduction reactions, delivering a high volumetric capacity of 64 AhL-1 with long-term stability for over 100 days (500 cycles). The reaction mechanism is well studied by operando UV-vis spectrum and DFT calculations. The high energy density of 21Wh L-1 based on both sides for S-Fe RFB at high temperature is impressive and the demonstration of flow battery stack all suggests this technology has great potential in practical applications. This work introduces a novel quinone-based catalyst for polysulfide reduction reactions, and the proposed relay catalysis strategy brings insights to the design of homogeneous catalysis for redox flow batteries. I suggest the work to be published in Nature Communications after minor revision.

Author reply: We deeply thank the reviewer's encouraging comments and insightful suggestions for us to improve our work.

Reviewer comments (1/6): The main idea of relay strategy is reasonable and interesting by coupling multiple molecular catalysts with matched kinetics and potential, as demonstrated in polysulfide-based RFB with FMN-Na and 2,6-DHAQ as an example. Could this strategy be further applied to other catalyst couples to verify its universality (e.g., quinone-quinone couple for polysulfide reduction reactions)?

Author reply: We thank the reviewer's insightful comments, which allow us to clarify and improve.

We fully agree with the reviewer that this universal strategy could be widely applied to various relay catalyst couples. Here, we applied 1,4-DHAQ (–0.53 V vs. SHE) and 1,8-DHAQ (–0.56 V vs. SHE) to replace FMN-Na and couple them with 2,6-DHAQ to show its universality. Furthermore, we explored 7,8-dihydroxyphenazine-2-sulfonic acid (DHPS) as the high-rate catalyst. The voltage profiles at 20 mA cm^{-2} demonstrate that both quinone-quinone and quinone-phenazine couples greatly boost the achieved capacity with a low overpotential, verifying that such a relay mechanism could be widely applied to various molecular catalyst couples.

We revised the manuscript on page 14 to clarify:

This relay strategy was further applied to other catalyst-couples with matched kinetics and potential, like quinone-quinone and quinone-phenazine relay catalysts. Supplementary Fig. 55 shows that by coupling high-potential 1,4-DHAQ and 1,8-DHAQ with high-rate 2,6-DHAQ or 7,8-dihydroxyphenazine-2-sulfonic acid (DHPS), the S-Fe flow cells all achieve a high capacity utilization with low overpotential in three different relay couples (1,4-DHAQ + 2,6-DHAQ, 1,4-DHAQ + 2,6-DHAQ, and 1,4-DHAQ + DHPS), showing its wide universality.

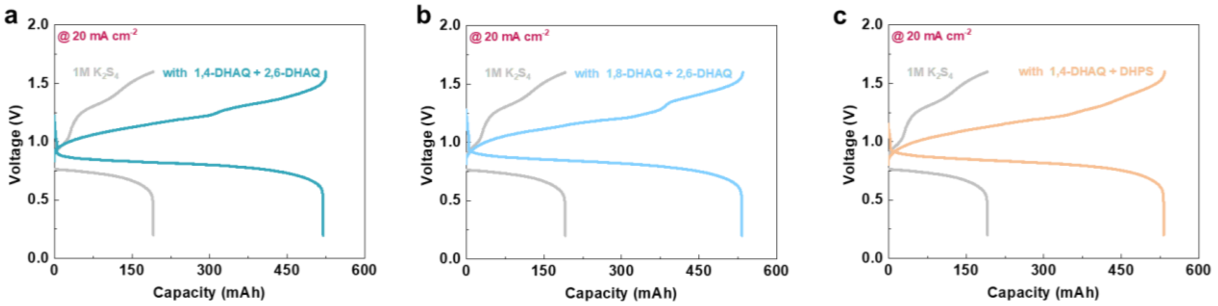

Supplementary Figure 55 | (a) The voltage profiles of S-Fe flow cells with or without relay catalysts at 20 mA cm^{-2} (10 ml of 1 M K_2S_4 with or without 10 mM 1,4-DHAQ and 10 mM 2,6-DHAQ–1 M KOH |CRIS membrane| 40 ml of 0.5 M $K_4[Fe(CN)_6]$–1 M KCl). (b) The voltage profiles of S-Fe flow cells with or without relay catalysts at 20 mA cm^{-2} (10 ml of 1 M K_2S_4 with or without 10 mM 1,8-DHAQ and 10 mM 2,6-DHAQ–1 M KOH |CRIS membrane| 40 ml of 0.5 M $K_4[Fe(CN)_6]$–1 M KCl). (c) The voltage profiles of S-Fe flow cells with or without relay catalysts at 20 mA cm^{-2} (10 ml of 1 M K_2S_4 with or without 10 mM 1,4-DHAQ and 10 mM DHPS–1 M KOH |CRIS membrane| 40 ml of 0.5 M $K_4[Fe(CN)_6]$–1 M KCl).

Reviewer comments (2/6): In Fig. 4, the relay catalysts show a higher capacity utilization and energy efficiency, compared with a single catalyst like FMN-Na or 2,6-DHAQ alone. However, an additional experiment of controlling the catalyst concentration at the same level (50 mM) is suggested to exclude the concentration effect (e.g., 25 mM FMN-Na + 25 mM 2,6-DHAQ).

Author reply: We thank the reviewer's insightful comments, which allow us to clarify and improve.

We conducted additional experiments to show that the benefit of relay catalysis is more than the concentration effect. Because the concentration limit of DHAQ and FMN are all lower than 100 mM in 1 M K_2S_4 electrolyte, we compare 25 mM FMN + 25 mM DHAQ with 50 mM DHAQ and 50 mM FMN.

We revised the manuscript on page 11:

We compare 25 mM FMN + 25 mM DHAQ with 50 mM DHAQ and 50 mM FMN. As shown in Supplementary Fig. 45, the 25 mM FMN-Na + 25 mM DHAQ relay catalysts still outperform the 50 mM DHAQ or 50 mM FMN-Na on capacity and energy efficiency, supporting that the benefit of relay catalysis is more than the concentration effect.

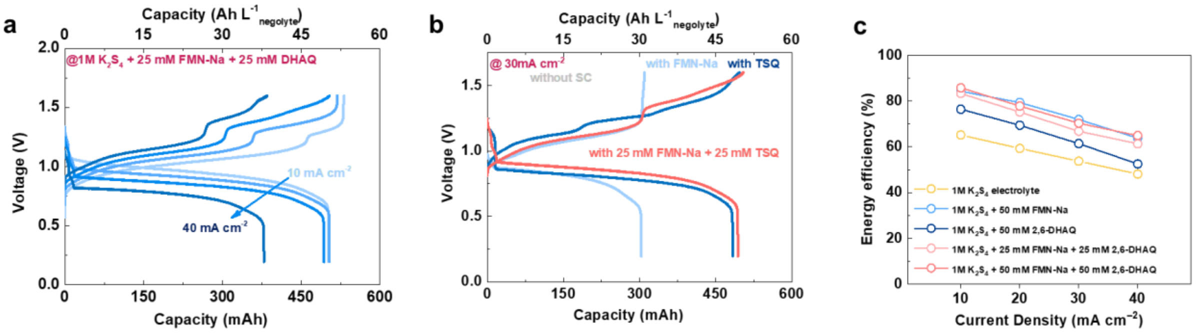

Supplementary Figure 45 | (a) The voltage profiles of the S-Fe flow cell with relay catalysts from 10 to 40 mA cm^{-2} (10 ml of 1 M K_2S_4 with 25 mM FMN-Na and 25 mM 2,6-DHAQ–1 M KOH |Nafion 117 membrane| 40 ml of 0.5 M $K_4[Fe(CN)_6]$–1 M KCl). (b) The voltage profiles of the S-Fe flow cell with 50 mM molecular catalyst at 30 mA cm^{-2}. (c) The energy efficiency comparison of the S-Fe flow cell with 50 mM molecular catalysts.

Reviewer comments (3/6): It is meaningful to study the temperature effect on the catalysis process, while the capacity contribution from FMN-Na itself is unclear at various current densities and temperatures, which should be provided for showing the effectiveness of relay mechanism.

Author reply: We thank the reviewer's insightful comments, which allow us to clarify and improve.

We conducted additional experiments to study the capacity contribution from FMN-Na itself at high current density and high temperatures, showing that the capacity contribution from FMN-Na is only around 2 Ah L^{-1}, accounting for 3% of the total capacity.

We revised the Supplementary Note 11 to clarify:

The capacity contribution from FMN-Na itself is only around 2 Ah L^{-1} which accounts for 3.7% of the total capacity, suggesting that the capacity enhancement and energy efficiency improvements are mainly from the catalysis effect (Supplementary Fig. 72).

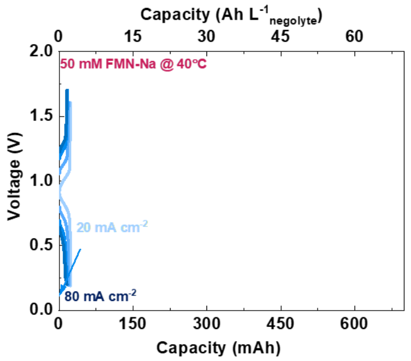

Supplementary Figure 72 | Voltage profiles of the FMN-Fe flow cell from 20 mA cm^{-2} to 80 mA cm^{-2} at 40 °C (10 ml of 50 mM FMN-Na–1 M KOH |CRIS membrane| 40 ml of 0.5 M $K_4[Fe(CN)_6]$–1 M KCl).

Reviewer comments (4/6): Some advanced Fe-S redox flow batteries should be cited in main text. (please refer to iScience, 2021, 24, 103157. Energy Mater. Adv., 2022, 9865618. J. Power Sources, 2024, 591, 233856. ChemSusChem, 2023, 16, e202300710.Advanced Energy Materials, 2025, e02374. Rare Met., 2024, 43, 5039-5047).

Author reply: We thank the reviewer's insightful comments, which allow us to clarify and improve.

We have discussed and added the relevant reference to the revised manuscript on page 2:

All-vanadium RFB is the most mature technology, however, its further employment is hindered by vanadium element scarcity, volatile high price, and toxicity[4,5]. Exploring novel RFB systems with earth-abundant elements (e.g., sulfur-based RFBs and organic-based RFBs) could substantially reduce active material cost with high sustainability[6-12]. For instance, the annual yield of sulfur is over 60,000,000 tons per year (vs. 100,000 tons per year for vanadium), while the price is 1,000 times cheaper than vanadium, suggesting they are a more sustainable choice for the growing energy storage market. Organic molecules, composed of earth-abundant elements like C, H, O, N, etc., are another potentially inexpensive alternative[13,14]. However, many competitive low-cost candidates suffer from sluggish kinetics, resulting in limited capacity utilization and low operating current density. For example, polysulfide-based RFB shows a low volumetric capacity (6–13 Ah $L^{-1}_{negolyte}$ with polysulfide utilization less than 35%) and low energy efficiency (50 – 60%)[15-17]. While utilizing polysulfide electrolyte at a low state of charge (SOC) could increase energy efficiency (80.95%), it largely limits energy density (1.6 Ah $L^{-1}_{posolyte+negolyte}$)[18]. Various organic molecules like azo compound, alloxazine, organosulfide, and fluorene molecules, are also suffering from sluggish kinetics with limited capacity utilization and high voltage gap with low operating current[19-22].

Developing effective catalytic processes and low-cost catalysts is critical for enabling the exploitation of earth-abundant, low-cost active materials in RFBs. Taking polysulfide-based RFB as an example, solid catalysts have been applied to improve the kinetics, including various metal sulfides (e.g., CoS/CoS_2, NiS_x, CuS_x, SW_2) and doped-carbon (e.g., nitrogen-doped carbon) catalysts[17,23-27]. However, their effectiveness remains unsatisfactory due to limited reaction sites, catalyst exfoliation, side reactions, and poor catalytic activity[15,19,28,29].

Reviewer comments (5/6): The author claimed that the membrane resistance is decreased at high temperature. However, no evidence and related data are provided to quantify this contribution.

Author reply: We thank the reviewer's insightful comments, which allow us to clarify and improve.

We conducted EIS tests to study the resistance change at elevated temperatures. The EIS tests indicate the intercept of the curve is decreased from 4.89 Ω cm^2 at room temperature to 3.52 Ω cm^2 at 40 °C, verifying our statements that the resistance of the flow cell decreases at elevated temperature.

We revised the Supplementary Note 11 to clarify:

The EIS spectra show that the intercepts of the curve decreased from 4.89 Ω cm^2 at room temperature to 3.52 Ω cm^2 at 40 °C, verifying such a synergistic effect at elevated temperature (Supplementary Fig. 73).

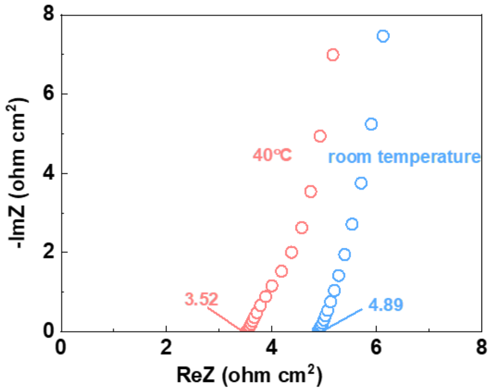

Supplementary Figure 73 | EIS tests of S-Fe flow cell with relay catalyst at room temperature (blue curve) or 40 °C (red curve) (10 ml of 1 M K_2S_4 with 50 mM FMN-Na + 50 mM 2,6-DHAQ–1 M KOH |CRIS membrane| 40 ml of 0.5 M $K_4[Fe(CN)_6]$–1 M KCl).

Reviewer comments (6/6): The supplementary Table 2 summarized various polysulfide-based RFB systems. The authors should compared to S-permanganate RFB(ACS Nano, 2023, 17, 16, 16252-16263) and it is also suggested to include the catalyst and membrane for better comparison.

Author reply: We thank the reviewer's insightful comments, which allow us to clarify and improve.

We have added the catalyst and membrane into comparison with polysulfide-based RFBs and polysulfide-permanganate RFB systems.

We revised the supplementary information to clarify:

Supplementary Table 2 | The comparison of the cycling stability and negolyte volumetric capacity of polysulfide-based redox flow batteries.

Negolyte composition	Posolyte composition*	Catalyst and membrane	Volumetric $capacity_{negolyte}$	Volumetric $capacity_{negolyte+posolyte}$	Cycling stability	Reference
10 mL of 1.2 M K_2S_4	24 mL of 0.5M $K_4[Fe(CN)_6]$ + 0.5M $Na_4[Fe(CN)_6]$	FMN+DHAQ relay catalysts with CRIS membrane[a]	**64 Ah L^{-1}**	18.8 Ah L^{-1}	**2689 hours** (500 cycles) with EE of 74% @ 20 mA cm^{-2}	This work
10 mL of 1 M K_2S_4	10 mL of 4 M KI (75%SOC)	FMN+DHAQ relay catalysts with CRIS membrane	**53.6 Ah L^{-1}**	26.8 Ah L^{-1}	**1000 hours** (250 cycles) with EE of 66% @ 20 mA cm^{-2}	This work
10 mL of 1 M K_2S_4	10 mL of 0.5M $K_4[Fe(CN)_6]$	FMN-Na with CRIS membrane	**13 Ah L^{-1}**	6.5 Ah L^{-1}	**1200 hours** (2000 cycles) with EE of 61% @ 40 mA cm^{-2}	5
10 mL of 1 M K_2S_4	10 mL of 2M KI (50%SOC)	FMN-Na with CRIS membrane	**17.8 Ah L^{-1}**	8.9 Ah L^{-1}	**960 hours** (1300 cycles) with EE of 59% @ 40 mA cm^{-2}	5
10 mL of 0.1	50 mL of 1 M NaOH with O_2	Ni-foam[c] with N117+FAA-3-	**2.68 Ah L^{-1}**	0.45 Ah L^{-1}	**450 hours** (80 cycles) with EE of 40% @ 1 mA cm^{-2}	14

M Na_2S_2		PK-130 membrane[b]				
5 mL of 2 M Na_2S_2	5 mL of 1.5 M $NaI_{1.5}$ (50%SOC)	CoS_2/CoS with N117+N115 membrane	**13 Ah L^{-1}**	6.5 Ah L^{-1}	**270 hours** (60 cycles) with EE of ~70% @ 20 mA cm^{-2}	15
10 mL of 2 M K_2S_2	10 mL of 4 M KI (50%SOC)	Ni foam[c] with CRIS membrane	**35.7 Ah L^{-1}**	17.8 Ah L^{-1}	**2200 hours** (500 cycles) with EE of 60% @ 10 mA cm^{-2}	16
5 mL of 0.5 M Na_2S_4	5 mL of 1.5 M NaI (50%SOC)	CoS_2 with N117 membrane	**13 Ah L^{-1}**	6.5 Ah L^{-1}	**530 hours** (200 cycles, refresh electrolyte 3 times) with EE of 55% @ 20 mA cm^{-2}	17
10 mL of 1 M Li_2S_4	10 mL of 0.5 M Li_2SO_4	Ni mesh[c] with LiSICON membrane	–	–	**960 hours** (40 cycles) with EE of 42% @ 0.325 mA cm^{-2}	18
40 mL of 2 M K_2S	25 mL of 0.8M $K_4[Fe(CN)_6]$	No catalyst with N212 membrane	**13 Ah L^{-1}**	8 Ah L^{-1}	**280 hours** (70 cycles) with EE of 80% @ 20 mA cm^{-2}	19
60 mL of 1 M Na_2S_2	40 mL of 1M $K_3[Fe(CN)_6]$ (70%SOC)	Co-GF with N117 membrane	**12 Ah L^{-1}**	7.2 Ah L^{-1}	**750 hours** (100 cycles) with EE of 75% @ 20 mA cm^{-2}	20
10 mL of 1 M K_2S_4	10 mL of 3 M KI (47%SOC)	Cu_7S_4/CNT with N115 membrane	**25 Ah L^{-1}**	12.5 Ah L^{-1}	**500 hours** (31 cycles) with EE of 65% @ 30 mA cm^{-2}	21
10 mL of 2 M K_2S_2	10 mL of 1.5 M $Mn(Ac)_2$ (25%SOC)	Ni foam[c] with CRIS membrane	**20 Ah L^{-1}**	10 Ah L^{-1}	**720 hours** (75 cycles) with EE of 61% @ 10 mA cm^{-2}	22
5 mL of 2 M Na_2S_2	5 mL of 2 M $NaI_{1.5}$ (25%SOC)	Cu_7S_4 with N117+N115 membrane	**13 Ah L^{-1}**	6.5 Ah L^{-1}	**280 hours** (60 cycles) with EE of 75% @ 20 mA cm^{-2}	23
10 mL of 1 M Na_2S_x	6 mL of 0.5 M $Na_4[Fe(CN)_6]$	Co and N doped carbon with N115 membrane	**8 Ah L^{-1}**	5 Ah L^{-1}	**180 hours** (300 cycles) with EE of 60% @ 60 mA cm^{-2}	24
5 mL of 2 M Na_2S_2	5 mL of 2 M $NaI_{1.5}$ (25%SOC)	Cu_2CoGeS_4 with N117+N115 membrane	**13 Ah L^{-1}**	6.5 Ah L^{-1}	**280 hours** (60 cycles) with EE of 70% @ 20 mA cm^{-2}	25
45 mL of 2 M Na_2S_2	45 mL of 1 M $NaMnO_4$	Ni foam[c] with N212 membrane	**24 Ah L^{-1}**	12 Ah L^{-1}	**227 hours** (100 cycles) with EE of 78% @ 20 mA cm^{-2}	26

* The S-Fe flow cell cycled with a voltage-cut mode, while S-O_2, S-I, and S-Mn flow cells cycled with a capacity-cut (SOC control) mode.

a. CRIS membrane is the abbreviation of charge-reinforced ion-selective membrane.

b. In this work, two membrane are adopted to build a three-chamber system, in which Nafion 117 (N117) is the cation-exchange membrane and Fumasep FAA-3-PK-130 are adopted as anion-exchange membrane.

c. Ni-foam and Ni mesh are pretreated with sulfide solution to form NiS_x on the surface as catalyst

Here we address the comments of Reviewer #2 in detail:

This manuscript reports a bio-inspired relay catalysis strategy for aqueous redox flow batteries (RFBs), in which two homogeneous molecular catalysts with distinct redox potentials and kinetic properties operate sequentially to overcome the long-standing trade-off between overpotential and reaction rate. Inspired by biological electron-transfer chains, the authors demonstrate that a low-overpotential but slower catalyst (FMN-Na) initiates the reaction at high reactant concentration, while a higher-activity, lower-potential catalyst (anthraquinone derivatives, particularly 2,6-DHAQ) takes over at higher state of charge.

The most noteworthy results include the following points:

1. Nearly 100% polysulfide utilization with a decent volumetric capacity up to ~64 Ah L^{-1} (negolyte).

2. High energy efficiency maintained despite full capacity utilization.

3. Excellent long-term stability (>500 cycles, multi-month operation).

4. Operando UV–vis spectroscopy directly visualizing the relay transition between catalysts.

Significance and relation to existing literature

The work is highly significant for the aqueous RFB field and more broadly for electrochemical energy storage using earth-abundant redox couples. Polysulfide-based and organic aqueous RFBs are widely recognized as cost-effective alternatives to vanadium systems, but sluggish kinetics and poor capacity utilization have severely limited their practical relevance. This manuscript addresses that core bottleneck in a conceptually new way.

Compared to established literature:

Previous homogeneous catalysis or redox-mediated strategies typically rely on single mediators, which inherently face a trade-off between kinetics and overpotential.

Dual-catalyst systems reported previously usually assign catalysts to separate charge/discharge roles, rather than sequential activation during a single half-reaction. The present work distinguishes itself by demonstrating a concentration- and potential-triggered relay process, supported by direct operando evidence, though I think it's still based on a straightforward extension of single mediator concept.

A few minor points could improve clarity but do not prohibit publication: The distinction between "relay catalysis" and other multi-catalyst or mediator approaches could be stated more explicitly to avoid conceptual ambiguity for non-specialist readers ; Quantitative comparison with representative state-of-the-art polysulfide or organic aqueous RFB systems would further contextualize the performance gains ; Minor inconsistencies in notation of catalyst redox states across figures and text should be checked.

Author reply: We deeply thank the reviewer's encouraging comments and insightful suggestions for us to improve our work.

Reviewer comments (1/3): The distinction between "relay catalysis" and other multi-catalyst or mediator approaches could be stated more explicitly to avoid conceptual ambiguity for non-specialist readers ;

Author reply: We thank the reviewer's insightful comments, which allow us to clarify and improve.

We revised the manuscript on page 5:

The key novelty of this approach lies in the "relay operation" in homogeneous catalysis, which should be differentiated from the commonly reported dual catalysts (or dual mediators) that each catalyst works on different reactions[51,52]. For instance, one catalyst only works on discharge reactions, and the other one works for charge reactions. In this way, the limitation and the trade-off (between kinetics and overpotential) of the single catalyst still exist in two separate reactions. However, in our relay approach, the design principle of relay catalysis on the same reaction lies in the potential/kinetics match and the dynamic transition from the low-rate catalyst to a high-rate catalyst via concentration-triggered potential shift. This cooperative relay mechanism effectively breaks the trade-off between decreasing overpotential and increasing driving force via streamlining the electron transfer sequence of multiple catalysts, maximizing achieved capacity while minimizing energy loss.

Reviewer comments (2/3): Quantitative comparison with representative state-of-the-art polysulfide or organic aqueous RFB systems would further contextualize the performance gains;

Author reply: We thank the reviewer's insightful comments, which allow us to clarify and improve.

We compare the volumetric capacity and cycling life of the state-of-the-art polysulfide-based redox flow batteries, and our relay catalyst-assisted S-Fe RFB and S-I RFB all show superior performance.

We revised the manuscript on page 14:

We summarized the achieved volumetric capacity and cycling life with the state-of-the-art polysulfide-based redox flow batteries, including S-Fe, S-O_2, S-I, and S-Mn flow cells (Supplementary Table 2), and the relay-catalysts-assisted cells shows a superior performance.

Supplementary Table 2 | The comparison of the cycling stability and negolyte volumetric capacity of polysulfide-based redox flow batteries.

Negolyte composition	Posolyte composition*	Catalyst and membrane	Volumetric capacity$_{negolyte}$	Volumetric capacity$_{negolyte+posolyte}$	Cycling stability	Reference
10 mL of 1.2 M K_2S_4	24 mL of 0.5M $K_4[Fe(CN)_6]$ + 0.5M $Na_4[Fe(CN)_6]$	FMN+DHAQ relay catalysts with CRIS membrane[a]	**64 Ah L^{-1}**	18.8 Ah L^{-1}	**2689 hours** (500 cycles) with EE of 74% @ 20 mA cm^{-2}	This work
10 mL of 1 M K_2S_4	10 mL of 4 M KI (75%SOC)	FMN+DHAQ relay catalysts with CRIS membrane	**53.6 Ah L^{-1}**	26.8 Ah L^{-1}	**1000 hours** (250 cycles) with EE of 66% @ 20 mA cm^{-2}	This work
10 mL of 1 M K_2S_4	10 mL of 0.5M $K_4[Fe(CN)_6]$	FMN-Na with CRIS membrane	**13 Ah L^{-1}**	6.5 Ah L^{-1}	**1200 hours** (2000 cycles) with EE of 61% @ 40 mA cm^{-2}	5
10 mL of 1 M K_2S_4	10 mL of 2M KI (50%SOC)	FMN-Na with CRIS membrane	**17.8 Ah L^{-1}**	8.9 Ah L^{-1}	**960 hours** (1300 cycles) with EE of 59% @ 40 mA cm^{-2}	5
10 mL of 0.1 M Na_2S_2	50 mL of 1 M NaOH with O_2	Ni-foam[c] with N117+FAA-3-PK-130 membrane[b]	**2.68 Ah L^{-1}**	0.45 Ah L^{-1}	**450 hours** (80 cycles) with EE of 40% @ 1 mA cm^{-2}	14
5 mL of 2 M Na_2S_2	5 mL of 1.5 M $NaI_{1.5}$ (50%SOC)	CoS_2/CoS with N117+N115 membrane	**13 Ah L^{-1}**	6.5 Ah L^{-1}	**270 hours** (60 cycles) with EE of ~70% @ 20 mA cm^{-2}	15
10 mL of 2 M K_2S_2	10 mL of 4 M KI (50%SOC)	Ni foam[c] with CRIS membrane	**35.7 Ah L^{-1}**	17.8 Ah L^{-1}	**2200 hours** (500 cycles) with EE of 60% @ 10 mA cm^{-2}	16
5 mL of 0.5 M Na_2S_4	5 mL of 1.5 M NaI (50%SOC)	CoS_2 with N117 membrane	**13 Ah L^{-1}**	6.5 Ah L^{-1}	**530 hours** (200 cycles, refresh electrolyte 3 times) with EE of 55% @ 20 mA cm^{-2}	17
10 mL of 1 M Li_2S_4	10 mL of 0.5 M Li_2SO_4	Ni mesh[c] with LiSICON membrane	–	–	**960 hours** (40 cycles) with EE of 42% @ 0.325 mA cm^{-2}	18

40 mL of 2 M K_2S	25 mL of 0.8M $K_4[Fe(CN)_6]$	No catalyst with N212 membrane	**13 Ah L^{-1}**	8 Ah L^{-1}	**280 hours** (70 cycles) with EE of 80% @ 20 mA cm^{-2}	19
60 mL of 1 M Na_2S_2	40 mL of 1M $K_3[Fe(CN)_6]$ (70%SOC)	Co-GF with N117 membrane	**12 Ah L^{-1}**	7.2 Ah L^{-1}	**750 hours** (100 cycles) with EE of 75% @ 20 mA cm^{-2}	20
10 mL of 1 M K_2S_4	10 mL of 3 M KI (47%SOC)	Cu_7S_4/CNT with N115 membrane	**25 Ah L^{-1}**	12.5 Ah L^{-1}	**500 hours** (31 cycles) with EE of 65% @ 30 mA cm^{-2}	21
10 mL of 2 M K_2S_2	10 mL of 1.5 M $Mn(Ac)_2$ (25%SOC)	Ni foam[c] with CRIS membrane	**20 Ah L^{-1}**	10 Ah L^{-1}	**720 hours** (75 cycles) with EE of 61% @ 10 mA cm^{-2}	22
5 mL of 2 M Na_2S_2	5 mL of 2 M $NaI_{1.5}$ (25%SOC)	Cu_7S_4 with N117+N115 membrane	**13 Ah L^{-1}**	6.5 Ah L^{-1}	**280 hours** (60 cycles) with EE of 75% @ 20 mA cm^{-2}	23
10 mL of 1 M Na_2S_x	6 mL of 0.5 M $Na_4[Fe(CN)_6]$	Co and N doped carbon with N115 membrane	**8 Ah L^{-1}**	5 Ah L^{-1}	**180 hours** (300 cycles) with EE of 60% @ 60 mA cm^{-2}	24
5 mL of 2 M Na_2S_2	5 mL of 2 M $NaI_{1.5}$ (25%SOC)	Cu_2CoGeS_4 with N117+N115 membrane	**13 Ah L^{-1}**	6.5 Ah L^{-1}	**280 hours** (60 cycles) with EE of 70% @ 20 mA cm^{-2}	25
45 mL of 2 M Na_2S_2	45 mL of 1 M $NaMnO_4$	Ni foam[c] with N212 membrane	**24 Ah L^{-1}**	12 Ah L^{-1}	**227 hours** (100 cycles) with EE of 78% @ 20 mA cm^{-2}	26

* The S-Fe flow cell cycled with a voltage-cut mode, while S-O_2, S-I, and S-Mn flow cells cycled with a capacity-cut (SOC control) mode.

a. CRIS membrane is the abbreviation of charge-reinforced ion-selective membrane.

b. In this work, two membrane are adopted to build a three-chamber system, in which Nafion 117 (N117) is the cation-exchange membrane and Fumasep FAA-3-PK-130 are adopted as anion-exchange membrane.

c. Ni-foam and Ni mesh are pretreated with sulfide solution to form NiS_x on the surface as catalyst

Reviewer comments (3/3): Minor inconsistencies in notation of catalyst redox states across figures and text should be checked.

Author reply: We thank the reviewer's insightful comments, which allow us to clarify and improve. We have carefully checked the redox states of catalysts and made sure they are consistent in the figures and full text.

Here we address the comments of Reviewer #3 in detail:

The authors show a very interesting bioinspired approach for increasing the kinetics of sluggish flow battery materials by employing redox molecules with facile kinetics as homogenous catalysts to enhance the performance of polysulfide flow batteries. Previous work from the same group utilized a single mediator (ref. 18), enhancing the polysulfide utilization only slightly. In this work they adopt another mediator at lower potential to access almost 100 % utilization of polysulfide, resulting in unpreceded charge storage densities for such an inexpensive chemistry. This approach is shown to be applicable to other chemistries as well. Much of the focus of the work is on UV/Vis measurements to illustrate how the system operates. I appreciate this idea, and must congratulate the authors on well-done experimental work. As a minor point, the flow of the text should be improved as discussed below. Therefore I feel that the work is publishable in this journal. However, the COMSOL simulations do not make any sense, as the simulation is not representative of the flow battery conditions, so they only serve to confuse the readers. Either the simulations have to be done properly, or they should be completely left out and explored separately. There is enough content in the paper as it is without the simulations.

Some points to be addressed:

Author reply: We deeply thank the reviewer's encouraging comments and insightful suggestions for us to improve our work.

Reviewer comments (1/8): It is claimed that energy efficiencies of batteries reported in the literature remain at 50-60 %. Energy efficiencies of >80% have been demonstrated in literature, albeit at low current density of 20 mA/cm2. https://doi.org/10.1016/j.isci.2021.103157

Author reply: We thank the reviewer's insightful comments, which allow us to clarify and improve.

For the polysulfide-based redox flow batteries, the energy efficiency depends on the state of charge (SOC) and membrane type in the absence of catalysts. At a very low SOC (< 5 Ah L^{-1}), the limited long-chain polysulfide (e.g., S_6^{2-}) in electrolyte would provide good kinetics, but the performance is poor when S_6^{2-} is fully depleted, accompanied by a quick energy efficiency drop. The low EE haa been verified by many other works that the polysulfide flow cell without catalysts always shows a low EE of 50-60 % when the volumetric capacity is larger than 13 Ah L^{-1}. (*Nat Energy* 8, 1355–1364 (**2023**); *Nat Commun* 16, 2885 (**2025**); *Nat Commun* 10, 3367 (**2019**)) In a word, it is challenging to achieve both high energy efficiency and high polysulfide utilization in polysulfide-based redox flow batteries without a catalyst.

In the cited reference (iScience, 2021, 24, 103157), the energy efficiency of 80% is achieved in a flow cell with a low volumetric capacity of 4.3 Ah $L^{-1}_{negolyte}$ at 20 mA cm^{-2} (25 mL 0.1 M ferrocyanide posolyte couple with 15 mL 2 M K_2S negolyte). Furthermore, the application of a thin membrane of Nafion 212 in this work is also beneficial for improved energy efficiency, but it sacrifices long-term stability at high concentration tests.

We revised the manuscript on page 2:

For example, polysulfide-based RFB shows a low volumetric capacity (6–13 Ah $L^{-1}_{negolyte}$ with polysulfide utilization less than 35%) and low energy efficiency (50 – 60%)[15-17]. While utilizing polysulfide electrolyte at a low state of charge (SOC) could increase energy efficiency (80.95%), it largely limits energy density (1.6 Ah $L^{-1}_{posolyte+negolyte}$)[18].

Reviewer comments (2/8): It is not easy to follow how the experiments were done. In UV/Vis investigations, please refer to relevant sections of the experimental part. For operando UV/Vis, what was the current density for battery testing?

Author reply: We thank the reviewer's insightful comments, which allow us to clarify and improve.

We have modified the experimental sections with more details and provided schematic illustrations of our experimental procedures, which have been referred to in the relevant figures in our methods section. The current density applied in operando UV-vis test is 3 mA cm^{-2} for low-concentration tests (1 mM test) and 20 mA cm^{-2} for high concentration test (5 mM and 250 mM test).

We revised the manuscript on Methods on page 21:

In-situ/operando UV-vis test: The UV–visible spectra were collected by the SEC2020 UV–visible Spectrophotometer (ALS Co., Ltd). The electrolyte for the UV-vis test was prepared with 1 M KOH as the supporting electrolyte. A thin quartz cell with an optical path length of 1 mm was used. The in-situ UV-vis tests were conducted for monitoring the chemical reaction rate (Supplementary Fig. 12). The reduced molecular catalysts were collected by reducing them inside a static cell (geometric area of the electrode is 2 cm^2) under 5 mA cm^{-2} with a cut-off voltage of 1.6 V. After disassembling the static cell, the sample was collected and mixed with K_2S_4 electrolyte. The quartz cell was well sealed inside the Ar-filled glovebox before taking the UV-vis test to exclude the possible oxidation from oxygen. For the *operando* UV-vis spectra, the quartz cell is connected with a flow cell with an active geometric area of 4 cm^2 (Supplementary Fig. 5). The electrolyte was injected inside the glovebox and carefully sealed before transferring to the outside of the glovebox. For obtaining the operando UV-vis spectra of molecular catalysts themselves (Supplementary Figs. 3-4 and Supplementary Figs. 5-7), the negolyte is 1 mM DHAQ in 1 M KOH, and the posolyte is 10 mL 0.25 M $K_4[Fe(CN_6)]$ + in 0.05 M $K_3[Fe(CN_6)]$ in 1 M KCl. The current density is 3 mA cm^{-2}. For the high-concentration molecular catalyst and K_2S_4 tests (Fig.3 and Fig.4), the posolyte composition for the operando UV-vis test is 15 mL 0.25 M $K_4[Fe(CN_6)]$ in 1 M KCl (theoretical capacity of 100.5 mAh), while the negolyte composition is 5 mM molecular catalysts, and 250 mM M K_2S_4 with or without molecular catalysts. The current density is 20 mA cm^{-2}.

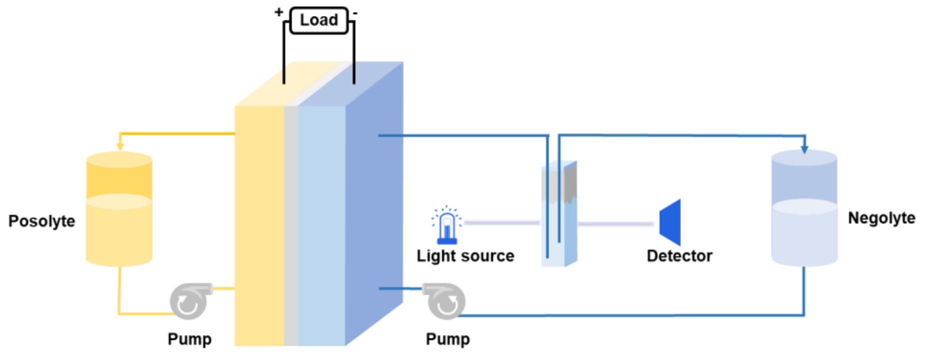

Supplementary Figure 5 | The schematic illustration of the *operando* UV-vis test.

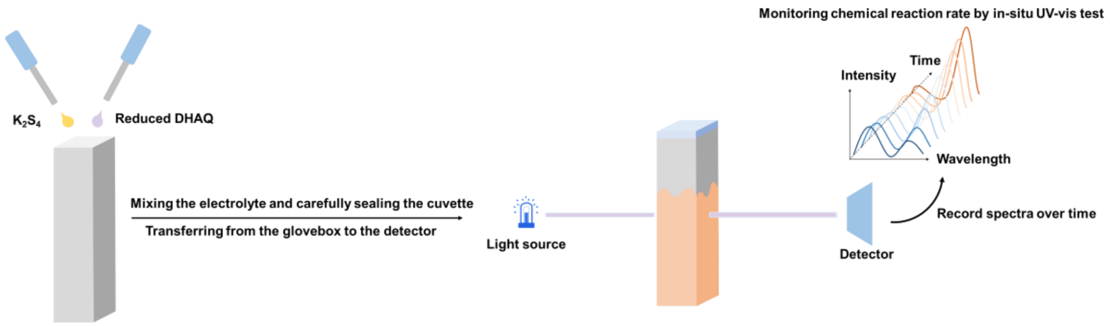

Supplementary Figure 12 | The schematic illustration of the in-situ UV-vis test to detect the chemical reaction rate.

Reviewer comments (3/8): For four-electrode test, do you mean that a reference electrode was included in both tanks? Please show the actual values of the measured potential differences in Fig. S42. Also please check the caption, as there concentration of K2S4 is mentioned as 250 mM or 1 M. The claim that main energy efficiency loss in this system is from the high resistance of Nafion 117 membrane is not well justified. The SCE-SCE potential allows directly measuring the Ohmic drop, so please use this value to evaluate what is the contribution of the membrane to the energy loss.

Author reply: We thank the reviewer's insightful comments, which allow us to clarify and improve.

Yes, the electrode was placed in the tank and inserted into the electrolyte, which has been shown to be an effective and simple method to decouple the potential in previous work (*Nat Commun* 13, 2388 (2022); *Nat Energy* 8, 1355–1364 (**2023**)). For better clarity, we provided the schematic illustration of the four-electrode test set-up. We provided the modified potential because of the overlap range between posolyte side and cross membrane potential, and we now also provide additional actual potential value vs. SCE for clarity. We have now checked the caption and corrected it to be 250 mM K_2S_4.

To study the effect of membrane resistance on the energy loss, we compare the cross-membrane potential difference and the potential drop caused by cell resistance. Since the resistance of the S-Fe cell with Nafion 117 is around 3.3 Ω cm^2 (*Nat Energy* 8, 1355–1364 (**2023**)), the ohmic loss is 66 mV at a current density of 20 mA cm^{-2}, which is highly consistent with our four-electrode test that the cross-membrane potential difference is around $\pm$70 mV. We have conducted iR correction analysis on the voltage profiles with or without relay catalysis to see the contribution of the membrane to the energy loss. For the cell with relay catalysts, the overpotential is greatly decreased after iR correction, while the high overpotential is not fully eliminated for the cell without catalysts, suggesting that the energy loss in catalysts-assisted cells is mainly from membrane resistance, but the poor kinetics of polysulfide is the main reason for high overpotential in catalyst-free cells.

We revised the manuscript on page 11:

The four-electrode test indicated that the relay catalysts decrease the negolyte overpotential, and the second plateau is from the lower working potential of the 2,6-DHAQ on the negolyte side (Supplementary Figs. 46-47). The iR correction was conducted to see the contribution of the energy loss in cells with or without relay catalysts. Interestingly, the cell with relay catalysts shows greatly decreased overpotential after removing the ohmic loss, while the high overpotential could not be fully eliminated from iR correction (Supplementary Fig. 48), suggesting the main energy efficiency loss in the catalyst-assisted system is from the high resistance of Nafion 117 membrane, but the poor polysulfide kinetics for catalysts-free system.

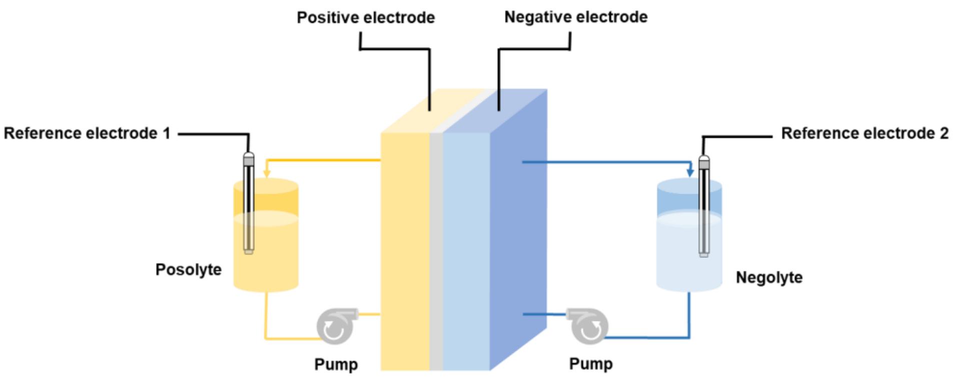

Supplementary Figure 46 | The schematic illustration of the four-electrode test.

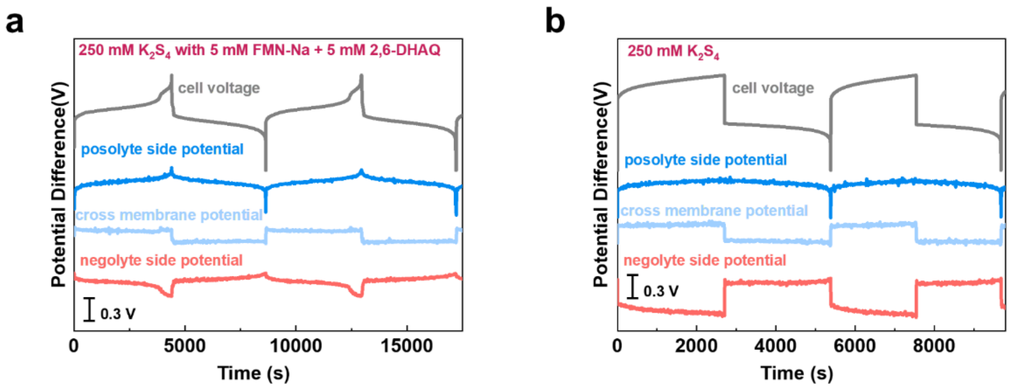

Supplementary Figure 47 | The four-electrode voltage profile of S-Fe flow cell (a) with relay catalysts and (b) without molecular catalysts at 20 mA cm^{-2} (10 mL of 250 mM K_2S_4 with or without 5 mM FMN-Na + 5 mM 2,6-DHAQ–1 M KOH |Nafion 117 membrane| 15 mL of 0.25 M $K_4[Fe(CN)_6]$–1 M KCl).

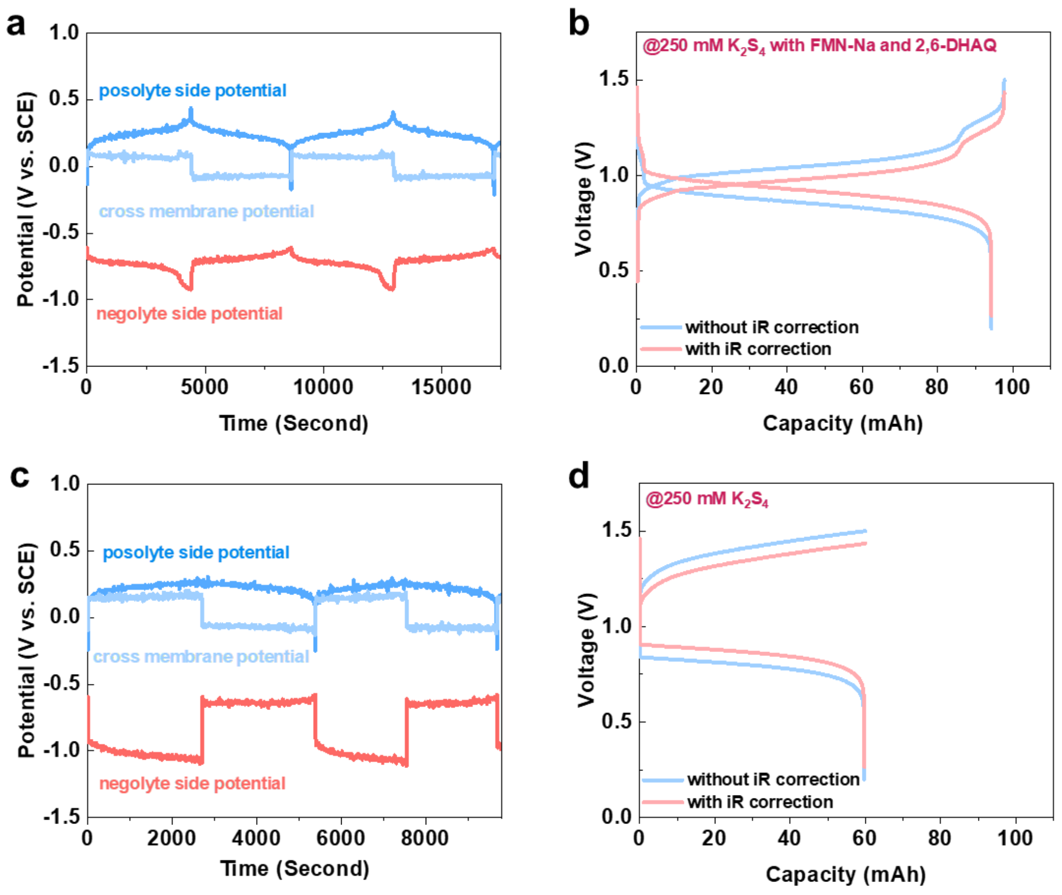

Supplementary Figure 48 | (a) The four-electrode voltage profile (vs. SCE) of the S-Fe flow cell with relay catalysts. (b) The iR correction for the -Fe flow cell with relay catalysts. (c) The four-electrode voltage profile (vs. SCE) of the S-Fe flow cell without relay catalysts. (d) The iR correction for the -Fe flow cell without relay catalysts. The resistance of the Nafion 117 membrane in the S-Fe flow cell[5] is around 3.3 Ω cm^2.

Reviewer comments (4/8): What is the energy efficiency of the 100 cm2 stack, and how does it compare to smaller cell?

Author reply: We thank the reviewer's insightful comments, which allow us to clarify and improve.

The energy efficiency is 73.5% at 30 mA cm^{-2} and 61.3% at 50 mA cm^{-2} for the 100 cm^2 flow cell stack, which is quite similar to the results in small cells (70.3% at 30 mA cm^{-2}). We provide the efficiency of the 100 cm^2 stack in Supplementary information, and we believed theses small discrepancies may come from the flow field, membrane, flow rate, and operation temperatures. These results verified that our relay strategy still functions well in the increased-size cell.

We revised the manuscript on page 16:

The energy efficiency of the flow cell stack is 73.5% at 30 mA cm^{-2} and 61.3% at 50 mA cm^{-2}, which is comparable with the EE in 12 cm^2 small cells (Supplementary Fig. 68).

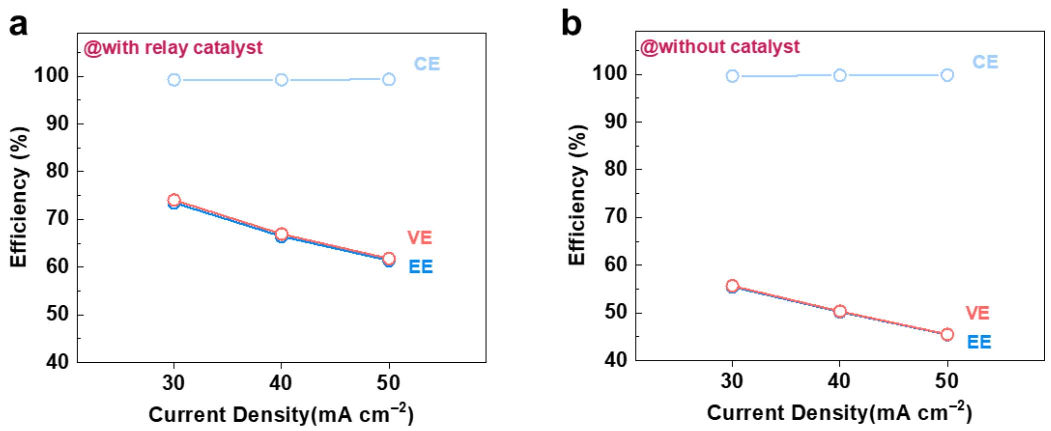

Supplementary Figure 68 | (a) The CE, EE, and VE of the 100 cm^2 S-Fe flow cell stack with relay catalysts from 30 to 50 mA cm^{-2}. (b) The CE, EE, and VE of the 100 cm^2 S-Fe flow cell stack without catalysts from 30 to 50 mA cm^{-2}.

Reviewer comments (5/8): How are the capacity fade rates for the larger cell, and at different temperatures.

Author reply: We thank the reviewer's insightful comments, which allow us to clarify and improve.

The capacity fade rate of the 100 cm^2 cell is 0.08% per cycle. However, we found the electrolyte leakage issue during the cell operation, which is ascribed to the main reason for faster capacity decay. Such engineering optimization will be further conducted in the future for large flow cell stacks. For the high-temperature test at 40 ℃, the capacity fade rate is 0.00047% per cycle at 60 mA cm^{-2} for 200 cycles.

We revised the manuscript on page 17:

Stable cycling of more than 200 cycles was achieved at 60 mA cm^{-2} with a low decay rate of 0.00047% per cycle.

Reviewer comments (6/8): It is stated that carbon felt was used as a current collector, but this is incorrect, as this is an electrode, while carbon plate is the current collector. What kind of pump and tubing were used, and what was the gasket material? How is the electrolyte introduced into the electrode, i.e. is there a flow channel in the carbon plate? Would be nice to have photos of the cell design in the supplementary material?

Author reply: We thank the reviewer's insightful comments, which allow us to clarify and improve.

We have corrected and modified the Methods part to clarify the flow cell structure. The flow cell is connected to two glass containers with peristaltic pump tubing (Tygon® Chemical) and PTFE tubes, and we applied the

peristaltic pump (Chuang Rui Precision Pump) to circulate the electrolyte. The gasket is made of PTFE. There are serpentine-type flow channels in the carbon plates, and we provided the photos of our cells in the supplementary material for better clarity.

We revised the manuscript on Methods on page 20:

Flow Cell assembly: The flow cell (Supplementary Fig. 35a) was constructed by using K^+-type Nafion membrane or CRIS membrane as the separator and carbon felts as the electrodes for both posolyte and negolyte side. The carbon felt was heated at 500 °C to improve its hydrophily. The flow cell is composed of stacked layers of carbon felt, carbon plate (with serpentine-type flow channels), and stainless-steel plate on each side (Supplementary Fig. 35b), sandwiching the membrane. The 3 mm PTFE gasket with O ring was adopted to prevent the electrolyte from leaking. The flow cell is connected to two glass containers with peristaltic pump tubing (Tygon® Chemical) and PTFE tubes. The peristaltic pump (Chuang Rui Precision Pump) was applied to circulate the electrolyte. The geometric area of the electrode is 12 cm^2 unless otherwise specified.

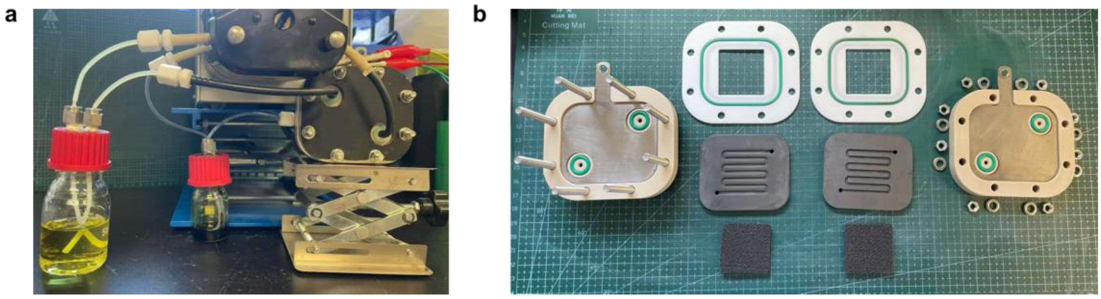

Supplementary Figure 35 | The optical images of (a) the S-Fe flow cell device and (b) cell components.

Reviewer comments (7/8): Eq. S10 is not properly balanced (there should be 2 S22-)

Author reply: We thank the reviewer's insightful comments, which allow us to clarify and improve.

We revised the supplementary materials:

This homogeneous electron transfer route converts the sluggish electrochemical process into a fast chemical process between reduced FMN^{5-} and S_4^{2-} (Eqn (S9-S10)).

$$FMN^{3-} + 2\,e^- \rightarrow FMN^{5-} \qquad (S9)$$

$$FMN^{5-} + S_4^{2-} \rightarrow FMN^{3-} + 2\,S_2^{2-} \qquad (S10)$$

Reviewer comments (8/8):

COMSOL simulations

The COMSOL simulations are very unrealistic, so they should be completely removed or done properly with a 2D electrode, as COMSOL example for vanadium. At the moment it does not bring any added value to the work, and I feel that the simulation could be a follow-up work. In the current simulation the electrode potential varies along the electrode, mass transport does not represent the experimental flow cell work, and kinetic expressions are unrealistic.

For COMSOL simulations, negative half-cell and tank should be considered, but for simplicity, membrane and the positive side could be ignored and membrane-electrode interface can be just considered a source or sink of K+. The tank should be considered as homogenous reactions between polysulfide and mediator can also take place in the tank. Here, the tank could be assumed as ideally stirred reactor. The convection should also be modelled in the cell.

Nernst-Planck equations are valid only for dilute electrolytes, and correct treatment of such complicated electrolytes is very challenging, so this could be mentioned.

As I understand the set-up, the felt electrode is pressed against the carbon plate, so why would there be a diffusion layer in the COMSOL model? Or is there a flow channel in the plate? Why is the thickness of the diffusion layer 50 um and electrode 100 um? It seems that the geometry was adopted from ref. 8, but this does not make any sense as there the electrode was on the bottom, and it was approached with a microelectrode from the solution, without any convection.

Why the catalytic processes (S18 and S19) are considered irreversible? The correct way would be to derive an equilibrium constant from equation S5 and then evaluate the forward and backward reactions from equilibrium constant and UV/Vis data, as K = kf/kb (or other way depending on how K is defined).

Why take only cathodic Tafel equation and not consider the full Butler-Volmer? This is incorrect, as the potential range is such that both the anodic and cathodic branch should be considered. Also polysulfide reactions at the electrode are not considered.

The boundary conditions and domain equation for current flow required for Nernst-Planck equation are not given.

Author reply: We thank the reviewer's insightful comments, which allow us to clarify and improve.

We fully agree with the reviewer that the 1D model has its own limitations and is far from reality. We have removed this part.

Here we address the comments of Reviewer #4 in detail:

The authors have designed relay-catalysts to improve the capacity and stability of polysulfide-ferrocyanide-based RFBs. It is comprehsive works which includes mechanistic study, molecular modelling, electrochemical characterization, and multi-physics simulations. It suits Nat. Com. well. Nevertheless, I have the following two points that should be clarified by the authors during the revisions.

Author reply: We deeply thank the reviewer's encouraging comments and insightful suggestions for us to improve our work.

Reviewer comments (1/3): For aqueous RFBs, the energy density is usually limited by the solubility. How does the reply catalysis change the solubility of polysulfide-ferrocyanide?

Author reply: We thank the reviewer's insightful comments, which allow us to clarify and improve.

Since the relay catalysts are only added in a low concentration at the mM level, we didn't observe their effects on the concentration of polysulfide. We provided the optical images of polysulfide electrolyte at high concentration (1 M and 1.2 M) to verify that the small amount of organic molecules would not greatly affect the solubility of polysulfide species even approaching the solubility limit of polysulfide species(Fig. R1 below). The molecular catalysts are only added in the negolyte side, so it will not affect the concentration of ferrocyanide as they are separated by a cation-exchange membrane.

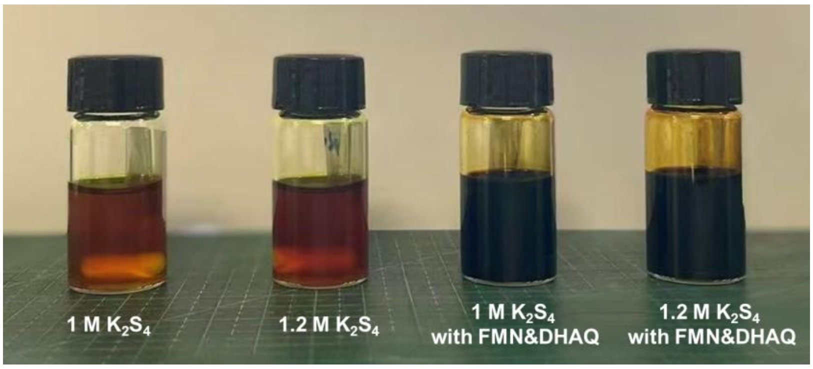

Fig. R1 | The optical images of 1 M K_2S_4 and 1.2 M K_2S_4 electrolyte with or without relay catalysts (50 mM FMN-Na + 50 mM 2,6-DHAQ).

Reviewer comments (2/3): I understand that the system with FMN-Na and 2,6-DHAQ can last over 500 cycles. But does this depend on the concentration of FMN-Na and 2,6-DHAQ? In other words, how stable are these relay-catalysts? What can happen is that a high concentration of relay-catalysts masks their instability, so the RFBs can last as long as these relay-catalysts are not yet decomposed.

Author reply: We thank the reviewer's insightful comments, which allow us to clarify and improve.

The concentration of molecular catalysts would affect the achieved capacity and energy efficiency. We conducted additional experiments to study the concentration effects. When the concentration is too low (2 mM), the relay catalysts could achieve a high capacity utilization at a low current density (10 mA cm^{-2}), but their insufficient kinetics at high current density (40 mA cm^{-2}) lead to low capacity and EE. The achieved capacity is boosted with improved energy efficiency when concentration is increased from 2 mM to 10 mM and then 50 mM, and such an effect is more pronounced at higher current density.

The catalyst stability will definitely determine the capacity and energy efficiency in the polysulfide flow cells because the loss of catalysts will lead to decreased capacity and EE. However, our results of high capacity retention and well-maintained EE all verified a high stability of relay catalysts. We further divide the charging capacity achieved below 1.3 V as FMN-catalyzed capacity and the charging capacity achieved above 1.3V as DHAQ-catalyzed capacity, which are all highly related to catalyst concentration. During the 2689 hours of operations, the ratio of FMN-catalyzed capacity and DHAQ-catalyzed capacity is almost unchanged, also

suggesting a good stability of relay catalysts in our system. Since the catalyst effects are more pronounced at high current density with low catalyst concentration lead to low capacity, we further conducted the long-term cycling test at 40 mA cm^{-2}, which still shows a high stability over 500 cycles (56 days). These results all synergistically verify the high stability of relay catalysts in our systems.

We revised the manuscript on page 14:

Since the achieved capacity and energy efficiency are highly related to the catalyst concentration (Supplementary Figs. 56-57), the well-maintained discharge capacity and EE (Fig. 5c and Supplementary Fig. 58) verified the high stability of relay catalysts. Furthermore, the capacity contribution from FMN-Na catalysis and 2,6-DHAQ catalysis is almost unchanged, suggesting a high activity of relay catalysts over 112 days (Supplementary Figs. 59). Even at a high current density of 40 mA cm^{-2}, the cell still shows a high stability with little capacity decay over 500 cycles with nearly full capacity utilization (Supplementary Figs. 60).

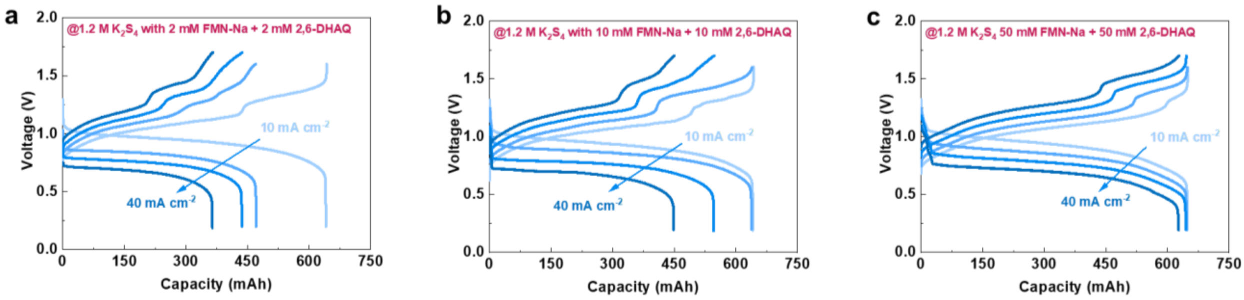

Supplementary Figure 56 | The voltage profiles of 1.2 M S-Fe flow cells with (a) 2 mM FMN-Na + 2 mM 2,6-DHAQ, (b) 10 mM FMN-Na + 10 mM 2,6-DHAQ, and (c) 50 mM FMN-Na + 50 mM 2,6-DHAQ from 10 to 40 mA cm^{-2} (10 ml of 1.2 M K_2S_4 with FMN-Na and 2,6-DHAQ–1 M KOH |Nafion 117 membrane| 24 ml of 1 M ferrocyanide posolyte (0.5 M $K_4[Fe(CN)_6]$ + 0.5 M $Na_4[Fe(CN)_6]$.

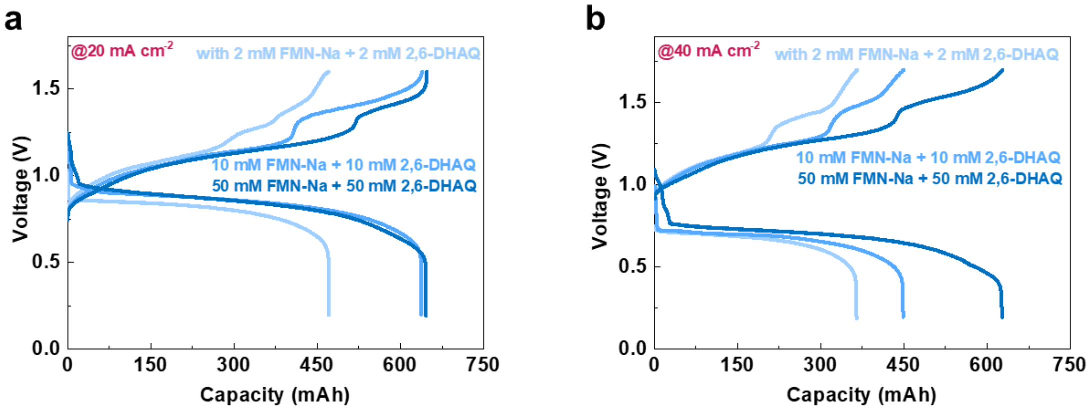

Supplementary Figure 57 | The voltage profiles of 1.2 M S-Fe flow cells with different concentrations of relay catalysts at (a) 20 mA $cm^{-2}$and (b) 40 mA cm^{-2}.

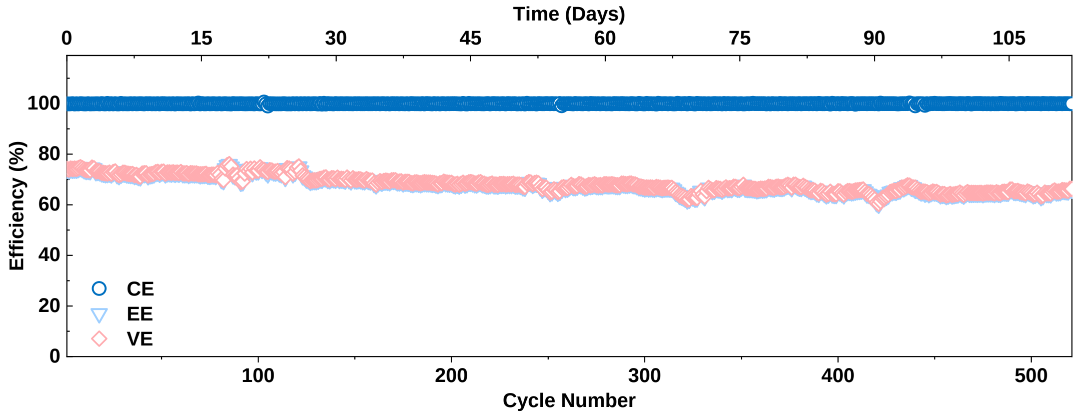

Supplementary Figure 58 | The CE, EE, and VE of the 1.2 M S-Fe flow cell.

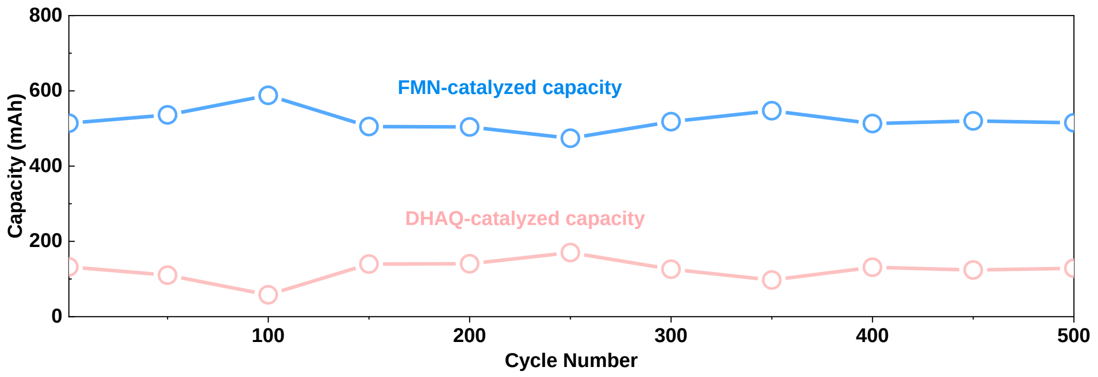

Supplementary Figure 59 | The capacity contribution from two charging plateaus over 500 cycles. The charging capacity below 1.3 V is ascribed to FMN-catalyzed capacity, and the charging capacity above 1.3 V is ascribed to DHAQ-catalyzed capacity. The small fluctuations are ascribed to temperature change over the 112 days.

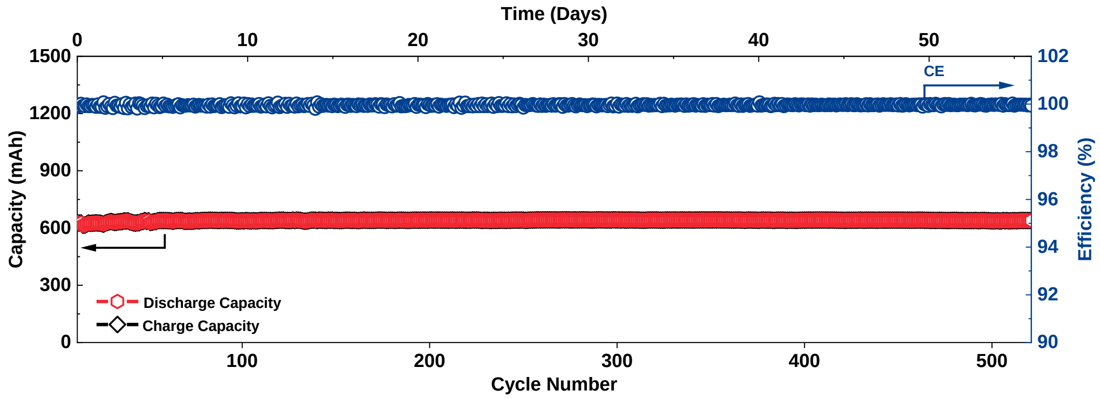

Supplementary Figure 60 | The long-term cycling stability of high-concentration S-Fe flow cell at 40 mA cm^{-2} (10 ml of 1.2 M K_2S_4–1 M KOH with 50 mM FMN-Na and 50 mM 2,6-DHAQ |CRIS membrane| 24 ml of 1 M ferrocyanide posolyte (0.5 M $K_4[Fe(CN)_6]$ + 0.5 M $Na_4[Fe(CN)_6]$).

Reviewer comments (3/3): For the COMSOL simulation, there is no list of parameters for the reaction-diffusion flow. This makes the work unreproducible. Therefore, a table that lists all physical parameters used in the COMSOL simulations should be provided in SI.

Author reply: We thank the reviewer's insightful comments, which allow us to improve. We have provided the physical parameters in the original Supplementary Table 4. Nevertheless, after considering the advice from Reviewer #3 to remove the COMSOL part, we have removed the COMSOL part in the current manuscript.

Here we address the comments of Reviewer #1 in detail:

The authors have adequately addressed the comments by this reviewer and significantly improved the manuscript. I recommend to accept this work at its present form.

Author reply: We deeply thank the reviewer's encouraging comments and insightful suggestions for us to improve our work.

Here we address the comments of Reviewer #2 in detail:

I am satisfied with the revised manuscript and recommend its acceptance in the current form

Author reply: We deeply thank the reviewer's encouraging comments and insightful suggestions for us to improve our work.

Here we address the comments of Reviewer #3 in detail:

The authors have adressed all the comments by the reviewers in a satisfactory manner, and I beleieve that the work can be accepted for publication.

Author reply: We deeply thank the reviewer's encouraging comments and insightful suggestions for us to improve our work.

Here we address the comments of Reviewer #4 in detail:

The authors have revised the manuscript sufficiently in response to the referees' comments. The computational aspects (DFT calculations) are also robust. Therefore, I recommend it for publication.

Author reply: We deeply thank the reviewer's encouraging comments and insightful suggestions for us to improve our work.
